# A draft genome assembly of halophyte *Suaeda aralocaspica*, a plant that performs C_4_ photosynthesis within individual cells

**DOI:** 10.1093/gigascience/giz116

**Published:** 2019-09-12

**Authors:** Lei Wang, Ganglong Ma, Hongling Wang, Chao Cheng, Shuyong Mu, Weili Quan, Li Jiang, Zhenyong Zhao, Yu Zhang, Ke Zhang, Xuelian Wang, Changyan Tian, Yi Zhang

**Affiliations:** 1 State Key Laboratory of Desert and Oasis Ecology, Xinjiang Institute of Ecology and Geography, Chinese Academy of Sciences, 818 South Beijing Road, Urumqi 830011, China; 2 University of Chinese Academy of Sciences, No.19(A) Yuquan Road, Shijingshan District, Beijing 100049, China; 3 Center for Genome Analysis, ABLife Inc., 388 Gaoxin 2nd Road, Wuhan, Hubei 430075, China; 4 Central Lab, Xinjiang Institute of Ecology and Geography, Chinese Academy of Sciences, 818 South Beijing Road, Urumqi 830011, China; 5 Key Laboratory of Biogeography and Bioresource in Arid Land, Xinjiang Institute of Ecology and Geography, Chinese Academy of Sciences, 818 South Beijing Road, Urumqi 830011, China

**Keywords:** *Suaeda aralocaspica*, genome, single-cell C_4_, photosynthesis, long noncoding RNAs, halophyte

## Abstract

**Background:**

The halophyte *Suaeda aralocaspica* performs complete C_4_ photosynthesis within individual cells (SCC_4_), which is distinct from typical C_4_ plants, which require the collaboration of 2 types of photosynthetic cells. However, despite SCC_4_ plants having features that are valuable in engineering higher photosynthetic efficiencies in agriculturally important C_3_ species such as rice, there are no reported sequenced SCC_4_ plant genomes, limiting our understanding of the mechanisms involved in, and evolution of, SCC_4_ photosynthesis.

**Findings:**

Using Illumina and Pacific Biosciences sequencing platforms, we generated ∼202 Gb of clean genomic DNA sequences having a 433-fold coverage based on the 467 Mb estimated genome size of *S. aralocaspica*. The final genome assembly was 452 Mb, consisting of 4,033 scaffolds, with a scaffold N50 length of 1.83 Mb. We annotated 29,604 protein-coding genes using Evidence Modeler based on the gene information from *ab initio* predictions, homology levels with known genes, and RNA sequencing–based transcriptome evidence. We also annotated noncoding genes, including 1,651 long noncoding RNAs, 21 microRNAs, 382 transfer RNAs, 88 small nuclear RNAs, and 325 ribosomal RNAs. A complete (circular with no gaps) chloroplast genome of *S. aralocaspica* 146,654 bp in length was also assembled.

**Conclusions:**

We have presented the genome sequence of the SCC_4_ plant *S. aralocaspica*. Knowledge of the genome of *S. aralocaspica* should increase our understanding of the evolution of SCC_4_ photosynthesis and contribute to the engineering of C_4_ photosynthesis into economically important C_3_ crops.

## Background

Carbon loss through photorespiration and water loss through transpiration are common in C_3_ plants, especially in warm or dry environments, and they result in significant decreases in growth, water use efficiency, and harvestable yields [1]. These problems are overcome in C_4_ and crassulacean acid metabolism (CAM) plant families [2], which perform evolved CO_2_-concentrating mechanisms (C_4_ cycle) and Calvin cycle (C_3_ cycle) using spatial (Kranz structure) and temporal (day to night switch) separations, respectively. Both C_4_ and CAM plants can outperform C_3_ plants, especially under photorespiratory conditions, and increase their water use efficiency [2], which has created considerable interest in implementing the C_4_ cycle in C_3_ crops such as rice to improve yields and stress tolerance [3–6].

Among eudicots, C_4_ photosynthesis most frequently occurs in the Amaranthaceae of Caryophyllales [7–9]. Four Amaranthaceae species (3 *Bienertia* and 1 *Suaeda*) can perform both C_4_ and C_3_ cycles within individual photosynthetic cells (single-cell C_4_[SCC_4_]) [10–13]. *Suaeda* contains species that utilize all types of C_4_, C_3_, and SCC_4_ mechanisms for CO_2_ fixation and, thus, represents a unique genus to study the evolution of C_4_ photosynthesis [14]. Mechanistically, the spatially separated chloroplasts in SCC_4_ contain different sets of nuclear-encoded proteins that are related to specific functions in the C_4_ and C_3_ cycles, which biochemically and functionally resemble mesophyll and bundle sheath cells in chloroplasts of Kranz C_4_ plant species [10, 11, 15–18]. These findings indicate that the key enzymes in photosynthesis are conserved and that both C_3_ and C_4_ enzymes work in the same cells in SCC_4_ plants during the daytime, which is different from both C_4_ and CAM plants.

At present, most of the knowledge of SCC_4_ photosynthesis has come from studies of *Bienertia sinuspersici*, which has 2 types of chloroplasts distributed in the central and peripheral parts of the cell [16, 18–29]. Studies on *Suaeda aralocaspica* (NCBI:txid224144) have focused on the germination of dimorphic seeds [30–34]. *S. aralocaspica* has elongated photosynthetic cells with 2 types of chloroplasts distributed at the opposite ends of the cell. This is analogous to the Kranz anatomy but lacks the intervening cell wall [35]. This cellular feature indicates that *S. aralocaspica* conducts C_4_ and C_3_ photosynthesis within a single cell, perhaps retaining the photosynthetic characteristics of both C_4_ and C_3_ cycles and representing an intermediate model of the evolutionary process from C_3_ to C_4_ [35,36]. *S. aralocaspica* is a hygro-halophyte that grows in temperate salt deserts with low night temperatures in areas ranging from the northeast of the Caspian lowlands eastwards to Mongolia and western China [35]. Therefore, it is important to sequence the genome of *S. aralocaspica*, which should aid the study of C_4_ evolution under stressful growth conditions and accelerate the engineering of C_4_ photosynthesis into C_3_ crops for adaptation to high-saline growth conditions.

In the present study, we sequenced the genome of *S. aralocaspica* collected from a cold desert in the Junggar Basin, Xinjiang, China. Using an integrated assembly strategy that combined shotgun Illumina sequencing and single-molecule real-time sequencing technology from Pacific Biosciences (PacBio), we generated a reference genome assembly of *S. aralocaspica* using protocols established in other plant species [37–40]. To our knowledge, this is the first sequenced SCC_4_ genome. These genomic resources provide a platform for advancing basic biological research and gene discovery in SCC_4_ plants, as well as for engineering C_4_ functional modules into C_3_ crops to increase yields and to adapt to high-salt conditions.

## Data Description

### Plant material

**Figure 1:**
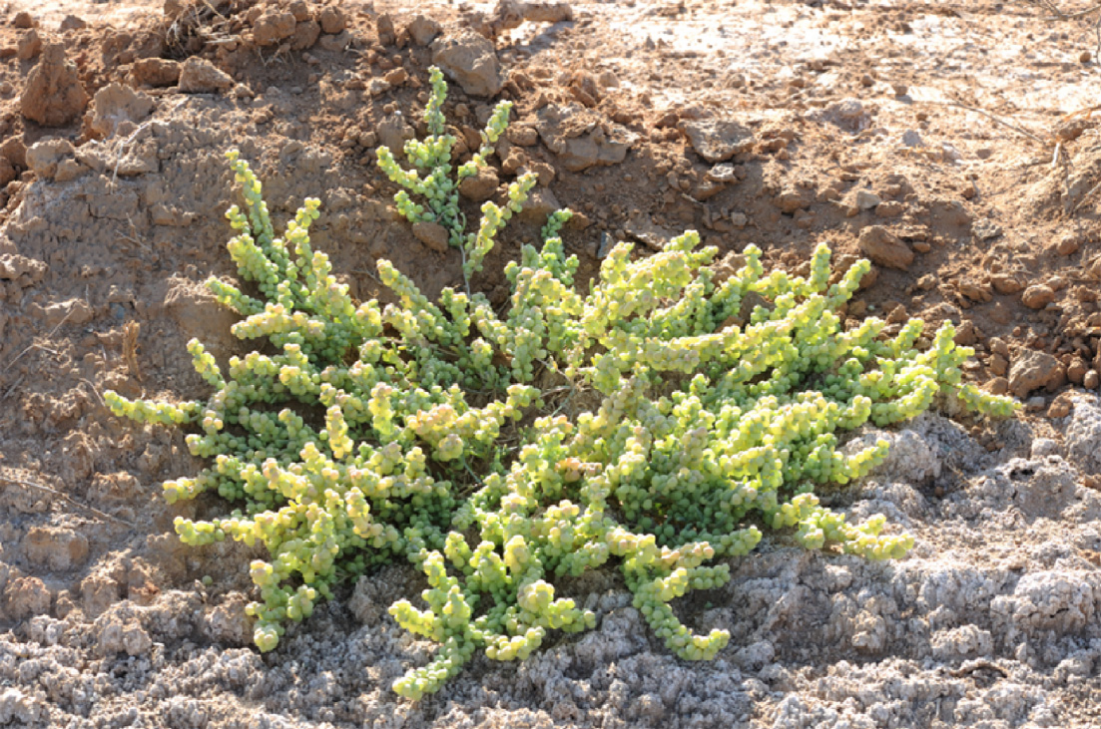
Example of *S. aralocaspica*.

Seeds were first collected from a healthy specimen of *S. aralocaspica* (Fig. 1). The selected plant measured ∼40 cm in height and was located within a natural stand close to Fu-kang County, Xinjiang Uygur Autonomous Region, China (44 14 N latitude, E 87 40 E longitude, 445 m elevation). The seeds were placed in 0.1% potassium permanganate, washed clean for 5 min with ultrapure water, and then spread in sterilized petri dishes. After a week of 30°C shaded culturing, the seeds germinated. After seed germination, leaves were collected as tissue sources for whole-genome sequencing. In addition, 6 other healthy *S. aralocaspica* (collected from the same location as the plant used for seed collection) were chosen as tissue (mature leaf, stem, root, and fruit) sources for RNA sequencing (RNA-seq). The samples were frozen in liquid nitrogen immediately after being collected and then stored at −80°C until DNA/RNA extraction. All the samples were collected with permission from and under the supervision of the local forestry bureau.

### DNA extraction and genome sequencing

Genomic DNA was extracted from leaves using a General AllGen Kit (Tiangen Biotech, Beijing, China) according to its manufacturer's instructions. Genomic DNA isolated from *S. aralocaspica* was used to construct multiple types of libraries, including short insert size (350, 500, and 800 bp) libraries, mate-paired (2, 5, 10, and 20 kb) libraries, and PacBio single-molecule real-time cell libraries. The purified libraries were quantified and stored at −80°C before sequencing. Then, the *S. aralocaspica* genome was sequenced on Illumina HiSeq 2000 (Illumina Inc., San Diego, CA, USA) and PacBio RS II platform (Pacific Biosciences of California, Menlo Park, CA, USA) using 8 libraries with different insert sizes. This generated 370 Gb raw Illumina HiSeq data and 10 Gb (∼21× genome coverage) PacBio reads (Supplemental Table 1).

To reduce the effects of sequencing errors on the assembly, a series of stringent filtering steps were used during read generation. We cleaned Illumina reads using the following steps: (1) Cut off adaptors. For the mate-paired library data, reads without Nextera adaptors longer than 10 bp on both end1 and end2 were removed; (2) Remove tail bases with quality score <20; (3) Remove reads harboring >20% bases with quality scores <20; (4) Remove reads with lengths <30 nucleotides (nt) for DNA-seq; and (5) Remove duplicated paired-end reads from DNA-seq that represent potential PCR artifacts. In total, 1053,309 raw subreads were produced by Pacbio. Then, reads with lengths <1 kb were filtered, and 935,509 reads were retained. Next, 46  Gb of Illumina clean reads with 100-bp read lengths was used to correct the PacBio raw reads using Proovread (Proovread, RRID:SCR_017331) [41] (v2). This yielded 632,805 corrected PacBio reads. After the quality control and filtering steps, 195 Gb clean Illumina reads and 6.9 clean PacBio reads were retained, resulting in a 433× fold coverage of the genome (Supplemental Table 1).

### Estimation of genome size

GCE (GCE, RRID:SCR_017332) [42] (v1.0.0) was used to estimate the genome size and heterozygosity. The term *k*-mer refers to a sequence with a length of*k* bp, and each unique *k*-mer within a genome dataset can be used to determine the discrete probability distributions of all possible *k*-mers and their frequencies of occurrence. Genome size can be calculated using the total length of sequencing reads divided by sequencing depth. To estimate the sequencing depth of the *S. aralocaspica* genome, we counted the copy number of a certain *k*-mer (e.g., 17-mer) present in the sequence reads and plotted the distribution of the copy numbers. The peak value of the frequency curve represents the overall sequencing depth. We used the algorithm *N* × (*L* − *K* + 1)/*D* = *G*, where *N* represents the total sequence read number, *L* represents the average length of sequence reads, and *K* represents the *k*-mer length, which was defined here as 17 bp. *G* denotes the genome size, and *D* represents the overall depth estimated from the *k*-mer distribution. Based on this method, the estimated genome size of *S. aralocaspica* was 467 Mb (Supplemental Fig. 1) and the heterozygosity was 0.16%.

### Genome assembly

The primary assembled genome was generated by SOAPdenovo (SOAPdenovo2, RRID:SCR_014986) [43] (version 2.04-r240) and contained 17,302 initial contigs (N50, ∼49.2 kb) and 4,184 scaffolds (N50, ∼1.44 Mb) spanning 445.6 Mb, with 96.1 Mb (21.56%) of the total size being intra-scaffold gaps (Supplemental Table 2). Then, we used all of the reads from the short insert libraries to fill gaps using GapCloser (GapCloser, RRID:SCR_015026) [44] (v1.12), and 74.7% of the total gaps were filled. This resulted in a genome size of 424.5 Mb, with 5.92% gaps, which was calculated using the total length of Ns divided by the total length of the assembly. Then, PBJelly (PBJelly, RRID:SCR_012091) [45] (v15.8.24) was used for the second round of gap filling using the polished PacBio data. This finally yielded a ∼452-Mb genome assembly with 4,033 scaffolds (N50, 1.83 Mb) (Table 1, Supplemental Table 2). The assembly spanned 96.8% of the *S. aralocaspica* genome (467 Mb) estimated by the *k*-mer spectrum (Supplemental Fig. 1).

**Table 1.** Summary of *S. aralocaspica* genome assembly

Assembly	Illumina	Illumina + PacBio
Total assembly size	424 Mb	452 Mb
Number of scaffolds (≥500 bp)	4,184	4,033
Longest scaffold	9.29 Mb	9.98 Mb
N50 contig (size/number)	49.21 kb/2,464	
N50 scaffold (size/number)	1.44 Mb/80	1.83 Mb/67
N90 scaffold (size/number)	306.62 kb/332	363.87 kb/282
% of N	5.78%	2.98%
Annotation		
Number of protein-coding genes		29,604
Number of small RNAs		816
Number of long noncoding genes		1,982

### RNA preparation and sequencing

RNA-seq was performed for genome annotation. Different tissues (mature leaf, stem, root, and fruit) of 6 *S. aralocaspica* specimens were used for RNA extraction. Tissues were ground in liquid nitrogen. After homogenizing the samples in a guanidine thiocyanate extraction buffer, sodium acetate and chloroform/isoamyl alcohol (24:1) were added. The solution was shaken vigorously, placed on ice for 15 min, and centrifuged (13,200 rpm) at 4°C to separate a clear upper aqueous layer, from which RNA was precipitated with isopropanol. The precipitated RNA was washed with 75% ethanol to remove impurities and then resuspended with diethyl pyrocarbonate–treated water. Total RNA was treated with RQ1 DNase (Promega) to remove DNA. The quality and quantity of the purified RNA were determined by measuring the absorbance at 260 nm/280 nm (A260/A280) using smartspec plus (BioRad). RNA integrity was further verified by 1.5% agarose gel electrophoresis. RNAs were then equally mixed for RNA-seq library preparation. Polyadenylated messenger RNAs (mRNAs) were purified and concentrated with oligo(dT)-conjugated magnetic beads (Invitrogen) before directional RNA-seq library preparation. Purified mRNAs were fragmented at 95°C, followed by end repair and 5′ adaptor ligation. Reverse transcription was performed using an RT primer harboring a 3′ adaptor sequence and a randomized hexamer. The complementary DNAs (cDNAs) were purified and amplified, and PCR products corresponding to 200–500 bp were purified, quantified, and stored at −80°C before sequencing. Transcriptomic libraries were sequenced using Illumina HiSeq X Ten (Illumina Inc., San Diego, CA, USA) for paired-end 150-nt reads. As a result, we generated 30 Gb of RNA-seq data (Supplemental Table 3).

To further annotate transcriptional start and termination sites, we also sequenced cap analysis of gene expression and deep sequencing (CAGE) and polyadenylation site sequencing (PAS) data. In brief, 20 μg of total RNA of mature leaves was used for CAGE-seq library preparation. Polyadenylated mRNAs were purified and concentrated with oligo (dT)-conjugated magnetic beads (Invitrogen). After treating with FastAP (Invitrogen) for 1 h at 37°C and subsequently with tobacco acid pyrophosphatase (Ambion) for 1 h at 37°C, the decapped full-length mRNA was ligated to the Truseq 5′ RNA adaptor (Illumina) for 1 h at 37°C and purified with oligo (dT)-conjugated magnetic beads (Invitrogen). Following fragmentation at 95°C, first-strand cDNA was synthesized using an RT primer harboring the Truseq 3′ adaptor sequence (Illumina) and a randomized hexamer. The cDNAs were purified and amplified using Truseq PCR primers (Illumina), and products corresponding to 200–500 bp were purified, quantified, and stored at −80°C until sequencing. CAGE-seq libraries were sequenced with Illumina Nextseq 500 (Illumina Inc., San Diego, CA, USA) for paired-end 150-nt reads. Finally, 16 Gb of CAGE-seq data were generated (Supplemental Table 3). In addition, 10 μg of total RNA of mature leaves was used for PAS-seq library preparation. In brief, polyadenylated mRNAs were purified using oligo (dT)-conjugated magnetic beads (Invitrogen). Purified RNA was fragmented and then reverse transcription was performed using a PAS-RT primer (a modified Truseq 3′ adaptor harboring dT18 and 2 additional anchor nucleotides at the 3′ terminus). DNA was then synthesized with Terminal-Tagging oligo cDNA using a ScriptSeq™cv2 RNA-Seq Library Preparation Kit (Epicentre). The cDNAs were purified and amplified, and PCR products corresponding to 300–500 bp were purified, quantified, and stored at −80°C before sequencing. PAS-seq libraries were sequenced with Illumina Nextseq 500 (Illumina Inc., San Diego, CA, USA) for single-end 300-nt reads. Finally, 28.5 Gb of PAS-seq data were generated (Supplemental Table 3).

To annotate microRNA, a total of 3 μg of mixed total RNA was the template for a small RNA cDNA library preparation using Balancer NGS Library Preparation Kit for small/microRNA (GnomeGen), following the manufacturer's instructions. Briefly, RNAs were ligated to 3′ and 5′ adaptors sequentially, reverse transcribed to cDNA, and PCR amplified. The whole library was applied to 10% native polyacrylamide gel electrophoresis, and bands corresponding to microRNA insertions were cut and eluted. After ethanol precipitation and washing, the purified small RNA libraries were quantified using a Qubit Fluorometer (Invitrogen) and stored at −80°C until sequencing. The small RNA library was sequenced with Illumina GA IIx (Illumina Inc., San Diego, CA, USA) for 33-nt reads. Finally, 4.5 Gb of small RNA data were generated (Supplemental Table 3).

### Genome quality evaluation

Different methods and data were used to check the completeness of the assembly. Using BWA (BWA, RRID:SCR_010910) [46], we found that 87.08–90.63% of DNA-paired end reads (350, 500, and 800 bp) could be properly mapped to the final assembled genome (Supplemental Table 4, Supplemental Fig. 2). We evaluated the completeness of the gene regions in our assembly using BUSCO (BUSCO, RRID:SCR_015008) [47] (v3.0.2). In total, 89.5% of the 1,440 single-copy orthologs presented in the plant lineage was completely identified in the genome (Supplemental Fig. 3).

Furthermore, Trinity (Trinity, RRID:SCR_013048) [48] (r20140413p1) was used to assemble the RNA-seq reads sequenced from the mixed *S. aralocaspica* RNA library into 157,521 unigenes. Then, these unigenes were aligned to the genome assembly by BLASTN with default parameter. We found that 94.5% of the unigenes could be aligned to the genome assembly, and 76.3% of the unigenes could cover 90% of the sequence length of 1 scaffold. For unigenes longer than 1 kb, 99.5% of the unigenes could be aligned to the genome assembly, and 92.8% of the unigenes could cover 90% of the sequence length of 1 scaffold (Supplemental Table 5).

### Gene and functional annotations

The genome of *S. aralocaspica* was annotated for protein-coding genes (PCGs), repeat elements, noncoding genes, and other genomic elements. In detail, MAKER (MAKER, RRID:SCR_005309) [49] (v2.31.9) was used to generate a consensus gene set based on 3 different types of evidence, *ab initio*, protein homologues, and the transcripts. *De novo* predictions were processed by AUGUSTUS (AUGUSTUS, RRID:SCR_008417) [50] (v3.2.1). Nonredundant protein sequences of 7 sequenced plants (*Arabidopsis thaliana, Oryza sativa, Beta vulgaris, Chenopodium quinoa, Glycine max, Spinacia oleracea*, and *Vitis vinifera*) provided homology evidence. The *S. aralocaspica* RNA-seq data generated from this study and a published transcriptome of the seed [51] were assembled into unigenes by Trinity [48] as the transcript evidence. We predicted 29,064 PCGs, with an average transcript length of 4,462 bp, coding sequence size of 1,112 bp, and a mean of 4.76 exons per transcript (Supplemental Tables 6 and 7). Of the annotated PCGs, 97.2% were functionally annotated by the InterPro, GO, KEGG, SwissProt, or NR databases (Supplemental Figs 4 and 5, Supplemental Table 8), and ∼91% were annotated with protein or transcript support (Supplemental Table 9). The transcriptional start and termination sites of most of the annotated genes were supported by sequencing reads from CAGE-seq and PAS-seq (Supplemental Figs 6 and 7).

In addition, 1,651 long noncoding RNAs were predicted following a previously published method [52]. In total, 382 transfer RNAs (tRNAs) were predicted using tRNAscan-SE (tRNAscan-SE, RRID:SCR_010835) [53] (v1.3.1). Additionally, 21 miRNAs, 88 small nuclear RNAs, and 325 ribosomal RNAs were identified by using the CMscan tool from INFERNAL (Infernal, RRID:SCR_011809) [54] (v1.1.2) to search the Rfam database with option –cut_ga (Supplemental Table 10, Supplemental Fig. 8).

### Repeat annotation

To annotate the repeat sequences of the *S. aralocaspica* genome, a combination of *de novo* and homology-based approaches was used [55,56]. For homology-based identification, we used RepeatMasker (RepeatMasker, RRID:SCR_012954) [57] (open-4.0.5) to search the protein database in Rebase against the *S. aralocaspica* genome and identify transposable elements (TEs). The Rebase database [58] was used to identify TEs. Parameters of RepeatMasker were set to “-species Viridiplantae -pa 30 -e rmblast”. In the *de novo* approach, PILER (PILER, RRID:SCR_017333) [59] (v1.0) was used to build the consensus repeat database. PILER software requires PALS, FAMS, and PILER to construct the consensus library. The default parameters of PILER were used. Then, the predicted consensus TEs were classified using RepeatClassifer implemented in the RepeatModeler package (RepeatModeler, RRID:SCR_015027) [60] (Version 1.0.11). We used RepeatMasker to search the TEs within the database constructed by PILER. Finally, we combined the *de novo* and homolog predictions of repeat elements according to their coordination in the genome, and detected 173.5 Mb repeat elements, which constituted 38.41% of the genome (Supplemental Table 11). As observed in other sequenced genomes [61], long terminal repeats [62] in *S. aralocaspica* occupied the majority (48.5%) of the repeated sequences (Supplemental Table 12).

### Phylogenetic placement of *S. aralocaspica*

The OrthoFinder (OrthoFinder, RRID:SCR_017118) [63] (v2.3.3) clustering method was used to perform orthologous group analyses with complete annotated protein sequences of 18 sequenced plant genomes: 8 C_3_ species (*Solanum tuberosum, S. oleracea, B. vulgaris, C. quinoa, A. thaliana, O. sativa, Musa acuminata*, and *Physcomitrella patens*), 8 C_4_ species (*S. aralocaspica, Amaranthus hypochondriacus, Sorghum bicolor, Setaria italica, Zea mays, Saccharum* spp., *Panicum hallii*, and *Pennisetum glaucum*), and 2 CAM species (*Ananas comosus* and *Phalaenopsis equestris*). The longest proteins encoded by each gene in all species were selected as input for OrthoFinder with default parameters. In total, 19,324 orthogroups, containing ≥2 genes, were circumscribed, 11,768 of which contained ≥1 gene from *S. aralocaspica* (Supplemental Table 13). Of the 29,604 annotated *S. aralocaspica* genes, 23,112 (89%) were classified into orthogroups. In total, 3,895 orthogroups (172,107 genes) were shared among all the genomes analyzed. A total of 70 orthogroups (351 genes) were specific to the assembled *S. aralocaspica* genome when compared with the other 17 genomes.

**Figure 2:**
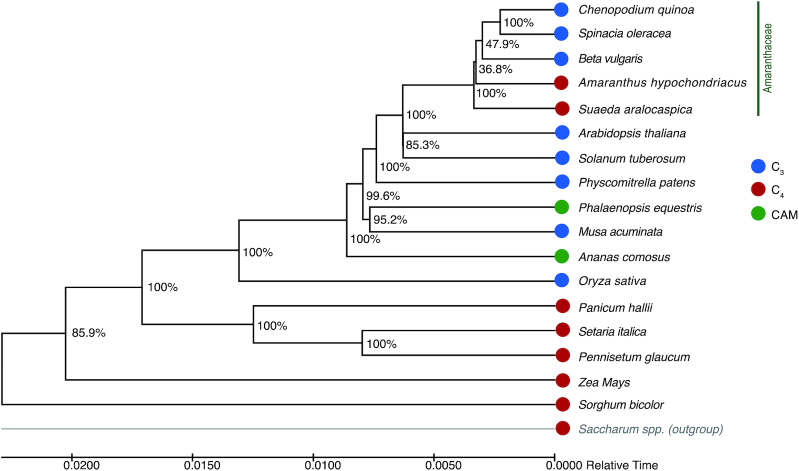
Phylogenetic tree of *S. aralocaspica* with other C_3_/C_4_/CAM plants. Bootstrap values were obtained from 1,000 bootstrap replicates and are reported as percentages.

With OrthoFinder, 15 single-copy orthologous genes, shared across 18 species, were identified and were aligned with MUSCLE (MUSCLE, RRID:SCR_011812) [58] (v3.8.31), using default settings (see Supplementary File 1 for commands and settings). The concatenated amino acid sequences were trimmed using trimAI (trimAI, RRID:SCR_017334) [64] (trimal -gt 0.8 -st 0.001 -cons 60) (v1.2rev59) and were further used by ModelFinder to select the best model (JTTDCMut+F+I+G4). Then, the phylogenetic trees were constructed using IQ-Tree (IQ-TREE, RRID:SCR_017254) [65] (v1.6.10). The aLRT method was used to perform 1,000 bootstrap analyses to test the robustness of each branch. Then, a timetree was inferred using the Realtime method [66,67] and ordinary least-squares estimates of branch lengths. This analysis involved 18 amino acid sequences. There were 4,489 positions in the final dataset. The timetree was constructed using MEGA X (MEGA Software, RRID:SCR_000667) [68]. The resulting phylogenetic tree showed that all 5 Amaranthaceae species were placed in the same clade, among which *A. hypochondriacus* (C_4_) was placed as a sister subclade to the other 3 C_3_ species (Fig. 2). Moreover, *S. aralocaspica* (SCC_4_) was the sister clade of 4 other species from the Amaranthaceae including *A. hypochondriacus* (C_4_) (Fig. 2). Our results of phylogenetic analyses were consistent with a previous study on the evolution of *C. quinoa* [69]. Inside of the Amaranthaceae, the close phylogenetic distance between *S. aralocaspica* (SCC_4_) and *A. hypochondriacus* (C_4_), away from all other C_3_ relatives, suggests that these SCC_4_ and C_4_ photosynthesizers might have had independently evolved. Outside of the Amaranthaceae, *S. aralocaspica* (SCC_4_) is more closely related to the C_3_ than C_4_ plants. These findings do not fully support the existing model that *S. aralocaspica* would be a C_3_–C_4_ intermediate and was on the road toward the C4 plants [35,36].

### Assembly of the *S. aralocaspica* chloroplast genome

**Figure 3:**
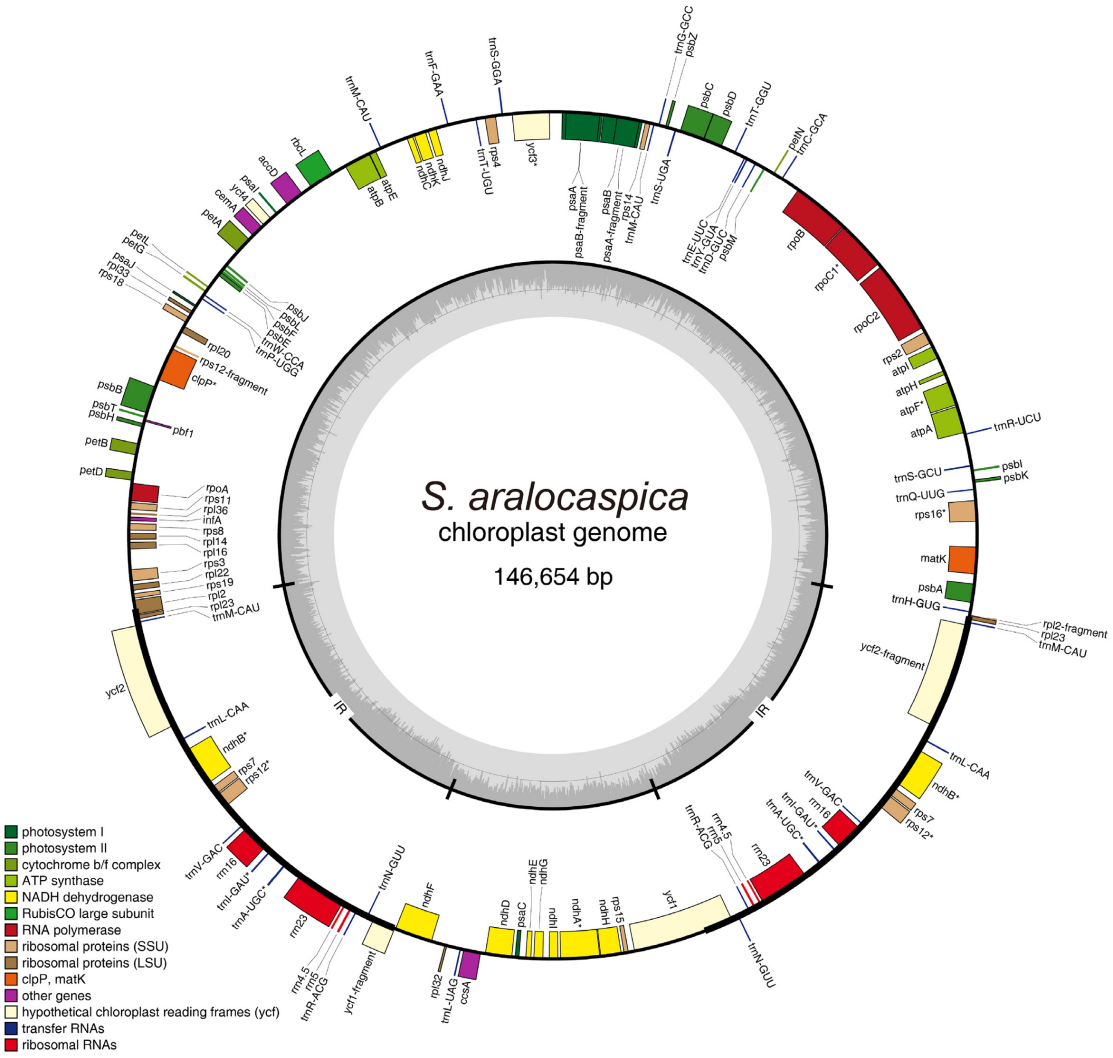
Gene map of the *S. aralocaspica* chloroplast genome. Genes shown outside the outer circle are transcribed clockwise, and those inside are transcribed counterclockwise. Genes belonging to different functional groups are color coded. The dashed area in the inner circle indicates guanine-cytosine content of the chloroplast genome.

Using the short insert size (350 bp) data, a complete (circular with no gaps) chloroplast genome of *S. aralocaspica* was assembled at 146,654 bp in length using NOVOPlasty (NOVOPlasty, RRID:SCR_017335) [70] (v2.7.2). The Rubisco-bis-phosphate oxygenase (RuBP) subunit of *C. quinoa* (GenBank:KY419706.1) was selected as a seed sequence. An initial gene annotation of the genome was performed using GeSeq (GeSeq, RRID:SCR_017336) [71]. The circular chloroplast genome maps were drawn using the OrganellarGenome DRAW tool (OGDraw, RRID:SCR_017337) [72], with subsequent manual editing (Fig. 3).

## Conclusion

Using the Illumina and Pacbio platforms, we successfully assembled the genome of *S. aralocaspica*, the first sequenced genome of a SCC_4_ plant. The final genome assembly was 452 Mb in size and consisted of 4,033 scaffolds, with a scaffold N50 length of 1.83 Mb. We annotated 29,604 protein-coding genes and noncoding genes including 1,651 long noncoding RNAs, 21 miRNAs, 382 tRNAs, 88 small nuclear RNAs, and 325 ribosomal RNAs. The phylogenetic tree placed SCC_4_ in a clade more closely related to the C_3_ than the C_4_ plants, not fully supporting the hypothesis that SCC_4_ is a C_3_–C_4_ intermediate that independently evolved from the C_3_ ancestors. A complete (circular with no gaps) chloroplast genome of *S. aralocaspica* was also assembled, and was 146,654 bp in size. The available genome assembly, together with transcriptomic data of *S. aralocaspica*, provides a valuable resource for investigating C_4_ evolution and mechanisms. We anticipate that future studies of *S. aralocaspica* will greatly facilitate the process of engineering crops, especially C_3_ species, including rice, with higher photosynthetic efficiencies and saline tolerance.

## Availability of supporting data and materials

Raw sequencing data are deposited in the NCBI SRA with accession number SRP128359. The NCBI Bioproject accession is PRJNA428881. Further supporting data and materials are available in the *GigaScience* GigaDB database [73].

## Additional files

**Supplemental Figure 1:** *k*-mer distribution of sequencing reads.

**Supplemental Figure 2:** Size distribution of inserts in sequenced paired-end DNA reads.

**Supplemental Figure 3:** Integrity comparison of genome assemblies of *S. aralocaspica* with BUSCO. For *S. aralocaspica*, assemblies in each step were analyzed respectively.

**Supplemental Figure 4:** Annotated genes supported by different evidence.

**Supplemental Figure 5:** Gene ontology distribution of *S. aralocaspica* protein-coding genes.

**Supplemental Figure 6:** Transcription start site (TSS) annotation with CAGE-seq.

**Supplemental Figure 7:** Transcription terminal site (TTS) annotation with PAS-seq.

**Supplemental Figure 8:** Noncoding RNAs classification in *S. aralocaspica*.

**Supplemental Table 1:** Summary of sequencing data obtained for genome assembly.

**Supplemental Table 2:** The assembly statistics of the *S. aralocaspica* genome.

**Supplemental Table 3:** Information of different types of RNA libraries.

**Supplemental Table 4:** Mapping efficiency of short insert library reads

**Supplemental Table 5:** Assessment of sequence coverage of *S. aralocaspica* genome assembly using unigenes.

**Supplemental Table 6:** Gene prediction in the *S. aralocaspica* genome.

**Supplemental Table 7:** Comparison of the gene structure among *S. aralocaspica* and some other species.

**Supplemental Table 8:** Summary of *S. aralocaspica* gene annotation based on homology or functional classification.

**Supplemental Table 9:** Number of *S. aralocaspica* genes with protein or unigene support.

**Supplemental Table 10:** Noncoding RNA genes in the *S. aralocaspica* genome.

**Supplemental Table 11:** Repeat elements in the *S. aralocaspica* genome. Repeat elements were identified by different methods and then combined into a final repeat set.

**Supplemental Table 12:** Repeat elements in *S. aralocaspica* genome.

**Supplemental Table 13:** Orthogroups clustered by OrthoFinder in 18 species.

giz116_GIGA-D-19-00024_Original_Submission

giz116_GIGA-D-19-00024_Revision_1

giz116_GIGA-D-19-00024_Revision_2

giz116_Response_to_Reviewer_Comments_Original_Submission

giz116_Response_to_Reviewer_Comments_Revision_1

giz116_Reviewer_1_Report_Original_Submission Robert van Buren -- 3/19/2019 Reviewed

giz116_Reviewer_2_Report_Original_Submission John Lovell -- 4/9/2019 Reviewed

giz116_Reviewer_2_Report_Revision_1 John Lovell -- 7/24/2019 Reviewed

giz116_Supplemental_Figures_and_Tables

## Abbreviations

bp: base pairs; BUSCO: Benchmarking Universal Single-Copy Orthologs; BWA: Burrows-Wheeler Aligner; CAGE: cap analysis of gene expression and deep sequencing; CAM: crassulacean acid metabolism; cDNA: complementary DNA; Gb: gigabase pairs; GCE: Genomic Character Estimator; GO: Gene Ontology; kb: kilobase pairs; KEGG: Kyoto Encyclopedia of Genes and Genomes; Mb: megabase pairs; miRNA: microRNA; mRNA: messenger RNA; NCBI: National Center for Biotechnology Information; nt: nucleotide; PacBio: Pacific Biosciences; PAS: polyadenylation site sequencing; PCG: protein-coding gene; RNA-seq: RNA sequencing; SCC_4_: single-cell C_4_ photosynthesis; SRA: Sequence Read Archive; TE: transposable element; tRNA: transfer RNA; TTS: transcription terminal site.

## Competing interests

The authors declare that they have no competing interests.

## Funding

This research was supported by the Key Research and Development Program of Xinjiang province (2018B01006–4), the National Natural Science Foundation of China (31770451), the National Key Research and Development Program (2016YFC0501400), and ABLife ( ABL2014–02028).

## Authors' contributions

C.T., L.W., Yi Z., and S.M. initiated the project and designed the study. L.W., H.W., L.J., Z.Z., and K.Z. prepared experimental materials and performed experiments for data collection. G.M., C.C., Yu Z., H.W., L.J., and K.Z. assembled the genome, analyzed the data, and generated the graphs. Yi Z., W.Q., C.T., L.W., C.C., and X.W. wrote the manuscript.
