# Supplementary material for: A draft genome assembly of halophyte Suaeda aralocaspica, a plant that performs C4 photosynthesis within individual cells
Source: Gigascience. 2019 Sep 12;8(9):giz116. doi: 10.1093/gigascience/giz116 (PMC6741815; doi:10.1093/gigascience/giz116)
Supplement: giz116_GIGA-D-19-00024_Original_Submission [file giz116_giga-d-19-00024_original_submission.pdf]

GigaScience

A draft genome assembly of halophyte Suaeda aralocaspica, the first reference genome of single-cell C4 plant

--Manuscript Draft--

**Manuscript Number:**	GIGA-D-19-00024
**Full Title:**	A draft genome assembly of halophyte Suaeda aralocaspica, the first reference genome of single-cell C4 plant
**Article Type:**	Data Note
**Funding Information:**	National Natural Science Foundation of China (31770451) — Dr. Lei Wang<br>National Key Research and Development Program (2016YFC0501400) — Prof. Changyan Tian<br>ABLife (ABL2014-02028) — Dr. Yi Zhang
**Abstract:**	Background<br><br>The halophyte Suaeda aralocaspica performs a complete C4 photosynthesis within individual cells (SCC4), which is distinct from the typical C4 plants that require the collaboration of two types of photosynthetic cells. However, despite the valuable features of SCC4 in the process of engineering higher photosynthetic efficiency in C3 species including rice, there is no reported genome sequence for SSC4 plants yet, which limits the understanding of mechanism and evolution of SCC4 photosynthesis.<br><br>Findings<br><br>Using the Illumina and Pacbio platforms, we generated a total of ~205 Gb cleaned genomic DNA sequences with 191-fold coverage based on the 467-Mb estimated genome size of S. aralocaspica. The final genome assembly is 452 Mb and consisted of 4033 scaffolds with a scaffolds N50 length of 1.83 Mb. We annotated 29604 protein-coding genes using Evidence Modeler based on the gene information from ab initio prediction, protein homology with known genes, and transcriptome evidence of RNA-seq. We also annotated noncoding genes including 1, 651 long noncoding RNA (lncRNA), 21 miRNAs, 382 transfer RNAs (tRNAs), 88 small nuclear RNAs, 325 ribosomal RNAs (rRNAs). Complete (circular with no gaps) chloroplast (cp) genomes of S. aralocaspica was also assembled to be 146,654 in length.<br><br>Conclusions<br><br>We have presented the first genome sequence of SCC4 plants. Knowledge in the genome of S. aralocaspica should provide more opportunities for understanding SCC4 photosynthesis evolution and contribute to the engineering of C4 photosynthesis to economic C3 crops.
**Corresponding Author:**	Yi Zhang, Ph.D.<br><br>CHINA
**Corresponding Author Secondary Information:**	
**Corresponding Author's Institution:**	
**Corresponding Author's Secondary Institution:**	
**First Author:**	Lei Wang
**First Author Secondary Information:**	
**Order of Authors:**	Lei Wang

Powered by Editorial Manager® and ProduXion Manager® from Aries Systems Corporation

	Ganglong Ma
	Hongling Wang
	Chao Cheng
	Shuyong Mu
	Weili Quan
	Li Jiang
	Zhenyong Zhao
	Yu Zhang
	Ke Zhang
	Xuelian Wang
	Changyan Tian
	Yi Zhang, Ph.D.
**Order of Authors Secondary Information:**	
**Additional Information:**	
**Question**	**Response**
Are you submitting this manuscript to a special series or article collection?	No
**Experimental design and statistics**<br><br>Full details of the experimental design and statistical methods used should be given in the Methods section, as detailed in our Minimum Standards Reporting Checklist. Information essential to interpreting the data presented should be made available in the figure legends.<br><br>Have you included all the information requested in your manuscript?	Yes
**Resources**<br><br>A description of all resources used, including antibodies, cell lines, animals and software tools, with enough information to allow them to be uniquely identified, should be included in the Methods section. Authors are strongly encouraged to cite Research Resource Identifiers (RRIDs) for antibodies, model organisms and tools, where possible.	Yes

Powered by Editorial Manager® and ProduXion Manager® from Aries Systems Corporation

Have you included the information requested as detailed in our Minimum Standards Reporting Checklist?	
**Availability of data and materials**<br><br>All datasets and code on which the conclusions of the paper rely must be either included in your submission or deposited in publicly available repositories (where available and ethically appropriate), referencing such data using a unique identifier in the references and in the “Availability of Data and Materials” section of your manuscript.<br><br>Have you have met the above requirement as detailed in our Minimum Standards Reporting Checklist?	Yes

Powered by Editorial Manager® and ProduXion Manager® from Aries Systems Corporation

Manuscript
Click here to access/download;Manuscript;manuscript-final-for-gigascience.docx
Click here to view linked References

A draft genome assembly of halophyte *Suaeda aralocaspica*, the first reference genome of single-cell C_4 plant

Lei Wang[1], Ganglong Ma[2], Hongling Wang[3], Chao Cheng[2], Shuyong Mu[3], Weili Quan[2], Li Jiang[4,5], Zhenyong Zhao[1], Yu Zhang[2], Ke Zhang[1], Xuelian Wang[2], Changyan Tian[1,*], Yi Zhang[2,*]

[1]State Key Laboratory of Desert and Oasis Ecology, Xinjiang Institute of Ecology and Geography, Chinese Academy of Sciences, Urumqi 830011, China,

[2]Center for Genome Analysis, ABLife Inc., Wuhan, Hubei 430075, China,

[3]Central Lab, Xinjiang Institute of Ecology and Geography, Chinese Academy of Sciences, Urumqi 830011, China,

[4]Key Laboratory of Biogeography and Bioresource in Arid Land, Xinjiang Institute of Ecology and Geography, Chinese Academy of Sciences, Urumqi 830011, China,

[5]Turpan Eremophytes Botanical Garden, Chinese Academy of Sciences, Turpan 838008, China,

[*]Correspondence address: Yi Zhang, Center for Genome Analysis, ABLife Inc., Wuhan, Hubei 430075, China, E-mail: yizhang@ablife.cc; Changyan Tian, State Key Laboratory of Desert and Oasis Ecology, Xinjiang Institute of Ecology and Geography, Chinese Academy of Sciences, Urumqi 830011, China, E-mail: tianchy@ms.xjb.ac.cn

Abstract

Background: The halophyte *Suaeda aralocaspica* performs a complete C_4 photosynthesis within individual cells (SCC_4), which is distinct from the typical C_4 plants that require the collaboration of two types of photosynthetic cells. However, despite the valuable features of SCC_4 in the process of engineering higher photosynthetic efficiency in C_3 species including rice, there is no reported genome sequence for SSC_4 plants yet, which limits the understanding of mechanism and evolution of SCC_4 photosynthesis.

Findings: Using the Illumina and Pacbio platforms, we generated a total of ~205 Gb cleaned genomic DNA sequences with 191-fold coverage based on the 467-Mb estimated genome size of *S. aralocaspica*. The final genome assembly is 452 Mb and consisted of 4033 scaffolds with a scaffolds N50 length of 1.83 Mb. We annotated 29604 protein-coding genes using Evidence Modeler based on the gene information from ab initio prediction, protein homology with known genes, and transcriptome evidence of RNA-seq. We also annotated noncoding genes including 1, 651 long noncoding RNA (lncRNA), 21 miRNAs, 382 transfer RNAs (tRNAs), 88 small nuclear RNAs, 325 ribosomal RNAs (rRNAs). Complete (circular with no gaps) chloroplast (cp) genomes of *S. aralocaspica* was also assembled to be 146,654 in length.

Conclusions: We have presented the first genome sequence of SCC_4 plants. Knowledge in the genome of *S. aralocaspica* should provide more opportunities for understanding SCC_4 photosynthesis evolution and contribute to the engineering of C_4 photosynthesis to economic C_3 crops.

Keywords: *Suaeda aralocaspica*, genome, single-cell C_4, photosynthesis, stress tolerance, lncRNAs, halophyte

Background

Carbon loss through photorespiration and water loss through transpiration are common in C_3 plants especially in warm or dry environments which result in significant decrease in growth, water-use efficiency, and harvestable yields [1]. These problems are overcome in C_4 and CAM plant families [2], which perform evolved CO_2-concentrating mechanisms (C_4 cycle) and Calvin cycle (C_3 cycle) by spatial (Kranz structure) and temporal separation (day to night switch), respectively. Both C_4 and CAM plants can outperform C_3 photosynthesis especially under photorespiratory conditions and increase the water usage efficiency [2], which has created considerable interests in implementing a C_4 cycle into C_3 crops such as rice to improve yields and stress tolerance recently [3-6].

Among eudicots, C_4 photosynthesis most frequently occurs in Amaranthaceae of Caryophyllales [7-9]. Four Amaranthaceae species (three *Bienertia* and one *Suaeda*) can perform both C_4 cycle and C_3 cycle within individual photosynthetic cells (single-cell C_4, SCC_4) [10-13]. *Suaeda* contains species utilizing all types of C_4, C_3 and SCC_4 mechanisms for CO_2 fixation, and thus representing a unique genus to study the evolution of C_4 photosynthesis [14]. Mechanistically, it has been shown that the spatial separated chloroplasts in SCC_4 contain different sets of nuclear encoded proteins which are to related specific functions in the C_4 and C_3 cycle, which resembles the biochemistry and function of mesophyll cell (MC) and bundle sheath cell (BSC) chloroplasts of Kranz C_4 plant species [10, 11, 15-18]. These findings indicate

that the key enzymes in photosynthesis are conserved, and both C_3 and C_4 enzymes work in the same cell in SCC_4 plants during the day time, which is different from both C_4 and CAM plants.

Up to now, most knowledge on single-cell C_4 photosynthesis comes from the studies of cellular and species *B. sinuspersici* with two types chloroplast distributing in the central and peripheral parts of the cell [16, 18-29]. Studies on *S. aralocaspica* have been focused on germination of dimorphic seeds [30-34]. *S. aralocaspica* has elongated photosynthesis cells with two types of chloroplasts distributing at the opposite ends of a cell, which is analogous to the Kranz anatomy, but lacking the intervening cell wall [35]. This cellular feature indicates that *S. aralocaspica* conducts C_4 and C_3 photosynthesis within a single cell, perhaps retaining the photosynthesis characteristics of both C_4 cycle and C_3 cycle and representing an intermediate model of the evolution process from C_3 to C_4 [35, 36]. *S. aralocaspica* is a hygro-halophyte growing in temperate salt deserts with low night temperatures in the areas from northeast of the Caspian lowlands east to Mongolia and western China [35]. Therefore, it is essential to sequence the genome of *S. aralocaspica*, which may help to study the C_4 evolution in stressful growth conditions which is expected to accelerate the progress of engineering C_4 photosynthesis into C_3 crops for adaption of the high salt growth condition.

In the present study, we sequenced the genome of *S. aralocaspica* collected from cold desert of the Junggar Basin, Xinjiang, China. Using an integrated assembly strategy combining shotgun Illumina and single-molecule real-time (SMRT) sequencing technology from Pacific Biosciences (PacBio), we generated a reference genome assembly of *S. aralocaspica* using established protocols in other plant species [37-40]. To our knowledge, this is the first SCC_4 genome. These genomic resources provide a platform for advancing basic biology research and

gene discovery in SCC_4, as well as for engineering C_3 crops with a C_4 function module to increase the yield and high-salt adaption.

Data Description

Plant material and DNA/RNA extraction

Mature leaves of *Suaeda aralocaspica* (Figure 1) were collected from Fu-kang, China. The sample was frozen in liquid nitrogen immediately after being collected and then stored at -80 °C until DNA extraction. Genomic DNA was extracted from samples using General AllGen Kit (Tiangen) according to its manufacture's instruction. Genomic DNA isolated from *S. aralocaspica* was used to construct multiple types of libraries, including short insert size (350bp, 500bp and 800bp) libraries, mate-paired (2 kb, 5 kb, 10 kb and 20 kb) libraries and PacBio SMRT Cell libraries. The purified libraries were quantified and stored at -80℃ before sequencing.

Genome sequencing and size estimation

The *S. aralocaspica* genome was sequenced by Illumina sequencing platform (HiSeq 2000) and PacBio RS II platform using 8 libraries with different insert sizes. This generated 370 Gb raw Illumina HiSeq data and 10 Gb (~21 × genome coverage) PacBio data. (Supplemental Table 1).

To reduce the effect of sequencing error to the assembly, a series of stringent filtering steps were taken on reads generation. We cleaned Illumina reads by the following steps: (1) Cut off adaptors. For the mate-pair library data, reads without Nextera adaptor longer than 10bp in both end1 and end2 were removed. (2) Remove tail bases with quality score less than 20. (3) Remove reads harboring more than 20% bases with quality score less than 20. (4) Reads with a length

less than 30 nt for DNA-seq reads were removed. (5) Remove duplicated paired-end reads from DNA-seq that represent potential polymerase chain reaction artefacts. After the quality control and filtering steps, 195 Gb cleaned Illumina reads and 10 Gb PacBio reads were retained with a fold coverage of genome was about 191 × (Supplemental Table 1).

GCE [41] was used to estimate the genome size and heterozygosity. K-mer refers to a sequence with the length of k bp, and each unique k-mer within a genome dataset can be used to determine the discrete probability distributions of all possible k-mers and their frequency of occurrence. Genome size could be calculated using the total length of sequencing reads divided by sequencing depth. To estimate the sequencing depth of *S. aralocaspica* genome, we counted the copy number of a certain k-mer (e.g., 17-mer) present in sequence reads, and plotted the distribution of copy numbers. The peak value of the frequency curve represents the overall sequencing depth. We used the algorithm: $N \times (L - K + 1)/D = G$, where N is the total sequence read number, L is the average length of sequence reads and K is k-mer length, defined as 17 bp here. G denotes the genome size, and D is the overall depth estimated from k-mer distribution. Based on the method, the estimated genome size of *S. aralocaspica* was 467 Mb (Supplemental Figure 1) and heterozygosity was 0.16%.

Genome assembly

Primary assembly genome was generated by SOAPdenovo[42] (version2.04-r240), which comprises 17,302 initial contigs (N50 ~ 49.2 kb) and 4,184 scaffolds (N50 ~ 1.44 Mb) spanning 445.6 Mb with 96.1 Mb (21.56%) of the total size were intra-scaffold gaps (Supplemental Table 2). Then we used all of the reads from short insert libraries to fill gaps with GapCloser [43], and 74.7% of the total gaps were filled, resulting a genome size spanning 424.5 Mb with 5.92%

gaps. Then PBJelly [44] (v15.8.24) was used for the second round of gap filling with approximately 21× of PacBio data. This finally yielded a ~452M genome assembly with 4033 scaffolds (N50 =1.83Mb) (Table 1, Supplemental Table 2). The assembly spanned 96.8% of the *S. aralocaspica* genome (467 Mb) estimated by the kmer-spectrum (Supplemental Figure 1).

Table 1: Summary of *S. aralocaspica* genome assembly.

**Assembly**	**Illumina**	**Illumina+PacBio**
**Total assembly Size**	424 Mb	452 Mb
**Number of scaffolds (≥ 500 bp)**	4184	4033
**Longest scaffold**	9.29 Mb	9.98 Mb
**N50 contig (size/number)**	49.21 kb/2464	-
**N50 scaffold (size/number)**	1.44 Mb/80	1.83 Mb/67
**N90 scaffold (size/number)**	306.62 kb/332	363.87 kb/282
**% of N**	5.78%	2.98%
**Annotation**		
**Number of protein coding genes**	-	29604
**Number of small RNAs**	-	816
**Number of long non-coding genes**	-	1982

RNA preparation and sequencing

RNA-seq was performed for genome annotation. Different tissues (mature leaf, stem, root, fruit, growing root and seedling) of *S. aralocaspica* were collected for RNA extraction. Tissues were

pulverized by fine grinding using liquid nitrogen. After homogenizing the sample with guanidine thiocyanate extraction buffer, NaAc and chloroform/isoamyl alcohol (24:1) was added. The solution was shaken vigorously, placed on ice for 15 min, and centrifuged at 4 °C to separate into a clear upper aqueous layer, from which RNA was precipitated with isopropanol. The precipitated RNA was washed with 75% ethanol to remove impurities, and then resuspended with DEPC-treated water. Total RNA was treated with RQ1 DNase (Promega) to remove DNA. The quality and quantity of the purified RNA were determined by measuring the absorbance at 260 nm/280 nm (A260/A280) using smartspec plus (BioRad). RNA integrity was further verified by 1.5% agrose gel electrophoresis. RNAs were then balance mixed for RNA-seq library preparation. Polyadenylated mRNAs were purified and concentrated with oligo(dT)-conjugated magnetic beads (Invitrogen) before directional RNA-seq library preparation. Purified mRNAs were fragmented at 95℃ followed by end repair and 5' adaptor ligation. Reverse transcription was performed with RT primer harboring 3' adaptor sequence and randomized hexamer. The cDNAs were purified and amplified and PCR products corresponding to 200-500 bps were purified, quantified and stored at -80℃ before sequencing. Transcriptomic libraries were sequenced with Hiseq X Ten for paired-ends 150 nt reads. As a result, we generated 30 Gb of RNA sequencing (RNA-seq) data (Supplemental Table 3).

To further annotate transcription start sites and transcription termination sites, we also sequenced CAGE (Cap Analysis of Gene Expression and deep sequencing) and PAS (Polyadenylation Site sequencing) data. In brief, 20 μg of total RNA of mature leaf was used for CAGE-seq library preparation. In brief, polyadenylated mRNAs were purified and concentrated with oligo (dT)-conjugated magnetic beads (Invitrogen). After treated with

FastAP (Invitrogen) at 37℃ for 1h and subsequently with Tobacco Acid Pyrophosphatase (Ambion) at 37 ℃ for 1h, the decapped full length mRNA was ligated to Turseq 5' RNA adaptor (Illumina) at 37℃ for 1h, and purified with oligo (dT)-conjugated magnetic beads (Invitrogen). Following fragmentation at 95℃, first strand cDNA was synthesized with RT primer harboring Turseq 3' adaptor sequence (Illumina) and a randomized hexamer. The cDNAs were purified and amplified with Truseq PCR primers (Illumina) and products corresponding to 200-500 bps were purified, quantified and stored at -80℃ until sequencing. CAGE-seq librarie were sequenced with Illumina Nextseq 500 for paired-end 150 nt reads. Finally, we generated 16 Gb of CAGE-seq data (Supplemental Table 3). 10 μg of total RNA of mature leaf was used for PAS-seq library preparation. In brief, polyadenylated mRNAs were purified with oligo (dT)-conjugated magnetic beads (Invitrogen). Purified RNA was fragmented, and then reverse transcription was per-formed with PAS-RT Primer (a modified Truseq 3' adaptor harboring dT18 and two additional anchor nucleotides at the 3' terminus) , then synthesized DNA with Termi-nal-Tagging oligo cDNA using ScriptSeq™cv2 RNA-Seq Library Preparation Kit (Ep-icentre). The cDNAs were purified and amplified and PCR products corresponding to 300-500 bps were purified, quantified and stored at -80°C before sequencing. PAS-seq libraries were sequenced with Illumina Nextseq 500 for single-end 300 nt reads. Finally, we generated 28.5 Gb of PAS-seq data (Supplemental Table 3).

To annotate miRNA, a total of 3 μg of mixed total RNA was used for small RNA cDNA library preparation with Balancer NGS Library Preparation Kit for small/microRNA (GnomeGen) following manufacture's instruction. Briefly, RNAs were ligated to 3' and 5' adaptor sequentially, reverse transcribed to cDNA and PCR amplified. Whole library was

applied to 10% native PAGE gel electrophoresis and bands corresponding to microRNA insertion were cut and eluted. After ethanol precipitation and washing, the purified small RNA libraries were quantified with Qubit Fluorometer (Invitrogen) and stored at -80℃ until sequencing. Small RNA library was sequenced with Illumina GA IIx for 33 nt reads. Finally, we generated 4.5 Gb of sRNA data (Supplemental Table 3).

Genome quality evaluation

Different methods and data were employed to check the completeness of the assembly. Using Bowtie2 [45], we found 87.08%-90.63% of DNA paired end reads (350bp, 500bp and 800bp) could properly mapped to the final assembly genome (Supplemental Table 4, Supplemental Figure 2). We evaluated the completeness of gene regions of our assembly using BUSCO (Benchmarking Universal Single-Copy Orthologs) [46]. Of the 1440 single-copy orthologs presented in the plant lineage, 89.5% was completely identified in the genome (Supplemental Figure 3).

Furthermore, Trinity [47] (r20140413p1) was used to assembled the RNA-seq reads sequenced from the mixed *S. aralocaspica* RNA library into 157,521 unigenes. Then these unigenes aligned to the genome assembly by BLASTN with default parameter. We found 94.5% of the unigenes could be aligned to the genome assembly and 76.3% of the unigenes could be covered in one scaffold with more than 90% of the sequence length. For unigenes longer than 1kb, 99.5% of the unigenes could be aligned to the genome assembly, and 92.8% of the unigenes could be covered in one scaffold with more than 90% of the sequence length (Supplemental Table 5). Taken together, these results suggesting that the *S. aralocaspica* genome assembly is of high quality.

Gene and functional annotation

The genome of *S. aralocaspica* was annotated and distribution of protein-coding genes (PCGs), repeat elements (RE), non-coding genes and other genomic element on the genome were shown (Figure 2). In detail, we used MAKER [48] for PCGs prediction by combining homolog protein data, de novo prediction data, and RNA-seq data. We predicted a total of 29,064 PCGs, with an average transcript length of 4,462 bp, coding sequence size of 1,112 bp, and a mean of 4.76 exons per transcript (Supplemental Tables 6 and 7). Of annotated PCGs, 97.2% were functionally annotated by the InterPro, GO, KEGG, SwissProt or NR databases (Supplemental Figures 4 and 5, Supplemental Table 8) and about 91% were annotated with protein or transcripts support (Supplemental Table 9). The transcription start and termination sites of most of the annotated genes were supported by sequencing reads from CAGE-seq and PAS-seq (Supplemental Figures 6 and 7).

In addition, 1, 651 long noncoding RNA (lncRNA) were predicted followed the method of previous study [49]. 382 transfer RNAs (tRNAs) were predicted using tRNAscan-SE [50]. 21 miRNAs, 88 small nuclear RNAs, and 325 ribosomal RNAs (rRNAs), were identified by using CMscan tool form INFERNAL [51] to search from the Rfam database with option --cut_ga (Supplemental Table 10, Supplemental Figure 8).

Repeat annotation

To annotate the repeat sequence of *S. aralocaspica* genome, a combination of de novo and homology-based approach was employed [52, 53]. For homology-based identification, we used RepeatMasker [54] to search the protein database in Rebase against the *S. aralocaspica* genome to identify TE. The Rebase database (http://www.girinst.org/server/RepBase/index.php) was

used to identify TEs. Parameters of RepeatMasker were set to "-species Viridiplantae -pa 30 -e rmblast". In de novo approach, PILER[55] was used to build the consensus repeat database. PILER software needs PALS, FAMS, PILER to construct the consensus library. The default parameters of PILER were used. Then the predicted consensus TEs were classified by RepeatClassifer implemented in the RepeatModeler package (Version 1.0.11) [56]. We used RepeatMasker to search the TEs within the database constructed by PILER. Finally, we combined the de novo prediction and the homolog prediction of repeat elements according to the coordination in the genome, and detected 173.5Mb repeat elements that constitute 38.41% of the genome (Supplemental Table 11). As observed in other sequenced genomes [57], long terminal repeats (LTRs) [58] in *S. aralocaspica* occupy the majority (48.5%) of repeat sequences (Supplemental Table 12).

Phylogenetic placement of *S. aralocaspica*

To date, *S. aralocaspica* is the first SCC_4 species with a high-quality genome sequence, which we then used as a reference to understand the evolution of genes important for C_4 biology. Previous phylogenetic studies based on the sequence of a few specific genes or intergenic regions reveal that *S. aralocaspica* is more closely related to C_4 *Suaeda* species than to the C_3 ones [14], suggesting the presence of a higher genetic similarity between SCC_4 and C_4. To test this hypothesis, we collected all four reported genome sequences from Chenopodiaceae and one from Amaranthaceae; Chenopodiaceae was merged to Amaranthaceae recently [59]. With the availability of these five genomes, we decided to perform a phylogenetic analysis using single-copy gene families shared by Amaranthaceae species and other 8 C_3, C_4 and CAM

species in eudicot or monocot with sequenced genomes. A gene family survey using OrthoMCL [60] on these 13 plant species yielded 15793 gene families in S. *aralocaspica* containing 22991 predicted genes (77.7% of the total genes identified) (Supplemental Table 13).

Peptide sequences from 143 single-copy orthologous gene clusters in all 13 species generated by OrthoMCL were used to construct phylogenetic relationships and estimate divergence. The peptides of each species were concatenated to a super sequence which were aligned by MUSULE [61] with default options, then the maximum likelihood method was applied to construct a phylogenetic tree by MEGA7 [62]. The resulted phylogenetic tree showed that all five Amaranthaceae species were placed in the same clade, among which *A. hypochondriacus* (C_4) was placed as a sister subclade with other four species (Figure 3). Moreover, *S. aralocaspica* (SCC_4) was the sister clade of other three C_3 species from the Amaranthaceae (Figure 3). These results indicated the SCC_4 would independently evolve in this family. In addition, the phylogenetic tree constructed using single-copy genes supported that Caryophyllales constituted the clade diverged with Rosids in eudicots, which is consistent with previous studies [63, 64].

To identify genes potentially important for SCC_4 biology, we identified 897 gene families that were unique to *S. aralocaspica* when compared with all other 12 plant species (Table. S16). To deciphering their biological functions, Kyoto Encyclopedia of Genes and Genomes (KEGG) enrichment analysis found these gene families to be specifically enriched in the terms including ‘fructose and mannose metabolism’, ‘N-Glycan biosynthesis’, ‘Oxidative phosphorylation’ and ‘carbon fixation in photosynthetic organisms’ (Supplemental Figure 9). These results indicated that *S. aralocaspica* might have evolved some specific genes to adapt SSC_4 photosynthesis.

Assembly of *S. aralocaspica* chloroplast genome

Using the short insert size (350 bp) data, complete (circular with no gaps) chloroplast (cp) genomes of *S. aralocaspica* was assembled to be 146,654 in length with NOVOPlasty [65]. The Rubisco-bis-phosphate oxygenase (RuBP) subunit of Chenopodium quinoa (KY419706) was selected as a seed sequence. Initial gene annotation of the genome was performed with GeSeq [66]. The circular cp genome maps were drawn using the OrganellarGenome DRAW tool [67], with subsequent manual editing (Figure 4).

Conclusion

Using the Illumina and Pacbio platforms, we successfully assembled the genome of *S. aralocaspica*, which was the first genome sequence of the SCC_4 plant. The final genome assembly is 452 Mb and consisted of 4033 scaffolds with a scaffolds N50 length of 1.83 Mb. We annotated xxx protein-coding genes and noncoding genes including 1, 651 lncRNA, 21 miRNAs, 382 tRNAs, 88 small nuclear RNAs, 325 rRNAs. The phylogenetic tree placed SCC_4 in a clade between C_4 and C_3 , supporting the hypothesis that SCC_4 is an C_3-C_4 intermediate [35, 68] and independently evolved from the C_3 ancestors. The available genome assembly, together with transcriptomic data of *S. aralocaspica*, provide a valuable resource for investigating C_4 evolution and mechanisms. We anticipate that future studies of *S. aralocaspica*

will greatly facilitate the process of engineering higher photosynthetic efficiency and saline tolerant crops, especially in C_3 species including rice.

Availability of supporting data

Raw sequencing data are deposited in the Sequence Read Archive with accession number SRP128359. The NCBI Bioproject accession is PRJNA428881.

Additional files

Supplemental Figure 1. K-mer distribution of sequencing reads.

Supplemental Figure 2. Size distribution of inserts in sequenced paired-end DNA reads.

Supplemental Figure 3. Integrity comparison of genome assemblies of *S. aralocaspica* with BUSCO. For S. aralocaspica, assemblies in each steps were analyzed respectively.

Supplemental Figure 4. Annotated genes supported by different manners.

Supplemental Figure 5. Gene ontology distribution of *S. aralocaspica* protein coding genes.

Supplemental Figure 6. Transcription start site (TSS) annotation with Cage-seq.

Supplemental Figure 7. Transcription terminal site (TTS) annotation with Pas-seq.

Supplemental Figure 8. Non-coding RNAs classification in S. aralocaspica.

Supplemental Figure 9. Functional enrichment of *S. aralocaspica*-specific gene families among 13 species.

Supplemental Table 1. Summary of sequencing data obtained for genome assembly.

Supplemental Table 2. The assembly statistics of the *S. aralocaspica* genome.

Supplemental Table 3. Information of different types of RNA libraries.

Supplemental Table 4. Mapping efficiency of short insert library reads

Supplemental Table 5. Assessment of sequence coverage of *S. aralocaspica* genome assembly using unigenes.

Supplemental Table 6. Gene prediction in the *S. aralocaspica* genome.

Supplemental Table 7. Comparison of the gene structure among *S. aralocaspica* and some other species

Supplemental Table 8. Summary of *S. aralocaspica* gene annotation based on homology or functional classification.

Supplemental Table 9. Number of *S. aralocaspica* genes with protein or unigene support.

Supplemental Table 10. Noncoding RNA genes in the *S. aralocaspica* genome.

Supplemental Table 11. Repeat elements in the *S. aralocaspica* genome. Repeat elements were identified by different methods and then combined into a final repeat set.

Supplemental Table 12. Repeat elements in *S. aralocaspica* genomes.

Supplemental Table 13. Gene families clustered by OrthoMCL in 13 species.

Abbreviations

SCC_4: single-cell C_4 photosynthesis; CAM: crassulacean acid metabolism; lncRNA: long non-coding RNAs; PCG: protein-coding gene;

Competing interests

The authors declare that they have no competing interests.

Funding

This research was supported by the National Natural Science Foundation of China (31770451), the National Key Research and Development Program (2016YFC0501400), Self-Determined

Project of State Key Laboratory of Desert and Oasis Ecology (Y371162) and ABLife (ABL2014-02028).

Author contributions

C.T., L.W., Yi Z. and S.M. initiated the project and designed the study. L.W., H.W., L.J., Z.Z. and K.Z. prepared experimental materials and performed experiments for data collection. G.M., C.C., Yu Z., H.W., L.J. and K.Z. assembled the genome, analyzed the data and generated the graphs. Yi Z., W.Q., C.T., L.W. and X.W. wrote the manuscript.

References

1. Walker BJ, VanLoocke A, Bernacchi CJ and Ort DR. The Costs of Photorespiration to Food Production Now and in the Future. Annual Review of Plant Biology. 2016;67 1:107-29. doi:10.1146/annurev-arplant-043015-111709.

2. Yamori W, Hikosaka K and Way DA. Temperature response of photosynthesis in C_3, C_4, and CAM plants: temperature acclimation and temperature adaptation. Photosynthesis Res. 2014;119 1-2:101-17. doi:10.1007/s11120-013-9874-6.

3. Hibberd JM, Sheehy JE and Langdale JA. Using C_4 photosynthesis to increase the yield of rice-rationale and feasibility. Curr Opin Plant Biol. 2008;11 2:228-31. doi:https://doi.org/10.1016/j.pbi.2007.11.002.

4. von Caemmerer S, Quick WP and Furbank RT. The Development of C4 Rice: Current Progress and Future Challenges. Science. 2012;336 6089:1671-2. doi:10.1126/science.1220177.

5. Gu J-F, Qiu M and Yang J-C. Enhanced tolerance to drought in transgenic rice plants overexpressing C4 photosynthesis enzymes. The Crop Journal. 2013;1 2:105-14.

doi:https://doi.org/10.1016/j.cj.2013.10.002.

6. Betti M, Bauwe H, Busch FA, Fernie AR, Keech O, Levey M, et al. Manipulating photorespiration to increase plant productivity: recent advances and perspectives for crop improvement. Journal of Experimental Botany. 2016;67 10:2977-88. doi:10.1093/jxb/erw076.

7. Akhani H, Trimborn P and Ziegler H. Photosynthetic pathways in Chenopodiaceae from Africa, Asia and Europe with their ecological, phytogeographical and taxonomical importance. Plant Syst Evol. 1997;206 1:187-221. doi:10.1007/bf00987948.

8. Sage RF, Li M and Monson RK. The taxonomic distribution of C_4 photosynthesis. In: Sage RF and Monson RK, editors. C_4 plant biology. San Diego, California, USA: Academic Press; 1999. p. 551-84.

9. Jacobs SWL. Review of leaf anatomy and ultrastructure in the Chenopodiaceae (Caryophyllales). J Torrey Bot Soc. 2001;128 3:236-53.

10. Voznesenskaya EV, Franceschi VR, Kiirats O, Freitag H and Edwards GE. Kranz anatomy is not essential for terrestrial C4 plant photosynthesis. Nature. 2001;414 6863:543-6. doi:10.1038/35107073.

11. Voznesenskaya EV, Franceschi VR, Kiirats O, Artyusheva EG, Freitag H and Edwards GE. Proof of C4 photosynthesis without Kranz anatomy in Bienertia cycloptera (Chenopodiaceae). The Plant Journal. 2002;31 5:649-62. doi:10.1046/j.1365-313X.2002.01385.x.

12. Akhani H, Barroca J, Koteeva N, Voznesenskaya E, Franceschi V, Edwards G, et al. Bienertia sinuspersici (Chenopodiaceae): A New Species from Southwest Asia and

Discovery of a Third Terrestrial C4 Plant Without Kranz Anatomy. Systematic Botany. 2005;30 2:290-301. doi:10.1600/0363644054223684.

13. Akhani H, Chatrenoor T, Dehghani M, Khoshravesh R, Mahdavi P and Matinzadeh Z. A new species of *Bienertia* (Chenopodiaceae) from Iranian salt deserts: A third species of the genus and discovery of a fourth terrestrial C_4 plant without Kranz anatomy. Plant Biosystems. 2012;146 3:550-9. doi:10.1080/11263504.2012.662921.

14. Schütze P, Freitag H and Weising K. An integrated molecular and morphological study of the subfamily Suaedoideae Ulbr. (Chenopodiaceae). Plant Syst Evol. 2003;239 3:257-86. doi:10.1007/s00606-003-0013-2.

15. Voznesenskaya EV, Edwards GE, Kiirats O, Artyusheva EG and Franceschi VR. Development of biochemical specialization and organelle partitioning in the single-cell C_4 system in leaves of *Borszczowia aralocaspica* (Chenopodiaceae). Am J Bot. 2003;90 12:1669-80. doi:10.3732/ajb.90.12.1669.

16. Voznesenskaya EV, Koteyeva NK, Chuong SD, Akhani H, Edwards GE and Franceschi VR. Differentiation of cellular and biochemical features of the single-cell C_4 syndrome during leaf development in *Bienertia cycloptera* (Chenopodiaceae). Am J Bot. 2005;92 11:1784-95. doi:10.3732/ajb.92.11.1784.

17. Offermann S, Okita TW and Edwards GE. Resolving the compartmentation and function of C_4 photosynthesis in the single-cell C_4 species *Bienertia sinuspersici*. Plant Physiol. 2011;155 4:1612-28. doi:10.1104/pp.110.170381.

18. Offermann S, Friso G, Doroshenk KA, Sun Q, Sharpe RM, Okita TW, et al. Developmental and subcellular organization of single-cell C_4 photosynthesis in

Bienertia sinuspersici determined by large-scale proteomics and cDNA assembly from 454 DNA sequencing. Journal of proteome research. 2015;14 5:2090-108. doi:10.1021/pr5011907.

19. Wimmer D, Bohnhorst P, Shekhar V, Hwang I and Offermann S. Transit peptide elements mediate selective protein targeting to two different types of chloroplasts in the single-cell C_4 species *Bienertia sinuspersici*. Sci Rep. 2017;7:41187. doi:10.1038/srep41187.

20. Jurić I, González-Pérez V, Hibberd JM, Edwards G and Burroughs NJ. Size matters for single-cell C4 photosynthesis in Bienertia. Journal of Experimental Botany. 2017;68 2:255-67. doi:10.1093/jxb/erw374.

21. Stutz SS, Edwards GE and Cousins AB. Single-cell C_4 photosynthesis: efficiency and acclimation of *Bienertia sinuspersici* to growth under low light. The New phytologist. 2014;202 1:220-32. doi:10.1111/nph.12648.

22. Lung SC, Yanagisawa M and Chuong SD. Protoplast isolation and transient gene expression in the single-cell C_4 species, *Bienertia sinuspersici*. Plant Cell Rep. 2011;30 4:473-84. doi:10.1007/s00299-010-0953-2.

23. Leisner CP, Cousins AB, Offermann S, Okita TW and Edwards GE. The effects of salinity on photosynthesis and growth of the single-cell C_4 species *Bienertia sinuspersici* (Chenopodiaceae). Photosynthesis Res. 2010;106 3:201-14. doi:10.1007/s11120-010-9595-z.

24. Uzilday B, Ozgur R, Yalcinkaya T, Turkan I and Sekmen AH. Changes in redox regulation during transition from C_3 to single cell C_4 photosynthesis in Bienertia

sinuspersici. J Plant Physiol. 2017;220:1-10. doi:10.1016/j.jplph.2017.10.006.

25. Koteyeva NK, Voznesenskaya EV, Berry JO, Cousins AB and Edwards GE. The unique structural and biochemical development of single cell C_4 photosynthesis along longitudinal leaf gradients in *Bienertia sinuspersici* and *Suaeda aralocaspica* (Chenopodiaceae). J Exp Bot. 2016;67 9:2587-601. doi:10.1093/jxb/erw082.

26. Rosnow J, Yerramsetty P, Berry JO, Okita TW and Edwards GE. Exploring mechanisms linked to differentiation and function of dimorphic chloroplasts in the single cell C4 species *Bienertia sinuspersici*. BMC Plant Biol. 2014;14:34. doi:10.1186/1471-2229-14-34.

27. Park J, Knoblauch M, Okita TW and Edwards GE. Structural changes in the vacuole and cytoskeleton are key to development of the two cytoplasmic domains supporting single-cell C(4) photosynthesis in Bienertia sinuspersici. Planta. 2009;229 2:369-82. doi:10.1007/s00425-008-0836-8.

28. Lara MV, Offermann S, Smith M, Okita TW, Andreo CS and Edwards GE. Leaf development in the single-cell C_4 system in *Bienertia sinuspersici*: expression of genes and peptide levels for C_4 metabolism in relation to chlorenchyma structure under different light conditions. Plant Physiol. 2008;148 1:593-610. doi:10.1104/pp.108.124008.

29. Chuong SD, Franceschi VR and Edwards GE. The cytoskeleton maintains organelle partitioning required for single-cell C4 photosynthesis in Chenopodiaceae species. Plant Cell. 2006;18 9:2207-23. doi:10.1105/tpc.105.036186.

30. Wang L, Huang Z, Baskin CC, Baskin JM and Dong M. Germination of dimorphic seeds

of the desert annual halophyte *Suaeda aralocaspica* (Chenopodiaceae), a C_4 plant without Kranz anatomy. Ann Bot. 2008;102 5:757-69. doi:10.1093/aob/mcn158.

31. Wang L, Baskin JM, Baskin CC, Cornelissen JH, Dong M and Huang Z. Seed dimorphism, nutrients and salinity differentially affect seed traits of the desert halophyte *Suaeda aralocaspica* via multiple maternal effects. BMC Plant Biol. 2012;12:170. doi:10.1186/1471-2229-12-170.

32. Cao J, Lv XY, Chen L, Xing JJ and Lan HY. Effects of salinity on the growth, physiology and relevant gene expression of an annual halophyte grown from heteromorphic seeds. AoB Plants. 2015;7:plv112. doi:10.1093/aobpla/plv112.

33. Wang HL, Tian CY and Wang L. Germination of dimorphic seeds of *Suaeda aralocaspica* in response to light and salinity conditions during and after cold stratification. PeerJ. 2017;5:e3671. doi:10.7717/peerj.3671.

34. Wang L, Wang HL, Yin L and Tian CY. Transcriptome assembly in *Suaeda aralocaspica* to reveal the distinct temporal gene/miRNA alterations between the dimorphic seeds during germination. BMC Genomics. 2017;18:806. doi:10.1186/s12864-017-4209-1.

35. Edwards GE and Voznesenskaya EV. C_4 photosynthesis: Kranz forms and single-cell C_4 in terrestrial plants. In: Raghavendra AS and Sage RF, editors. C_4 photosynthesis and related CO_2 concentrating mechanisms. Dordrecht: Springer Netherlands; 2011. p. 29-61.

36. Sharpe RM and Offermann S. One decade after the discovery of single-cell C_4 species in terrestrial plants: what did we learn about the minimal requirements of C_4

photosynthesis? Photosynthesis Res. 2014;119 1-2:169-80. doi:10.1007/s11120-013-9810-9.

37. Badouin H, Gouzy J, Grassa CJ, Murat F, Staton SE, Cottret L, et al. The sunflower genome provides insights into oil metabolism, flowering and Asterid evolution. Nature. 2017;546 7656:148-52. doi:10.1038/nature22380.

38. Jarvis DE, Ho YS, Lightfoot DJ, Schmöckel SM, Li B, Borm TJA, et al. The genome of Chenopodium quinoa. Nature. 2017;542:307. doi:10.1038/nature21370 https://www.nature.com/articles/nature21370#supplementary-information.

39. Zhang GQ, Liu KW, Li Z, Lohaus R, Hsiao YY, Niu SC, et al. The Apostasia genome and the evolution of orchids. Nature. 2017;549 7672:379-83. doi:10.1038/nature23897.

40. Zhao G, Zou C, Li K, Wang K, Li T, Gao L, et al. The *Aegilops tauschii* genome reveals multiple impacts of transposons. Nature Plants. 2017; doi:10.1038/s41477-017-0067-8.

41. Liu B, Shi Y, Yuan J, Hu X, Zhang H, Li N, et al. Estimation of genomic characteristics by analyzing k-mer frequency in de novo genome projects. arXiv: Genomics. 2013.

42. Li R, Zhu H, Ruan J, Qian W, Fang X, Shi Z, et al. De novo assembly of human genomes with massively parallel short read sequencing. Genome Res. 2010;20 2:265-72. doi:10.1101/gr.097261.109.

43. The-Tomato-Genome-Consortium. The tomato genome sequence provides insights into fleshy fruit evolution. Nature. 2012;485:635. doi:10.1038/nature11119 https://www.nature.com/articles/nature11119#supplementary-information.

44. English AC, Richards S, Han Y, Wang M, Vee V, Qu J, et al. Mind the gap: upgrading

genomes with Pacific Biosciences RS long-read sequencing technology. PLoS One. 2012;7 11:e47768. doi:10.1371/journal.pone.0047768.

45. Li H and Durbin R. Fast and accurate short read alignment with Burrows-Wheeler transform. Bioinformatics. 2009;25 14:1754-60. doi:10.1093/bioinformatics/btp324.

46. Simao FA, Waterhouse RM, Ioannidis P, Kriventseva EV and Zdobnov EM. BUSCO: assessing genome assembly and annotation completeness with single-copy orthologs. Bioinformatics. 2015;31 19:3210-2. doi:10.1093/bioinformatics/btv351.

47. Grabherr MG, Haas BJ, Yassour M, Levin JZ, Thompson DA, Amit I, et al. Full-length transcriptome assembly from RNA-Seq data without a reference genome. Nat Biotechnol. 2011;29 7:644-52. doi:10.1038/nbt.1883.

48. Cantarel BL, Korf I, Robb SM, Parra G, Ross E, Moore B, et al. MAKER: an easy-to-use annotation pipeline designed for emerging model organism genomes. Genome Res. 2008;18 1:188-96. doi:10.1101/gr.6743907.

49. Cabili MN, Trapnell C, Goff L, Koziol M, Tazon-Vega B, Regev A, et al. Integrative annotation of human large intergenic noncoding RNAs reveals global properties and specific subclasses. Genes Dev. 2011;25 18:1915-27. doi:10.1101/gad.17446611.

50. Lowe TM and Eddy SR. tRNAscan-SE: a program for improved detection of transfer RNA genes in genomic sequence. Nucleic Acids Res. 1997;25 5:955-64.

51. Nawrocki EP, Kolbe DL and Eddy SR. Infernal 1.0: inference of RNA alignments. Bioinformatics. 2009;25 10:1335-7. doi:10.1093/bioinformatics/btp157.

52. Iorizzo M, Senalik DA, Grzebelus D, Bowman M, Cavagnaro PF, Matvienko M, et al. De novo assembly and characterization of the carrot transcriptome reveals novel genes,

new markers, and genetic diversity. BMC Genomics. 2011;12:389. doi:10.1186/1471-2164-12-389.

53. Wang L, Yu S, Tong C, Zhao Y, Liu Y, Song C, et al. Genome sequencing of the high oil crop sesame provides insight into oil biosynthesis. Genome Biology. 2014;15 2:R39. doi:10.1186/gb-2014-15-2-r39.

54. Tarailo-Graovac M and Chen N. Using RepeatMasker to identify repetitive elements in genomic sequences. Current protocols in bioinformatics. 2009;Chapter 4:Unit 4.10. doi:10.1002/0471250953.bi0410s25.

55. Edgar RC and Myers EW. PILER: identification and classification of genomic repeats. Bioinformatics. 2005;21 Suppl 1:i152-i8. doi:10.1093/bioinformatics/bti1003.

56. Rao SK, Fukayama H, Reiskind JB, Miyao M and Bowes G. Identification of C4 responsive genes in the facultative C4 plant *Hydrilla verticillata*. Photosynthesis Res. 2006;88 2:173-83. doi:10.1007/s11120-006-9049-9.

57. Vlasova A, Capella-Gutierrez S, Rendon-Anaya M, Hernandez-Onate M, Minoche AE, Erb I, et al. Genome and transcriptome analysis of the Mesoamerican common bean and the role of gene duplications in establishing tissue and temporal specialization of genes. Genome Biology. 2016;17:32. doi:10.1186/s13059-016-0883-6.

58. Wicker T, Sabot F, Hua-Van A, Bennetzen JL, Capy P, Chalhoub B, et al. A unified classification system for eukaryotic transposable elements. Nature reviews Genetics. 2007;8 12:973-82. doi:10.1038/nrg2165.

59. Angiosperm-Phylogeny-Group. An update of the Angiosperm Phylogeny Group classification for the orders and families of flowering plants: APG II. Bot J Linn Soc.

2003;141 4:399-436. doi:10.1046/j.1095-8339.2003.t01-1-00158.x.

60. Li L, Stoeckert CJ, Jr. and Roos DS. OrthoMCL: identification of ortholog groups for eukaryotic genomes. Genome Res. 2003;13 9:2178-89. doi:10.1101/gr.1224503.

61. Edgar RC. MUSCLE: multiple sequence alignment with high accuracy and high throughput. Nucleic Acids Res. 2004;32 5:1792-7. doi:10.1093/nar/gkh340.

62. Kumar S, Stecher G and Tamura K. MEGA7: molecular evolutionary genetics analysis version 7.0 for bigger datasets. Mol Biol Evol. 2016;33 7:1870-4. doi:10.1093/molbev/msw054.

63. Xu C, Jiao C, Sun H, Cai X, Wang X, Ge C, et al. Draft genome of spinach and transcriptome diversity of 120 Spinacia accessions. Nature Communications. 2017;8:15275. doi:10.1038/ncomms15275 https://www.nature.com/articles/ncomms15275#supplementary-information.

64. Dohm JC, Minoche AE, Holtgrawe D, Capella-Gutierrez S, Zakrzewski F, Tafer H, et al. The genome of the recently domesticated crop plant sugar beet (Beta vulgaris). Nature. 2014;505 7484:546-9. doi:10.1038/nature12817.

65. Dierckxsens N, Mardulyn P and Smits G. NOVOPlasty: de novo assembly of organelle genomes from whole genome data. Nucleic Acids Res. 2017;45 4:e18. doi:10.1093/nar/gkw955.

66. Tillich M, Lehwark P, Pellizzer T, Ulbricht-Jones ES, Fischer A, Bock R, et al. GeSeq - versatile and accurate annotation of organelle genomes. Nucleic Acids Res. 2017;45 W1:W6-W11. doi:10.1093/nar/gkx391.

67. Lohse M, Drechsel O, Kahlau S and Bock R. OrganellarGenomeDRAW--a suite of tools

for generating physical maps of plastid and mitochondrial genomes and visualizing expression data sets. Nucleic Acids Res. 2013;41 Web Server issue:W575-81. doi:10.1093/nar/gkt289.

68. Kapralov MV, Akhani H, Voznesenskaya EV, Edwards G, Franceschi V and Roalson EH. Phylogenetic Relationships in the Salicornioideae / Suaedoideae / Salsoloideae s.l. (Chenopodiaceae) Clade and a Clarification of the Phylogenetic Position of Bienertia and Alexandra Using Multiple DNA Sequence Datasets. Systematic Botany. 2006;31 3:571-85. doi:10.1043/06-01.1.

69. Tamura K, Battistuzzi FU, Billing-Ross P, Murillo O, Filipski A and Kumar S. Estimating divergence times in large molecular phylogenies. Proc Natl Acad Sci U S A. 2012;109 47:19333-8. doi:10.1073/pnas.1213199109.

70. Jones DT, Taylor WR and Thornton JM. The rapid generation of mutation data matrices from protein sequences. Bioinformatics. 1992;8 3:275-82.

Figure legend

Figure 1: Example of *S. aralocaspica.*

Figure 2: Distribution of genomic features in the *S. aralocaspica* genome. Track **a**, a virtual chromosome merged by all of the 4033 contigs. One scale label indicates 2 Mb. The blue and green bands indicated different length contigs arranged. Track **b**, PAS-seq reads binding site density on the genome length per 500 kb ranged from 0 to 0.14. Track **c**, CAGE-seq reads binding site density on the genome length per 500 kb ranged from 0 to 0.19. Track **d**, lncRNA predicted number on the genome length per 500 kb ranged from 0 to 8. Track **e**, GC content; the ratio of GC sites per 500 kb ranged from 0.05 to 0.31. Track **f**, repeat elements density (TEs); the frequency of repeats sites percent in genome regions per 500kb ranged from 0 to 0.69. Track **g**, gene density (mRNA); the frequency of gene numbers within genome regions per 1Mb ranged from 0 to 135. Track **h**, marks the genomic position of photosynthesis genes. Track **i**, indicates self-collinearity of *S. aralocaspica*, co-linear segments with at least 10 anchor pairs are shown.

Figure 3: The phylogenetic relationship of the *S. aralocaspica* with other C_3/C_4 plants. A time-tree inferred using the Reltime method [69] and the JTT matrix-based model[70]. The estimated log likelihood value is -52002.30. The analysis involved 15 amino acid sequences which were combined by single copy genes from 15 plant genomes. All positions containing gaps and missing data were eliminated. There were a total of 3730 positions in the final dataset. Evolutionary analyses were conducted in MEGA7.

Figure1 Click here to access/download;Figure;Figure.1.pdf

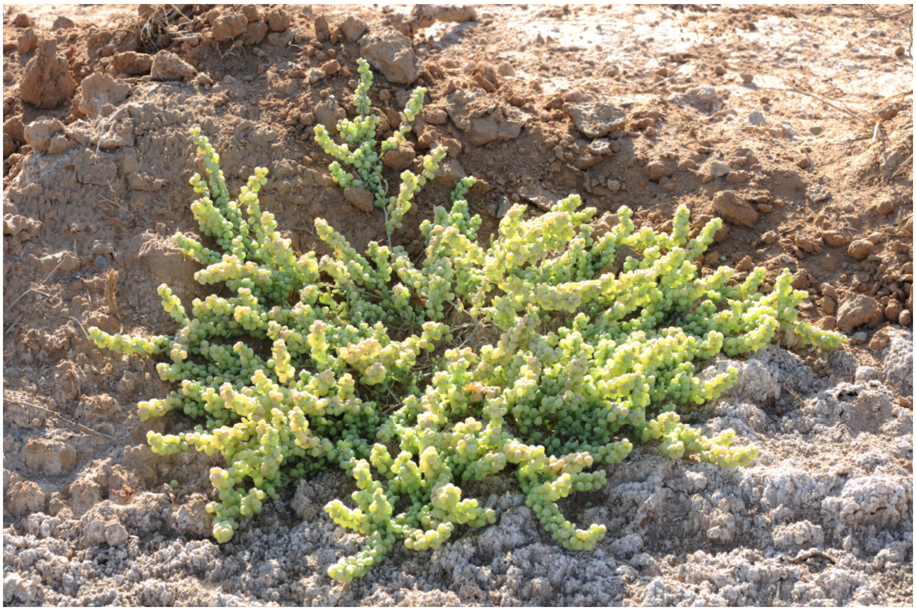

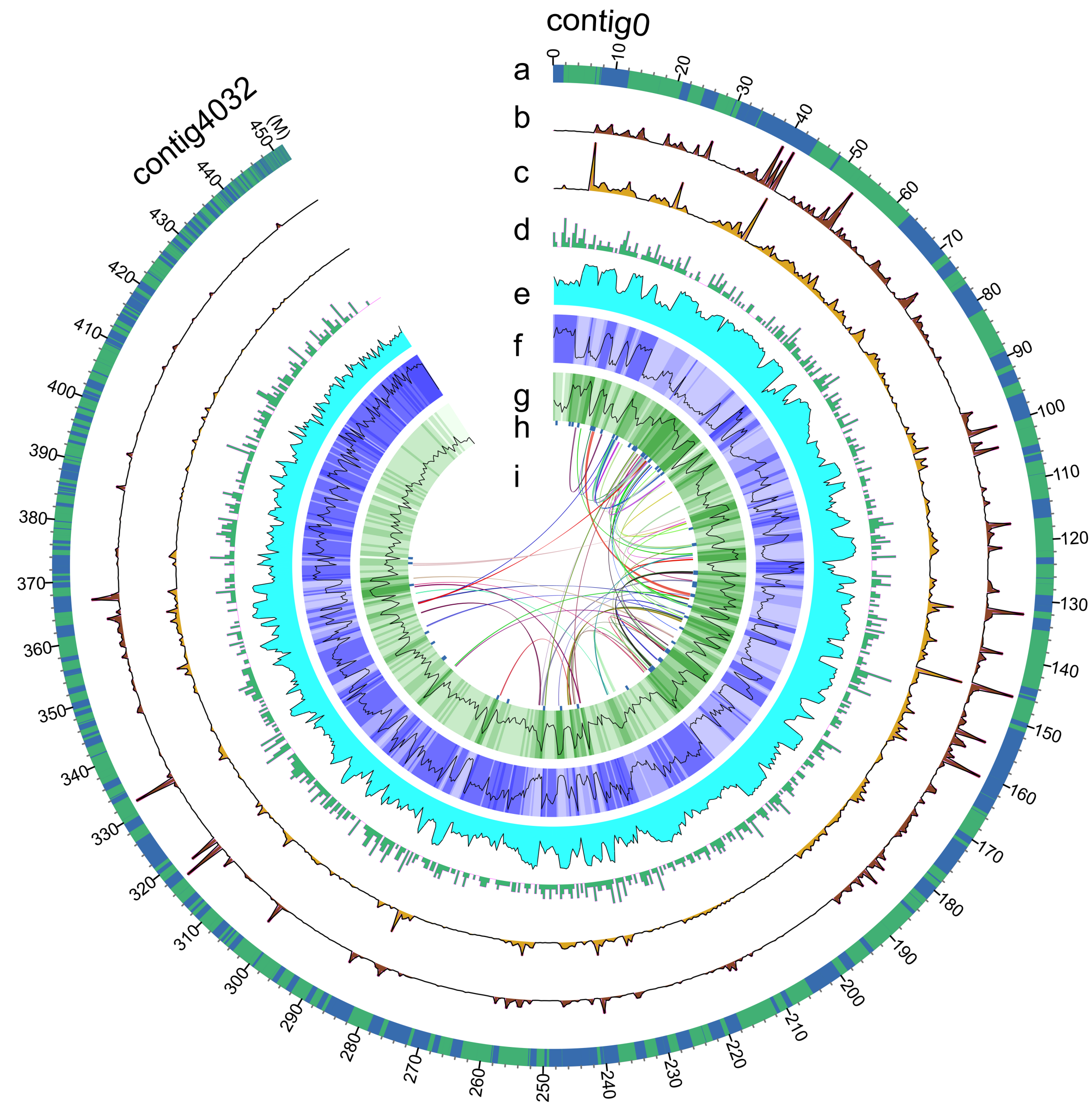
contig0
contig4032
(M)
a
b
c
d
e
f
g
h
i

Figure3

Click here to access/download;Figure;Figure.3.pdf

a

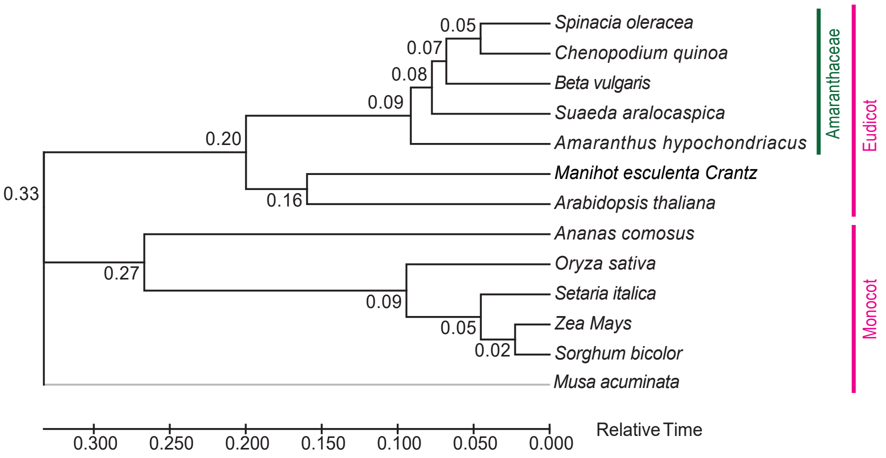

Figure4

Click here to access/download;Figure;Figure.4.pdf

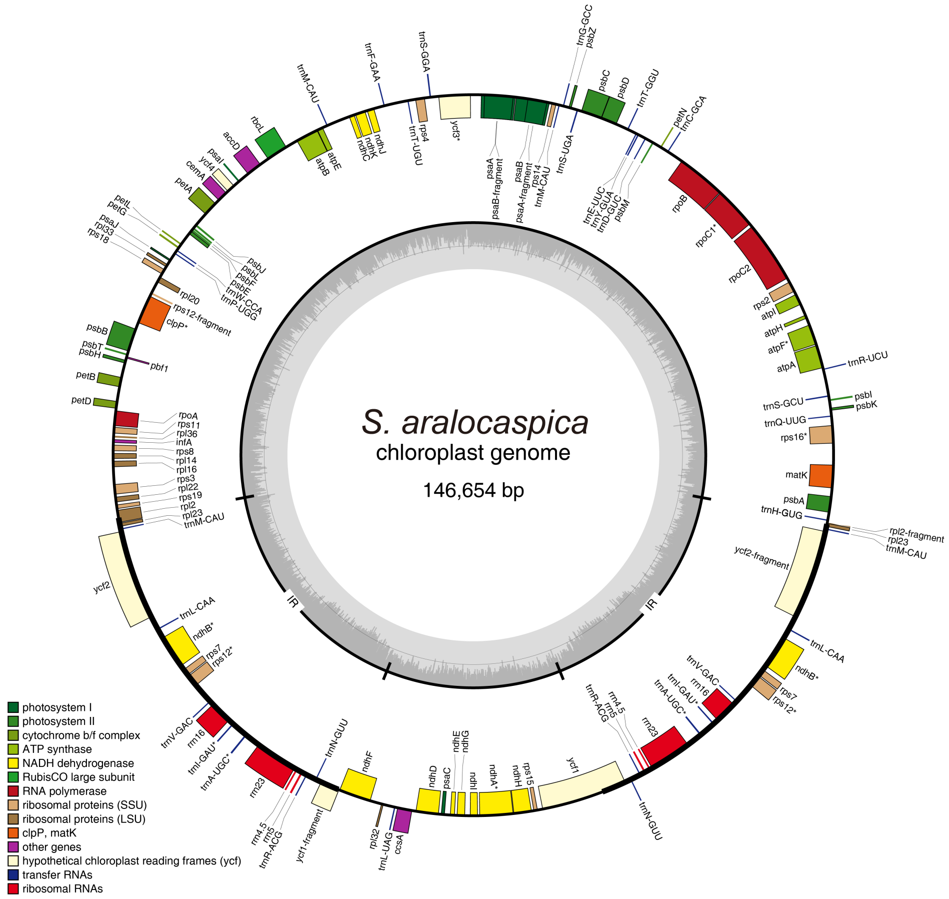

Supplemental figures and tables

Click here to access/download
Supplementary Material
Supplemental-Information-for-gigascience.docx
