# Supplementary material for: A draft genome assembly of halophyte Suaeda aralocaspica, a plant that performs C4 photosynthesis within individual cells
Source: Gigascience. 2019 Sep 12;8(9):giz116. doi: 10.1093/gigascience/giz116 (PMC6741815; doi:10.1093/gigascience/giz116)
Supplement: giz116_GIGA-D-19-00024_Revision_1 [file giz116_giga-d-19-00024_revision_1.pdf]

GigaScience

A draft genome assembly of halophyte Suaeda aralocaspica, a plant that performs C_4 photosynthesis within individual cells

--Manuscript Draft--

Manuscript Number:	GIGA-D-19-00024R1
Full Title:	A draft genome assembly of halophyte Suaeda aralocaspica, a plant that performs C_4 photosynthesis within individual cells
Article Type:	Data Note
Funding Information:	the Key Research and Development Program of Xinjiang province (2018B01006-4) — Dr. Lei Wang<br>National Natural Science Foundation of China (31770451) — Dr. Lei Wang<br>National Key Research and Development Program (2016YFC0501400) — Prof. Changyan Tian<br>ABLife (ABL2014-02028) — Dr. Yi Zhang
Abstract:	Background: The halophyte Suaeda aralocaspica performs complete C_4 photosynthesis within individual cells (SCC_4), which is distinct from typical C_4 plants that require the collaboration of two types of photosynthetic cells. However, despite SCC_4 plants having features that are valuable in engineering higher photosynthetic efficiencies in C_3 species, including rice, there are no reported sequenced SCC_4 plant genomes, which limits our understanding of the mechanisms involved in, and evolution of, SCC_4 photosynthesis.<br>Findings: Using Illumina and Pacbio platforms, we generated ~202 Gb of clean genomic DNA sequences having a 433-fold coverage based on the 467 Mb estimated genome size of S. aralocaspica. The final genome assembly was 452 Mb, consisting of 4,033 scaffolds, with a scaffold N50 length of 1.83 Mb. We annotated 29,604 protein-coding genes using Evidence Modeler based on the gene information from ab initio predictions, homology levels with known genes, and RNA sequencing-based transcriptome evidence. We also annotated noncoding genes, including 1,651 long noncoding RNAs, 21 microRNAs, 382 transfer RNAs, 88 small nuclear RNAs, and 325 ribosomal RNAs. A complete (circular with no gaps) chloroplast genome of S. aralocaspica was also assembled to be 146,654 bp in length.<br>Conclusions: We have presented the genome sequence of S. aralocaspica, a SCC_4 plant. Knowledge of the genome of S. aralocaspica should increase our understanding of SCC_4 photosynthesis' evolution and contribute to the engineering of C_4 photosynthesis into economically important C_3 crops.
Corresponding Author:	Yi Zhang, Ph.D.<br><br>CHINA
Corresponding Author Secondary Information:	
Corresponding Author's Institution:	
Corresponding Author's Secondary Institution:	
First Author:	Lei Wang
First Author Secondary Information:	
Order of Authors:	Lei Wang<br>Ganglong Ma<br>Hongling Wang

Powered by Editorial Manager® and ProduXion Manager® from Aries Systems Corporation

	Chao Cheng
	Shuyong Mu
	Weili Quan
	Li Jiang
	Zhenyong Zhao
	Yu Zhang
	Ke Zhang
	Xuelian Wang
	Changyan Tian
	Yi Zhang, Ph.D.
**Order of Authors Secondary Information:**	

Response to Reviewers:

July 12, 2019

Dear Editor,

We are pleased to submit the revised manuscript entitled “A draft genome assembly of halophyte Suaeda aralocaspica, a plant that performs C_4 photosynthesis within individual cells”. Attached please find our detailed responses to your comments and those raised by both reviewers. The manuscript has been revised accordingly. All authors greatly appreciate the critical comments which we have used to guide our revisions to improve the manuscript.

We believe the quality and clarity of the manuscript has been improved significantly, which we hope is now in a form suitable for publication in GigaScience as a Data Note.

Yours sincerely,
Dr. Yi Zhang
Center for Genome Analysis, ABLife Inc., Wuhan, Hubei 430075, China
Email: yizhang@ablife.cc

Comments from the editor and responses:

As you will see the reviewers felt significant additional work is required to improve the writing, methodological detail and validation/analysis. We are willing to continue to consider the manuscript if you are able to address these points, but we will only send it back to the reviewers if you are able to carry out this additional work thoroughly. This includes getting a native English speaker or commercial copy editor to improve the language. And improve the methodological detail, for example including detailed protocols via protocols.io (these can be reused or adapted from protocols already submitted https://www.protocols.io/groups/gigascience-journal).

Response: According the your kind advice, we have sent our revised manuscript to a commercial copy editor in International Science Editing (http://www.internationalscienceediting.com) to improve the language. In addition, we have updated the methodological details in manuscript and also uploaded the detailed commands <detailed_commands_for_assembly_and_annotation.docx> and house-scripts used in genome assembly and annotation to GigaDB database.

Responses to the reviewers’ comments are detailed below.

Comments from the reviewers and responses:

Reviewer #1: Wang et al. generate a draft genome of Suaeda aralocaspica, a halophyte with single cell C_4 photosynthesis. They use a primarily Illumina based

Powered by Editorial Manager® and ProduXion Manager® from Aries Systems Corporation

approach with gap filling using low coverage PacBio data. This resource will be useful for the comparative genomics and C_4 research communities. I have some concerns I feel should be addressed before this manuscript is suitable for publication.

Major:
Were the gaps that were filled by PacBio data polished? Raw PacBio reads have an error rate of ~5-10% and these patched regions would be full of errors if there was no polishing. The Raw PacBio reads could be polished against themselves prior to gap filling with PBJelly or the regions could be polished with Illumina data using a program such as Pilon.

Response: We appreciate the reviewer's comment. Sorry that we missed this important information in the last version of the manuscript. The details have been added to the revised manuscript (lines 121-124). In this study, raw PacBio reads were polished with Illumina data using Proovread (https://github.com/BioInf-Wuerzburg/proovread) to ensure the high accuracy of the PacBio reads used for genome assembly.

Minor:
Figure 2 is not particularly informative and slightly misleading. Circos plots of chromosomes/pseudomolecules can be useful for looking at gene and repeat density as well as macrosynteny (for polyploids and for detecting whole genome duplication events). Here, the 4,033 contigs are stitched together into one continuous block and various features are highlighted. Because most scaffolds are relatively small, rolling windows of features seem meaningless (particularly track i).

Response: We concur with the reviewer's comment, and decided to remove this figure from the revised manuscript accordingly.

Line 205: more details on what MAKER parameters were used are needed, particularly which gene prediction programs were used (i.e. Snap, Augustus, etc). The BUSCO completeness of the annotation should also be provided.

Response: Thanks for the reviewer's comment, and detailed MAKER parameters were added to revised manuscript (lines 224-231) accordingly. De novo predictions were processed by AUGUSTUS (v3.2.1). The BUSCO completeness of the annotation was provided at Lines 212-214.

I would caution the authors against using 'first' in the title. To my knowledge, this is the first plant with single cell C_4 to be sequenced, but that might not be the case when this paper is published.

Response: Thanks for this specific comment. The title was changed to: "A draft genome assembly of halophyte Suaeda aralocaspica, a plant that performs C_4 photosynthesis within individual cells".

Reviewer #2: Gigascience 4.2019
Here, the authors present the wet lab work, some bioinformatics information and short analysis of the genome of S. aralocaspica, the first SCC_4 genome available. This is a short technical / data report of the public availability of a genome. I appreciate the simplicity this type of article; however, I have a number of scientific and data presentation comments. These are detailed below.

Genome analysis:
The genome analyses (phylogenetics, gene families) leave much to be desired. They are not well described, interpretation is nearly completely lacking and the scope of the analyses are very narrow. I understand that this is a data note; however, if such analyses are going to be conducted, they should be done in a rigorous manner, with sufficient replication, and include a complete methodological description and scientific interpretation.
What does 'SCC_4 would independently evolve in this family' mean? Is it that the trees indicate that SCC_4 is a derived trait in the family? If so, to make this claim, you would

need to analyze gene / species trees with many more species. It could easily be that the observed pattern is simply due to very small sample size.
Same goes for the private-gene family analysis. If the authors want to actually perform this analysis they need to add many more genomes. This is in part to capture the evolution of these families, and in part because sequence alignments of highly diverged and sparsely sampled sequences are highly biased and inaccurate.

Response: Many thanks for your critical comments. We have re-performed the comparative genome analysis, using all annotated protein sequences from 18 sequenced plant genomes including eight C_3 species, eight C_4 species and two CAM species. The analysis We also rewrote the result presentation under the subtitle of “Phylogenetic placement of S. aralocaspica” accordingly. In fact, we tried to use more species, however, which led to no single copy gene for analysis. In the revised version, we also added a couple of interpretation and expanded the scope of the analyses.

Text:
There are a lot of proofreading and grammar errors. The most obvious is line 282: 'We annotated xxx protein-coding genes and noncoding genes' … This type of error is very concerning and makes me skeptical that the authors put in the effort to check accuracy of the numbers presented throughout.
Response: To avoid this type of errors, we have sent our revised manuscript to a commercial copy editor in International Science Editing (http://www.internationalscienceediting.com) to improve the language.

The title is misleading and makes the reader think that you have sequenced a unicellular organism.
Response: Thanks for your comment. We have change the title to : “A draft genome assembly of halophyte Suaeda aralocaspica, a plant that performs C_4 photosynthesis within individual cells” to avoid the misunderstanding.

The term 'high quality' is used a lot. To me, this is not helpful. High-quality means different things to different people. The genome presented here is of low or moderate quality to those working in model systems, or those with recently sequenced genomes. However, it is certainly high enough quality for a species with little genomic information. I would drop this term throughout and instead focus on the exact contiguity.
Response: Thanks for you for your comments. We have dropped this term throughout..

Methods:
The methodological details focus on lab protocols, but have very little information about sample sizes, plant growth, etc. for example: How were plants grown for RNA extraction? How many biological replicates? N. technical replicates? Was RNA extracted from the exact same plant as gDNA? If not, was it the same genotype? If so, how was it clonally propagated? All of these questions and many others must be answered.
Please provide georeferenced location information for the sample collection site.
Please provide the collection permits and all other information about how the sample was obtained.

Response: Thanks for you for your comments. We have added the suggested information as a separated part called “Plant materials” at line 92 in revised manuscript.

How were PacBio reads error corrected?

Response: The raw reads with length < 1kb were filtered and then 46 Gb of Illumina clean reads with 100 bp read length was used to correct the PacBio raw reads using Proovread (https://github.com/BioInf-Wuerzburg/proovread). The detailed method were added to the revised manuscript (line 121).

How is % gap calculated? on a per-bp basis? Line 126. If so, how large are the gaps (10k Ns?)

Powered by Editorial Manager® and ProduXion Manager® from Aries Systems Corporation

	Response: A "gap" is a run of Ns of any length, % gap was calculated using total length of Ns divided to the total length of assembly.<br>In the final assembly, there are 35949 Gaps, the total length of Gaps is 13456548, in average each gap is 374 Ns. The results were added to the revised manuscript (line 148).
Additional Information:	
Question	Response
Are you submitting this manuscript to a special series or article collection?	No
**Experimental design and statistics**<br><br>Full details of the experimental design and statistical methods used should be given in the Methods section, as detailed in our Minimum Standards Reporting Checklist. Information essential to interpreting the data presented should be made available in the figure legends.<br><br>Have you included all the information requested in your manuscript?	Yes
**Resources**<br><br>A description of all resources used, including antibodies, cell lines, animals and software tools, with enough information to allow them to be uniquely identified, should be included in the Methods section. Authors are strongly encouraged to cite Research Resource Identifiers (RRIDs) for antibodies, model organisms and tools, where possible.<br><br>Have you included the information requested as detailed in our Minimum Standards Reporting Checklist?	Yes
**Availability of data and materials**<br><br>All datasets and code on which the conclusions of the paper rely must be either included in your submission or deposited in publicly available repositories (where available and ethically	Yes

appropriate), referencing such data using a unique identifier in the references and in the “Availability of Data and Materials” section of your manuscript.<br><br>Have you have met the above requirement as detailed in our Minimum Standards Reporting Checklist?	

Powered by Editorial Manager® and ProduXion Manager® from Aries Systems Corporation

Manuscript

Click here to access/download;Manuscript;manuscript-revision.docx

Click here to view linked References

A draft genome assembly of halophyte *Suaeda aralocaspica*, a plant that performs C_4 photosynthesis within individual cells

Lei Wang[1], Ganglong Ma[2], Hongling Wang[3], Chao Cheng[2], Shuyong Mu[3], Weili Quan[2], Li Jiang[4,5], Zhenyong Zhao[1], Yu Zhang[2], Ke Zhang[1], Xuelian Wang[2], Changyan Tian[1,*], Yi Zhang[2,*]

[1]State Key Laboratory of Desert and Oasis Ecology, Xinjiang Institute of Ecology and Geography, Chinese Academy of Sciences, Urumqi 830011, China,

[2]Center for Genome Analysis, ABLife Inc., Wuhan, Hubei 430075, China,

[3]Central Lab, Xinjiang Institute of Ecology and Geography, Chinese Academy of Sciences, Urumqi 830011, China,

[4]Key Laboratory of Biogeography and Bioresource in Arid Land, Xinjiang Institute of Ecology and Geography, Chinese Academy of Sciences, Urumqi 830011, China,

[5]Turpan Eremophytes Botanical Garden, Chinese Academy of Sciences, Turpan 838008, China,

[*]Correspondence address: Yi Zhang, Center for Genome Analysis, ABLife Inc., Wuhan, Hubei 430075, China, E-mail: yizhang@ablife.cc; Changyan Tian, State Key Laboratory of Desert and Oasis Ecology, Xinjiang Institute of Ecology and Geography, Chinese Academy of Sciences, Urumqi 830011, China, E-mail: tianchy@ms.xjb.ac.cn

Abstract

Background: The halophyte *Suaeda aralocaspica* performs complete C_4 photosynthesis within individual cells (SCC_4), which is distinct from typical C_4 plants that require the collaboration of two types of photosynthetic cells. However, despite SCC_4 plants having features that are valuable in engineering higher photosynthetic efficiencies in C_3 species, including rice, there are no reported sequenced SCC_4 plant genomes, which limits our understanding of the mechanisms involved in, and evolution of, SCC_4 photosynthesis.

Findings: Using Illumina and Pacbio platforms, we generated ~202 Gb of clean genomic DNA sequences having a 433-fold coverage based on the 467 Mb estimated genome size of *S. aralocaspica*. The final genome assembly was 452 Mb, consisting of 4,033 scaffolds, with a scaffold N50 length of 1.83 Mb. We annotated 29,604 protein-coding genes using Evidence Modeler based on the gene information from *ab initio* predictions, homology levels with known genes, and RNA sequencing-based transcriptome evidence. We also annotated noncoding genes, including 1,651 long noncoding RNAs, 21 microRNAs, 382 transfer RNAs, 88 small nuclear RNAs, and 325 ribosomal RNAs. A complete (circular with no gaps) chloroplast genome of *S. aralocaspica* was also assembled to be 146,654 bp in length.

Conclusions: We have presented the genome sequence of *S. aralocaspica*, a SCC_4 plant. Knowledge of the genome of *S. aralocaspica* should increase our understanding of SCC_4 photosynthesis' evolution and contribute to the engineering of C_4 photosynthesis into economically important C_3 crops.

Keywords: *Suaeda aralocaspica*, genome, single-cell C_4, photosynthesis, long noncoding RNAs, halophyte

Background

Carbon loss through photorespiration and water loss through transpiration are common in C_3 plants, especially in warm or dry environments, and they result in significant decreases in growth, water-use efficiency, and harvestable yields [1]. These problems are overcome in C_4 and CAM plant families [2], which perform evolved CO_2-concentrating mechanisms (C_4 cycle) and Calvin cycle (C_3 cycle) using spatial (Kranz structure) and temporal (day to night switch) separations, respectively. Both C_4 and CAM plants can outperform C_3 plants, especially under photorespiratory conditions, and increase the water-use efficiency [2], which has created considerable interest in implementing the C_4 cycle in C_3 crops, such as rice, to improve yields and stress tolerance [3-6].

Among eudicots, C_4 photosynthesis most frequently occurs in the Amaranthaceae of Caryophyllales [7-9]. Four Amaranthaceae species (three *Bienertia* and one *Suaeda*) can perform both C_4 and C_3 cycles within individual photosynthetic cells (single-cell C_4, SCC_4) [10-13]. *Suaeda* contains species that utilize all types of C_4, C_3 and SCC_4 mechanisms for CO_2 fixation and thus, represent a unique genus to study the evolution of C_4 photosynthesis [14]. Mechanistically, the spatially separated chloroplasts in SCC_4 contain different sets of nuclear-encoded proteins that are related to specific functions in the C_4 and C_3 cycles, which biochemically and functionally resemble mesophyll and bundle sheath cells in chloroplasts of Kranz C_4 plant species [10, 11, 15-18]. These findings indicate that the key enzymes in

photosynthesis are conserved and that both C_3 and C_4 enzymes work in the same cells in SCC_4 plants during the day time, which is different from both C_4 and CAM plants.

At present, most knowledge of SCC_4 photosynthesis has come from studies of *Bienertia sinuspersici*, which has two types of chloroplasts distributed in the central and peripheral parts of the cell [16, 18-29]. Studies on *S. aralocaspica* have focused on the germination of dimorphic seeds [30-34]. *S. aralocaspica* has elongated photosynthetic cells with two types of chloroplasts distributed at the opposite ends of the cell. This is analogous to the Kranz anatomy, but lacks the intervening cell wall [35]. This cellular feature indicates that *S. aralocaspica* conducts C_4 and C_3 photosynthesis within a single cell, perhaps retaining the photosynthetic characteristics of both C_4 and C_3 cycles and representing an intermediate model of the evolutionary process from C_3 to C_4 [35, 36]. *S. aralocaspica* is a hygro-halophyte that grows in temperate salt deserts with low night temperatures in areas from northeast of the Caspian lowlands east to Mongolia and western China [35]. Therefore, it is essential to sequence the genome of *S. aralocaspica*, which may aid in studying C_4 evolution under stressful growth conditions and in accelerating engineering C_4 photosynthesis into C_3 crops for adaptation to high saline growth conditions.

In the present study, we sequenced the genome of *S. aralocaspica* collected from a cold desert in the Junggar Basin, Xinjiang, China. Using an integrated assembly strategy that combined shotgun Illumina sequencing and single-molecule real-time sequencing technology from Pacific Biosciences (PacBio), we generated a reference genome assembly of *S. aralocaspica* using protocols established in other plant species [37-40]. To our best knowledge, this is the first sequenced SCC_4 genome. These genomic resources provide a platform for advancing basic biological research and gene discovery in SCC_4 plants, as well as for

engineering C_4 functional modules into C_3 crops to increase yields and to adapt to high-salt conditions.

Data Description

Plant material

First, seeds were collected from a healthy *S. aralocaspica* (Figure 1). The selected plant measured ~40 cm in height and was located within a natural stand close to Fu-kang County, Xinjiang Uygur Autonomous Region, China (N 44°14′ latitude, E 87°40′ longitude, 445 m elevation). The seeds were placed in 0.1% potassium permanganate, washed clean with ultrapure water after 5 min, and then spread in sterilized petri dishes. After a week of 30°C shaded culturing, the seeds germinated. After seed germination, leaves were collected as tissue sources for whole-genome sequencing. In addition, six other healthy *S. aralocaspica* (collected in same location with the plant for seeds collection) were chosen as tissue (mature leaf, stem, root and fruit) sources for RNA sequencing (RNA-seq). The samples were frozen in liquid nitrogen immediately after being collected and then stored at −80°C until DNA/RNA extraction. All the samples were collected with permission from and under the supervision of local forestry bureaus.

DNA extraction and genome sequencing

Genomic DNA was extracted from leaves using a General AllGen Kit (Tiangen biotech, Beijing, China) according to its manufacturer's instructions. Genomic DNA isolated from *S. aralocaspica* was used to construct multiple types of libraries, including short insert size (350, 500, and 800 bp) libraries, mate-paired (2, 5, 10, and 20 kb) libraries, and PacBio single-

molecule real-time cell libraries. The purified libraries were quantified and stored at −80°C before sequencing. Then, the *S. aralocaspica* genome was sequenced on an Illumina sequencing platform (HiSeq 2000) and PacBio RS II platform using eight libraries with different insert sizes. This generated 370 Gb raw Illumina HiSeq data and 10 Gb (~21× genome coverage) PacBio reads (Supplemental Table 1).

To reduce the effects of sequencing errors on the assembly, a series of stringent filtering steps were used during reads generation. We cleaned Illumina reads using the following steps: (1) Cut off adaptors. For the mate-paired library data, reads without Nextera adaptors longer than 10 bp on both end1 and end2 were removed; (2) Remove tail bases with quality score less than 20; (3) Remove reads harboring more than 20% bases with quality scores less than 20; (4) Remove reads with lengths less than 30 nt for DNA-seq; and (5) Remove duplicated paired-end reads from DNA-seq that represent potential PCR artefacts. In total, 1,053,309 raw subreads were produced by Pacbio. Then, reads with lengths < 1 kb were filtered, and 935,509 reads were retained. Next, 46 Gb of Illumina clean reads with 100-bp read lengths was used to correct the PacBio raw reads using Proovread [41] (v2). This yielded 632,805 corrected PacBio reads. After the quality control and filtering steps, 195 Gb clean Illumina reads and 6.9 clean PacBio reads were retained, resulting in a 433× fold coverage of the genome (Supplemental Table 1).

Estimation of genome size

GCE [42] (v1.0.0) was used to estimate the genome size and heterozygosity. The term K-mer refers to a sequence with a length of k bp, and each unique k-mer within a genome dataset can be used to determine the discrete probability distributions of all possible k-mers and their

frequencies of occurrence. Genome size can be calculated using the total length of sequencing reads divided by sequencing depth. To estimate the sequencing depth of the *S. aralocaspica* genome, we counted the copy number of a certain k-mer (e.g., 17-mer) present in the sequence reads and plotted the distribution of the copy numbers. The peak value of the frequency curve represents the overall sequencing depth. We used the algorithm $N \times (L - K + 1)/D = G$, where N represents the total sequence read number, L represents the average length of sequence reads and K represents the k-mer length, which was defined here as 17 bp. G denotes the genome size, and D represents the overall depth estimated from the k-mer distribution. Based on this method, the estimated genome size of *S. aralocaspica* was 467 Mb (Supplemental Figure 1) and the heterozygosity was 0.16%.

Genome assembly

The primary assembled genome was generated by SOAPdenovo [43] (version 2.04-r240) and contained 17,302 initial contigs (N50, ~49.2 kb) and 4,184 scaffolds (N50, ~1.44 Mb) spanning 445.6 Mb, with 96.1 Mb (21.56%) of the total size being intra-scaffold gaps (Supplemental Table 2). Then, we used all of the reads from the short insert libraries to fill gaps using GapCloser [44] (v1.12), and 74.7% of the total gaps were filled. This resulted in a genome size of 424.5 Mb, with 5.92% gaps, which was calculated using the total length of Ns divided by the total length of the assembly. Then, PBJelly [45] (v15.8.24) was used for the second round of gap filling using the polished PacBio data. This finally yielded a ~452 M genome assembly with 4,033 scaffolds (N50, 1.83 Mb) (Table 1, Supplemental Table 2). The assembly spanned 96.8% of the *S. aralocaspica* genome (467 Mb) estimated by the k-mer spectrum (Supplemental Figure 1).

Table 1: Summary of *S. aralocaspica* genome assembly.

Assembly	Illumina	Illumina+PacBio
**Total assembly Size**	424 Mb	452 Mb
**Number of scaffolds (≥ 500 bp)**	4184	4033
**Longest scaffold**	9.29 Mb	9.98 Mb
**N50 contig (size/number)**	49.21 kb/2464	-
**N50 scaffold (size/number)**	1.44 Mb/80	1.83 Mb/67
**N90 scaffold (size/number)**	306.62 kb/332	363.87 kb/282
**% of N**	5.78%	2.98%
**Annotation**		
**Number of protein coding genes**	-	29604
**Number of small RNAs**	-	816
**Number of long non-coding genes**	-	1982

RNA preparation and sequencing

RNA-seq was performed for genome annotation. Different tissues (mature leaf, stem, root, and fruit) of six *S. aralocaspica* were used for RNA extraction. Tissues were ground in liquid nitrogen. After homogenizing the samples in a guanidine thiocyanate extraction buffer, NaAc and chloroform/isoamyl alcohol (24:1) were added. The solution was shaken vigorously, placed on ice for 15 min, and centrifuged (13200rpm) at 4°C to separate a clear upper aqueous layer, from which RNA was precipitated with isopropanol. The precipitated RNA was washed with 75% ethanol to remove impurities and then resuspended with DEPC-treated water. Total RNA

was treated with RQ1 DNase (Promega) to remove DNA. The quality and quantity of the purified RNA were determined by measuring the absorbance at 260 nm/280 nm (A260/A280) using smartspec plus (BioRad). RNA integrity was further verified by 1.5% agarose gel electrophoresis. RNAs were then equally mixed for RNA-seq library preparation. Polyadenylated mRNAs were purified and concentrated with oligo(dT)-conjugated magnetic beads (Invitrogen) before directional RNA-seq library preparation. Purified mRNAs were fragmented at 95°C, followed by end repair and 5′ adaptor ligation. Reverse transcription was performed using an RT primer harboring a 3′ adaptor sequence and a randomized hexamer. The cDNAs were purified and amplified, and PCR products corresponding to 200–500 bp were purified, quantified and stored at −80°C before sequencing. Transcriptomic libraries were sequenced using HiSeq X Ten for paired-end 150-nt reads. As a result, we generated 30 Gb of RNA-seq data (Supplemental Table 3).

To further annotate transcriptional start and termination sites, we also sequenced cap analysis of gene expression and deep sequencing (CAGE) and polyadenylation site sequencing (PAS) data. In brief, 20 μg of total RNA of mature leaves was used for CAGE-seq library preparation. Polyadenylated mRNAs were purified and concentrated with oligo (dT)-conjugated magnetic beads (Invitrogen). After treating with FastAP (Invitrogen) for 1 h at 37°C and subsequently with tobacco acid pyrophosphatase (Ambion) for 1 h at 37°C, the decapped full-length mRNA was ligated to the Truseq 5′ RNA adaptor (Illumina) for 1 h at 37°C and purified with oligo (dT)-conjugated magnetic beads (Invitrogen). Following fragmentation at 95°C, first-strand cDNA was synthesized using an RT primer harboring the Truseq 3′ adaptor sequence (Illumina) and a randomized hexamer. The cDNAs were purified and amplified using

Truseq PCR primers (Illumina), and products corresponding to 200–500 bp were purified, quantified and stored at −80°C until sequencing. CAGE-seq libraries were sequenced with Illumina Nextseq 500 for paired-end 150-nt reads. Finally, 16 Gb of CAGE-seq data were generated (Supplemental Table 3). In addition, 10 μg of total RNA of mature leaves was used for PAS-seq library preparation. In brief, polyadenylated mRNAs were purified using oligo (dT)-conjugated magnetic beads (Invitrogen). Purified RNA was fragmented and then reverse transcription was performed using a PAS-RT primer (a modified Truseq 3′ adaptor harboring dT18 and two additional anchor nucleotides at the 3′ terminus). DNA was then synthesized with Terminal-Tagging oligo cDNA using a ScriptSeq™cv2 RNA-Seq Library Preparation Kit (Epicentre). The cDNAs were purified and amplified, and PCR products corresponding to 300–500 bp were purified, quantified and stored at −80°C before sequencing. PAS-seq libraries were sequenced with Illumina Nextseq 500 for single-end 300-nt reads. Finally, 28.5 Gb of PAS-seq data were generated (Supplemental Table 3).

To annotate microRNA (miRNA), a total of 3 μg of mixed total RNA was the template for a small RNA cDNA library preparation using Balancer NGS Library Preparation Kit for small/microRNA (GnomeGen), following the manufacturer's instructions. Briefly, RNAs were ligated to 3′ and 5′ adaptors sequentially, reverse transcribed to cDNA and PCR amplified. The whole library was applied to 10% native PAGE gel electrophoresis, and bands corresponding to miRNA insertions were cut and eluted. After ethanol precipitation and washing, the purified small RNA libraries were quantified using a Qubit Fluorometer (Invitrogen) and stored at −80°C until sequencing. The small RNA library was sequenced with Illumina GA IIx for 33-nt reads. Finally, 4.5 Gb of small RNA data were generated (Supplemental Table 3).

Genome quality evaluation

Different methods and data were employed to check the completeness of the assembly. Using BWA [46], we found that 87.08%–90.63% of DNA-paired end reads (350, 500, and 800 bp) could be properly mapped to the final assembled genome (Supplemental Table 4, Supplemental Figure 2). We evaluated the completeness of the gene regions in our assembly using BUSCO [47] (v3.0.2). In total, 89.5% of the 1,440 single-copy orthologs presented in the plant lineage was completely identified in the genome (Supplemental Figure 3).

Furthermore, Trinity [48] (r20140413p1) was used to assemble the RNA-seq reads sequenced from the mixed *S. aralocaspica* RNA library into 157,521 unigenes. Then, these unigenes were aligned to the genome assembly by BLASTN with default parameter. We found 94.5% of the unigenes could be aligned to the genome assembly, and 76.3% of the unigenes could cover 90% of the sequence length of one scaffold. For unigenes longer than 1 kb, 99.5% of the unigenes could be aligned to the genome assembly, and 92.8% of the unigenes could cover 90% of the sequence length of one scaffold (Supplemental Table 5).

Gene and functional annotations

The genome of *S. aralocaspica* was annotated for protein-coding genes (PCGs), repeat elements, non-coding genes and other genomic elements. In detail, MAKER [49] (v2.31.9) was used to generate a consensus gene set based on three different type of evidence, *ab initio*, protein homologues, and the transcripts. *De novo* predictions were processed by AUGUSTUS [50] (v3.2.1). Non-redundant protein sequences of seven sequenced plants (*Arabidopsis thaliana*, *Oryza sativa*, *Beta vulgaris*, *Chenopodium quinoa*, *Glycine max*, *Spinacia oleracea*, and *Vitis vinifera*) provided homology evidence. The *S. aralocaspica* RNA-seq data generated from this

study and a published transcriptome of the seed [51] were assembled into unigenes by Trinity [52] as the transcript evidence. We predicted 29,064 PCGs, with an average transcript length of 4,462 bp, coding sequence size of 1,112 bp, and a mean of 4.76 exons per transcript (Supplemental Tables 6 and 7). Of the annotated PCGs, 97.2% were functionally annotated by the InterPro, GO, KEGG, SwissProt or NR databases (Supplemental Figures 4 and 5, Supplemental Table 8), and ~91% were annotated with protein or transcript support (Supplemental Table 9). The transcriptional start and termination sites of most of the annotated genes were supported by sequencing reads from CAGE-seq and PAS-seq (Supplemental Figures 6 and 7).

In addition, 1, 651 long noncoding RNAs were predicted following a previously published method [53]. In total, 382 transfer RNAs (tRNAs) were predicted using tRNAscan-SE [54] (v1.3.1). Additionally, 21 miRNAs, 88 small nuclear RNAs, and 325 ribosomal RNAs, were identified by using the CMscan tool from INFERNAL [55] (v1.1.2) to search the Rfam database with option --cut_ga (Supplemental Table 10, Supplemental Figure 8).

Repeat annotation

To annotate the repeat sequences of the *S. aralocaspica* genome, a combination of *de novo* and homology-based approaches was employed [56, 57]. For homology-based identification, we used RepeatMasker [58] (open-4.0.5) to search the protein database in Rebase against the *S. aralocaspica* genome and identify transposable elements (TEs). The Rebase database (http://www.girinst.org/server/RepBase/index.php) was used to identify TEs. Parameters of RepeatMasker were set to "-species Viridiplantae -pa 30 -e rmblast". In the *de novo* approach, PILER [59] (v1.0) was used to build the consensus repeat database. PILER software requires

PALS, FAMS, and PILER to construct the consensus library. The default parameters of PILER were used. Then, the predicted consensus TEs were classified using RepeatClassifer implemented in the RepeatModeler package [60] (Version 1.0.11). We used RepeatMasker to search the TEs within the database constructed by PILER. Finally, we combined the *de novo* and homolog predictions of repeat elements according to their coordination in the genome, and detected 173.5 Mb repeat elements, which constituted 38.41% of the genome (Supplemental Table 11). As observed in other sequenced genomes [61], long terminal repeats [62] in *S. aralocaspica* occupied the majority (48.5%) of the repeated sequences (Supplemental Table 12).

Phylogenetic placement of *S. aralocaspica*

The OrthoFinder [63] (v2.3.3) clustering method was used to perform orthologous group analyses with complete annotated protein sequences of 18 sequenced plant genomes, eight C_3 species (*Solanum tuberosum*, *S. oleracea*, *B. vulgaris*, *C. quinoa*, *A. thaliana*, *O. sativa*, *Musa acuminata*, and *Physcomitrella patens*), eight C_4 species (*S. aralocaspica*, *Amaranthus hypochondriacus*, *Sorghum bicolor*, *Setaria italica*, *Z. mays*, *Saccharum* spp., *Panicum hallii*, and *Pennisetum glaucum*) and two CAM species (*Ananas comosus* and *Phalaenopsis equestris*). The longest proteins encoded by each gene in all species were selected as input for OrthoFinder with default parameters. In total, 19,324 orthogroups, containing at least two genes, were circumscribed, 11,768 of which contained at least one gene from *S. aralocaspica* (Supplemental Table 13). Of the 29,604 annotated *S. aralocaspica* genes, 23,112 (89%) were classified into orthogroups. In total, 3,895 orthogroups (172,107 genes) were shared among all the genomes analyzed. A total of 70 orthogroups (351 genes) were specific to the assembled *S. aralocaspica*

genome when compared with the other 17 genomes.

With OrthoFinder, 15 single-copy orthologous genes, shared across 18 species, were identified and were aligned with MUSCLE [78] (v3.8.31), employing default settings (see Supplementary File 1 for commands and settings). The concatenated amino acid sequences were trimmed using trimAI [64] (trimal -gt 0.8 -st 0.001 -cons 60) (v1.2rev59) and were further used by ModelFinder to select the best model (JTTDCMut+F+I+G4). Then, the phylogenetic trees were constructed using IQ-Tree [65] (v1.6.10). The aLRT method was used to perform 1,000 bootstrap analyses to test the robustness of each branch. Then, a timetree was inferred using the Realtime method [66, 67] and Ordinary Least Squares estimates of branch lengths. This analysis involved 18 amino acid sequences. There were 4,489 positions in the final dataset. The timetree were constructed using MEGA X [68]. The resulting phylogenetic tree showed that all five Amaranthaceae species were placed in the same clade, among which *A. hypochondriacus* (C_4) was placed as a sister subclade to the other three C_3 species (Figure 3). Moreover, *S. aralocaspica* (SCC_4) was the sister clade of four other species from the Amaranthaceae including *A. hypochondriacus* (C_4) (Figure 3). Our results of phylogenetic analyses were consistent with a previous study on the evolution of *C. quinoa* [69]. Inside of the Amaranthaceae, the close phylogenetic distance between *S. aralocaspica* (SCC_4) and *A. hypochondriacus* (C_4), away from all other C_3 relatives, suggests that these SCC_4 and C_4 photosynthesis might have had independently evolved. Outside of the Amaranthaceae, *S. aralocaspica* (SCC_4) is more closely related to the C_3 than C_4 plants. These findings do not fully support the existing model that *S. aralocaspica* would be an C_3–C_4 intermediate and were on the road toward the C4 plants [35, 36].

Assembly of the *S. aralocaspica* chloroplast genome

Using the short insert size (350 bp) data, a complete (circular with no gaps) chloroplast genome of *S. aralocaspica* was assembled at 146,654 bp in length using NOVOPlasty [70] (v2.7.2). The Rubisco-bis-phosphate oxygenase (RuBP) subunit of *C. quinoa* (GenBank: KY419706.1) was selected as a seed sequence. An initial gene annotation of the genome was performed using GeSeq [71] (https://chlorobox.mpimp-golm.mpg.de/geseq.html). The circular chloroplast genome maps were drawn using the OrganellarGenome DRAW tool [72] (https://chlorobox.mpimp-golm.mpg.de/OGDraw.html), with subsequent manual editing (Figure 3).

Conclusion

Using the Illumina and Pacbio platforms, we successfully assembled the genome of *S. aralocaspica*, which was the first sequenced genome of a SCC_4 plant. The final genome assembly was 452 Mb and consisted of 4,033 scaffolds, with a scaffold N50 length of 1.83 Mb. We annotated 29,604 protein-coding genes and noncoding genes including 1,651 long noncoding RNAs, 21 miRNAs, 382 tRNAs, 88 small nuclear RNAs, and 325 ribosomal RNAs. The phylogenetic tree placed SCC_4 in a clade more closely related to the C_3 than C_4 plants, do not fully supporting the hypothesis that SCC_4 is a C_3–C_4 intermediate that independently evolved from the C_3 ancestors. A complete (circular with no gaps) chloroplast genome of *S. aralocaspica* was also assembled at 146,654 bp. The available genome assembly, together with transcriptomic data of *S. aralocaspica*, provide a valuable resource for investigating C_4

evolution and mechanisms. We anticipate that future studies of *S. aralocaspica* will greatly facilitate the process of engineering crops, especially C_3 species, including rice, with higher photosynthetic efficiencies and saline tolerance.

Availability of supporting data

Raw sequencing data are deposited in the Sequence Read Archive with accession number SRP128359. The NCBI Bioproject accession is PRJNA428881.

Additional files

Supplemental Figure 1. K-mer distribution of sequencing reads.

Supplemental Figure 2. Size distribution of inserts in sequenced paired-end DNA reads.

Supplemental Figure 3. Integrity comparison of genome assemblies of *S. aralocaspica* with BUSCO. For S. aralocaspica, assemblies in each steps were analyzed respectively.

Supplemental Figure 4. Annotated genes supported by different manners.

Supplemental Figure 5. Gene ontology distribution of *S. aralocaspica* protein coding genes.

Supplemental Figure 6. Transcription start site (TSS) annotation with Cage-seq.

Supplemental Figure 7. Transcription terminal site (TTS) annotation with Pas-seq.

Supplemental Figure 8. Non-coding RNAs classification in S. aralocaspica.

Supplemental Table 1. Summary of sequencing data obtained for genome assembly.

Supplemental Table 2. The assembly statistics of the *S. aralocaspica* genome.

Supplemental Table 3. Information of different types of RNA libraries.

Supplemental Table 4. Mapping efficiency of short insert library reads

Supplemental Table 5. Assessment of sequence coverage of *S. aralocaspica* genome assembly using unigenes.

Supplemental Table 6. Gene prediction in the *S. aralocaspica* genome.

Supplemental Table 7. Comparison of the gene structure among *S. aralocaspica* and some other species

Supplemental Table 8. Summary of *S. aralocaspica* gene annotation based on homology or functional classification.

Supplemental Table 9. Number of *S. aralocaspica* genes with protein or unigene support.

Supplemental Table 10. Noncoding RNA genes in the *S. aralocaspica* genome.

Supplemental Table 11. Repeat elements in the *S. aralocaspica* genome. Repeat elements were identified by different methods and then combined into a final repeat set.

Supplemental Table 12. Repeat elements in *S. aralocaspica* genomes.

Supplemental Table 13. Orthogroups clustered by OrthoFinder in 18 species.

Abbreviations

SCC_4: single-cell C_4 photosynthesis; CAM: crassulacean acid metabolism; lncRNA: long non-coding RNAs; PCG: protein-coding gene;

Competing interests

The authors declare that they have no competing interests.

Funding

This research was supported by the Key Research and Development Program of Xinjiang province (2018B01006-4), the National Natural Science Foundation of China (31770451), the National Key Research and Development Program (2016YFC0501400) and ABLife (ABL2014-02028).

Author contributions

C.T., L.W., Yi Z., and S.M. initiated the project and designed the study. L.W., H.W., L.J., Z.Z., and K.Z. prepared experimental materials and performed experiments for data collection. G.M., C.C., Yu Z., H.W., L.J., and K.Z. assembled the genome, analyzed the data and generated the graphs. Yi Z., W.Q., C.T., L.W., C.C., and X.W. wrote the manuscript.

References

1. Walker BJ, VanLoocke A, Bernacchi CJ and Ort DR. The Costs of Photorespiration to Food Production Now and in the Future. Annual Review of Plant Biology. 2016;67 1:107-29. doi:10.1146/annurev-arplant-043015-111709.
2. Yamori W, Hikosaka K and Way DA. Temperature response of photosynthesis in C_3, C_4, and CAM plants: temperature acclimation and temperature adaptation. Photosynthesis Res. 2014;119 1-2:101-17. doi:10.1007/s11120-013-9874-6.
3. Hibberd JM, Sheehy JE and Langdale JA. Using C_4 photosynthesis to increase the yield of rice-rationale and feasibility. Curr Opin Plant Biol. 2008;11 2:228-31. doi:https://doi.org/10.1016/j.pbi.2007.11.002.
4. von Caemmerer S, Quick WP and Furbank RT. The Development of C4 Rice: Current

Progress and Future Challenges. Science. 2012;336 6089:1671-2. doi:10.1126/science.1220177.

5. Gu J-F, Qiu M and Yang J-C. Enhanced tolerance to drought in transgenic rice plants overexpressing C4 photosynthesis enzymes. The Crop Journal. 2013;1 2:105-14. doi:https://doi.org/10.1016/j.cj.2013.10.002.

6. Betti M, Bauwe H, Busch FA, Fernie AR, Keech O, Levey M, et al. Manipulating photorespiration to increase plant productivity: recent advances and perspectives for crop improvement. Journal of Experimental Botany. 2016;67 10:2977-88. doi:10.1093/jxb/erw076.

7. Akhani H, Trimborn P and Ziegler H. Photosynthetic pathways in Chenopodiaceae from Africa, Asia and Europe with their ecological, phytogeographical and taxonomical importance. Plant Syst Evol. 1997;206 1:187-221. doi:10.1007/bf00987948.

8. Sage RF, Li M and Monson RK. The taxonomic distribution of C_4 photosynthesis. In: Sage RF and Monson RK, editors. C_4 plant biology. San Diego, California, USA: Academic Press; 1999. p. 551-84.

9. Jacobs SWL. Review of leaf anatomy and ultrastructure in the Chenopodiaceae (Caryophyllales). J Torrey Bot Soc. 2001;128 3:236-53.

10. Voznesenskaya EV, Franceschi VR, Kiirats O, Freitag H and Edwards GE. Kranz anatomy is not essential for terrestrial C4 plant photosynthesis. Nature. 2001;414 6863:543-6. doi:10.1038/35107073.

11. Voznesenskaya EV, Franceschi VR, Kiirats O, Artyusheva EG, Freitag H and Edwards GE. Proof of C4 photosynthesis without Kranz anatomy in Bienertia cycloptera

(Chenopodiaceae). The Plant Journal. 2002;31 5:649-62. doi:10.1046/j.1365-313X.2002.01385.x.

12. Akhani H, Barroca J, Koteeva N, Voznesenskaya E, Franceschi V, Edwards G, et al. Bienertia sinuspersici (Chenopodiaceae): A New Species from Southwest Asia and Discovery of a Third Terrestrial C4 Plant Without Kranz Anatomy. Systematic Botany. 2005;30 2:290-301. doi:10.1600/0363644054223684.

13. Akhani H, Chatrenoor T, Dehghani M, Khoshravesh R, Mahdavi P and Matinzadeh Z. A new species of *Bienertia* (Chenopodiaceae) from Iranian salt deserts: A third species of the genus and discovery of a fourth terrestrial C_4 plant without Kranz anatomy. Plant Biosystems. 2012;146 3:550-9. doi:10.1080/11263504.2012.662921.

14. Schütze P, Freitag H and Weising K. An integrated molecular and morphological study of the subfamily Suaedoideae Ulbr. (Chenopodiaceae). Plant Syst Evol. 2003;239 3:257-86. doi:10.1007/s00606-003-0013-2.

15. Voznesenskaya EV, Edwards GE, Kiirats O, Artyusheva EG and Franceschi VR. Development of biochemical specialization and organelle partitioning in the single-cell C_4 system in leaves of *Borszczowia aralocaspica* (Chenopodiaceae). Am J Bot. 2003;90 12:1669-80. doi:10.3732/ajb.90.12.1669.

16. Voznesenskaya EV, Koteyeva NK, Chuong SD, Akhani H, Edwards GE and Franceschi VR. Differentiation of cellular and biochemical features of the single-cell C_4 syndrome during leaf development in *Bienertia cycloptera* (Chenopodiaceae). Am J Bot. 2005;92 11:1784-95. doi:10.3732/ajb.92.11.1784.

17. Offermann S, Okita TW and Edwards GE. Resolving the compartmentation and

function of C_4 photosynthesis in the single-cell C_4 species *Bienertia sinuspersici*. Plant Physiol. 2011;155 4:1612-28. doi:10.1104/pp.110.170381.

18. Offermann S, Friso G, Doroshenk KA, Sun Q, Sharpe RM, Okita TW, et al. Developmental and subcellular organization of single-cell C_4 photosynthesis in *Bienertia sinuspersici* determined by large-scale proteomics and cDNA assembly from 454 DNA sequencing. Journal of proteome research. 2015;14 5:2090-108. doi:10.1021/pr5011907.

19. Wimmer D, Bohnhorst P, Shekhar V, Hwang I and Offermann S. Transit peptide elements mediate selective protein targeting to two different types of chloroplasts in the single-cell C_4 species *Bienertia sinuspersici*. Sci Rep. 2017;7:41187. doi:10.1038/srep41187.

20. Jurić I, González-Pérez V, Hibberd JM, Edwards G and Burroughs NJ. Size matters for single-cell C4 photosynthesis in Bienertia. Journal of Experimental Botany. 2017;68 2:255-67. doi:10.1093/jxb/erw374.

21. Stutz SS, Edwards GE and Cousins AB. Single-cell C_4 photosynthesis: efficiency and acclimation of *Bienertia sinuspersici* to growth under low light. The New phytologist. 2014;202 1:220-32. doi:10.1111/nph.12648.

22. Lung SC, Yanagisawa M and Chuong SD. Protoplast isolation and transient gene expression in the single-cell C_4 species, *Bienertia sinuspersici*. Plant Cell Rep. 2011;30 4:473-84. doi:10.1007/s00299-010-0953-2.

23. Leisner CP, Cousins AB, Offermann S, Okita TW and Edwards GE. The effects of salinity on photosynthesis and growth of the single-cell C_4 species *Bienertia*

sinuspersici (Chenopodiaceae). Photosynthesis Res. 2010;106 3:201-14. doi:10.1007/s11120-010-9595-z.

24. Uzilday B, Ozgur R, Yalcinkaya T, Turkan I and Sekmen AH. Changes in redox regulation during transition from C_3 to single cell C_4 photosynthesis in Bienertia sinuspersici. J Plant Physiol. 2017;220:1-10. doi:10.1016/j.jplph.2017.10.006.

25. Koteyeva NK, Voznesenskaya EV, Berry JO, Cousins AB and Edwards GE. The unique structural and biochemical development of single cell C_4 photosynthesis along longitudinal leaf gradients in *Bienertia sinuspersici* and *Suaeda aralocaspica* (Chenopodiaceae). J Exp Bot. 2016;67 9:2587-601. doi:10.1093/jxb/erw082.

26. Rosnow J, Yerramsetty P, Berry JO, Okita TW and Edwards GE. Exploring mechanisms linked to differentiation and function of dimorphic chloroplasts in the single cell C4 species *Bienertia sinuspersici*. BMC Plant Biol. 2014;14:34. doi:10.1186/1471-2229-14-34.

27. Park J, Knoblauch M, Okita TW and Edwards GE. Structural changes in the vacuole and cytoskeleton are key to development of the two cytoplasmic domains supporting single-cell C(4) photosynthesis in Bienertia sinuspersici. Planta. 2009;229 2:369-82. doi:10.1007/s00425-008-0836-8.

28. Lara MV, Offermann S, Smith M, Okita TW, Andreo CS and Edwards GE. Leaf development in the single-cell C_4 system in *Bienertia sinuspersici*: expression of genes and peptide levels for C_4 metabolism in relation to chlorenchyma structure under different light conditions. Plant Physiol. 2008;148 1:593-610. doi:10.1104/pp.108.124008.

29. Chuong SD, Franceschi VR and Edwards GE. The cytoskeleton maintains organelle partitioning required for single-cell C4 photosynthesis in Chenopodiaceae species. Plant Cell. 2006;18 9:2207-23. doi:10.1105/tpc.105.036186.

30. Wang L, Huang Z, Baskin CC, Baskin JM and Dong M. Germination of dimorphic seeds of the desert annual halophyte *Suaeda aralocaspica* (Chenopodiaceae), a C_4 plant without Kranz anatomy. Ann Bot. 2008;102 5:757-69. doi:10.1093/aob/mcn158.

31. Wang L, Baskin JM, Baskin CC, Cornelissen JH, Dong M and Huang Z. Seed dimorphism, nutrients and salinity differentially affect seed traits of the desert halophyte *Suaeda aralocaspica* via multiple maternal effects. BMC Plant Biol. 2012;12:170. doi:10.1186/1471-2229-12-170.

32. Cao J, Lv XY, Chen L, Xing JJ and Lan HY. Effects of salinity on the growth, physiology and relevant gene expression of an annual halophyte grown from heteromorphic seeds. AoB Plants. 2015;7:plv112. doi:10.1093/aobpla/plv112.

33. Wang HL, Tian CY and Wang L. Germination of dimorphic seeds of *Suaeda aralocaspica* in response to light and salinity conditions during and after cold stratification. PeerJ. 2017;5:e3671. doi:10.7717/peerj.3671.

34. Wang L, Wang HL, Yin L and Tian CY. Transcriptome assembly in *Suaeda aralocaspica* to reveal the distinct temporal gene/miRNA alterations between the dimorphic seeds during germination. BMC Genomics. 2017;18:806. doi:10.1186/s12864-017-4209-1.

35. Edwards GE and Voznesenskaya EV. C_4 photosynthesis: Kranz forms and single-cell C_4 in terrestrial plants. In: Raghavendra AS and Sage RF, editors. C_4 photosynthesis

and related CO_2 concentrating mechanisms. Dordrecht: Springer Netherlands; 2011. p. 29-61.

36. Sharpe RM and Offermann S. One decade after the discovery of single-cell C_4 species in terrestrial plants: what did we learn about the minimal requirements of C_4 photosynthesis? Photosynthesis Res. 2014;119 1-2:169-80. doi:10.1007/s11120-013-9810-9.

37. Badouin H, Gouzy J, Grassa CJ, Murat F, Staton SE, Cottret L, et al. The sunflower genome provides insights into oil metabolism, flowering and Asterid evolution. Nature. 2017;546 7656:148-52. doi:10.1038/nature22380.

38. Jarvis DE, Ho YS, Lightfoot DJ, Schmöckel SM, Li B, Borm TJA, et al. The genome of Chenopodium quinoa. Nature. 2017;542:307. doi:10.1038/nature21370 https://www.nature.com/articles/nature21370#supplementary-information.

39. Zhang GQ, Liu KW, Li Z, Lohaus R, Hsiao YY, Niu SC, et al. The Apostasia genome and the evolution of orchids. Nature. 2017;549 7672:379-83. doi:10.1038/nature23897.

40. Zhao G, Zou C, Li K, Wang K, Li T, Gao L, et al. The *Aegilops tauschii* genome reveals multiple impacts of transposons. Nature Plants. 2017; doi:10.1038/s41477-017-0067-8.

41. Hackl T, Hedrich R, Schultz J and Forster F. proovread: large-scale high-accuracy PacBio correction through iterative short read consensus. Bioinformatics. 2014;30 21:3004-11. doi:10.1093/bioinformatics/btu392.

42. Liu B, Shi Y, Yuan J, Hu X, Zhang H, Li N, et al. Estimation of genomic characteristics by analyzing k-mer frequency in de novo genome projects. arXiv: Genomics. 2013.

43. Li R, Zhu H, Ruan J, Qian W, Fang X, Shi Z, et al. De novo assembly of human genomes with massively parallel short read sequencing. Genome Res. 2010;20 2:265-72. doi:10.1101/gr.097261.109.

44. The-Tomato-Genome-Consortium. The tomato genome sequence provides insights into fleshy fruit evolution. Nature. 2012;485:635. doi:10.1038/nature11119 https://www.nature.com/articles/nature11119#supplementary-information.

45. English AC, Richards S, Han Y, Wang M, Vee V, Qu J, et al. Mind the gap: upgrading genomes with Pacific Biosciences RS long-read sequencing technology. PLoS One. 2012;7 11:e47768. doi:10.1371/journal.pone.0047768.

46. Li H and Durbin R. Fast and accurate short read alignment with Burrows-Wheeler transform. Bioinformatics. 2009;25 14:1754-60. doi:10.1093/bioinformatics/btp324.

47. Simao FA, Waterhouse RM, Ioannidis P, Kriventseva EV and Zdobnov EM. BUSCO: assessing genome assembly and annotation completeness with single-copy orthologs. Bioinformatics. 2015;31 19:3210-2. doi:10.1093/bioinformatics/btv351.

48. Grabherr MG, Haas BJ, Yassour M, Levin JZ, Thompson DA, Amit I, et al. Full-length transcriptome assembly from RNA-Seq data without a reference genome. Nat Biotechnol. 2011;29 7:644-52. doi:10.1038/nbt.1883.

49. Cantarel BL, Korf I, Robb SM, Parra G, Ross E, Moore B, et al. MAKER: an easy-to-use annotation pipeline designed for emerging model organism genomes. Genome Res. 2008;18 1:188-96. doi:10.1101/gr.6743907.

50. Stanke M and Morgenstern B. AUGUSTUS: a web server for gene prediction in eukaryotes that allows user-defined constraints. Nucleic Acids Res. 2005;33 Web

Server issue:W465-7. doi:10.1093/nar/gki458.

51. Wang L, Wang HL, Yin L and Tian CY. Transcriptome assembly in Suaeda aralocaspica to reveal the distinct temporal gene/miRNA alterations between the dimorphic seeds during germination. BMC Genomics. 2017;18 1:806. doi:10.1186/s12864-017-4209-1.

52. Grabherr MG, Haas BJ, Yassour M, Levin JZ, Thompson DA, Amit I, et al. Full-length transcriptome assembly from RNA-Seq data without a reference genome. Nat Biotechnol. 2011;29 7:644-52. doi:10.1038/nbt.1883.

53. Cabili MN, Trapnell C, Goff L, Koziol M, Tazon-Vega B, Regev A, et al. Integrative annotation of human large intergenic noncoding RNAs reveals global properties and specific subclasses. Genes Dev. 2011;25 18:1915-27. doi:10.1101/gad.17446611.

54. Lowe TM and Eddy SR. tRNAscan-SE: a program for improved detection of transfer RNA genes in genomic sequence. Nucleic Acids Res. 1997;25 5:955-64.

55. Nawrocki EP, Kolbe DL and Eddy SR. Infernal 1.0: inference of RNA alignments. Bioinformatics. 2009;25 10:1335-7. doi:10.1093/bioinformatics/btp157.

56. Iorizzo M, Senalik DA, Grzebelus D, Bowman M, Cavagnaro PF, Matvienko M, et al. De novo assembly and characterization of the carrot transcriptome reveals novel genes, new markers, and genetic diversity. BMC Genomics. 2011;12:389. doi:10.1186/1471-2164-12-389.

57. Wang L, Yu S, Tong C, Zhao Y, Liu Y, Song C, et al. Genome sequencing of the high oil crop sesame provides insight into oil biosynthesis. Genome Biology. 2014;15 2:R39. doi:10.1186/gb-2014-15-2-r39.

58. Tarailo-Graovac M and Chen N. Using RepeatMasker to identify repetitive elements in genomic sequences. Current protocols in bioinformatics. 2009;Chapter 4:Unit 4.10. doi:10.1002/0471250953.bi0410s25.

59. Edgar RC and Myers EW. PILER: identification and classification of genomic repeats. Bioinformatics. 2005;21 Suppl 1:i152-i8. doi:10.1093/bioinformatics/bti1003.

60. Rao SK, Fukayama H, Reiskind JB, Miyao M and Bowes G. Identification of C4 responsive genes in the facultative C4 plant *Hydrilla verticillata*. Photosynthesis Res. 2006;88 2:173-83. doi:10.1007/s11120-006-9049-9.

61. Vlasova A, Capella-Gutierrez S, Rendon-Anaya M, Hernandez-Onate M, Minoche AE, Erb I, et al. Genome and transcriptome analysis of the Mesoamerican common bean and the role of gene duplications in establishing tissue and temporal specialization of genes. Genome Biology. 2016;17:32. doi:10.1186/s13059-016-0883-6.

62. Wicker T, Sabot F, Hua-Van A, Bennetzen JL, Capy P, Chalhoub B, et al. A unified classification system for eukaryotic transposable elements. Nature reviews Genetics. 2007;8 12:973-82. doi:10.1038/nrg2165.

63. Emms DM and Kelly S. OrthoFinder: solving fundamental biases in whole genome comparisons dramatically improves orthogroup inference accuracy. Genome Biol. 2015;16:157. doi:10.1186/s13059-015-0721-2.

64. Capella-Gutierrez S, Silla-Martinez JM and Gabaldon T. trimAl: a tool for automated alignment trimming in large-scale phylogenetic analyses. Bioinformatics. 2009;25 15:1972-3. doi:10.1093/bioinformatics/btp348.

65. Nguyen LT, Schmidt HA, von Haeseler A and Minh BQ. IQ-TREE: a fast and effective

stochastic algorithm for estimating maximum-likelihood phylogenies. Mol Biol Evol. 2015;32 1:268-74. doi:10.1093/molbev/msu300.

66. Tamura K, Tao Q and Kumar S. Theoretical Foundation of the RelTime Method for Estimating Divergence Times from Variable Evolutionary Rates. Mol Biol Evol. 2018;35 7:1770-82. doi:10.1093/molbev/msy044.

67. Tamura K, Battistuzzi FU, Billing-Ross P, Murillo O, Filipski A and Kumar S. Estimating divergence times in large molecular phylogenies. Proc Natl Acad Sci U S A. 2012;109 47:19333-8. doi:10.1073/pnas.1213199109.

68. Kumar S, Stecher G, Li M, Knyaz C and Tamura K. MEGA X: Molecular Evolutionary Genetics Analysis across Computing Platforms. Mol Biol Evol. 2018;35 6:1547-9. doi:10.1093/molbev/msy096.

69. Zou C, Chen A, Xiao L, Muller HM, Ache P, Haberer G, et al. A high-quality genome assembly of quinoa provides insights into the molecular basis of salt bladder-based salinity tolerance and the exceptional nutritional value. Cell Res. 2017;27:1327. doi:10.1038/cr.2017.124 https://www.nature.com/articles/cr2017124#supplementary-information.

70. Dierckxsens N, Mardulyn P and Smits G. NOVOPlasty: de novo assembly of organelle genomes from whole genome data. Nucleic Acids Res. 2017;45 4:e18. doi:10.1093/nar/gkw955.

71. Tillich M, Lehwark P, Pellizzer T, Ulbricht-Jones ES, Fischer A, Bock R, et al. GeSeq - versatile and accurate annotation of organelle genomes. Nucleic Acids Res. 2017;45 W1:W6-W11. doi:10.1093/nar/gkx391.

72. Lohse M, Drechsel O, Kahlau S and Bock R. OrganellarGenomeDRAW--a suite of tools for generating physical maps of plastid and mitochondrial genomes and visualizing expression data sets. Nucleic Acids Res. 2013;41 Web Server issue:W575-81. doi:10.1093/nar/gkt289.

73. Kapralov MV, Akhani H, Voznesenskaya EV, Edwards G, Franceschi V and Roalson EH. Phylogenetic Relationships in the Salicornioideae / Suaedoideae / Salsoloideae s.l. (Chenopodiaceae) Clade and a Clarification of the Phylogenetic Position of Bienertia and Alexandra Using Multiple DNA Sequence Datasets. Systematic Botany. 2006;31 3:571-85. doi:10.1043/06-01.1.

Figure legend

Figure 1: Example of *S. aralocaspica.*

Figure 2: The phylogenetic relationship of the *S. aralocaspica* with other C_3/C_4/CAM plants.

Figure 3: Gene map of S. aralocaspica chloroplast genome. Genes shown outside the outer circle are transcribed clockwise, and those inside are transcribed counterclockwise. Genes belonging to different functional groups are color coded. The dashed area in the inner circle indicates GC content of the chloroplast genome.

Figure 1

Click here to access/download;Figure;Figure.1.pdf

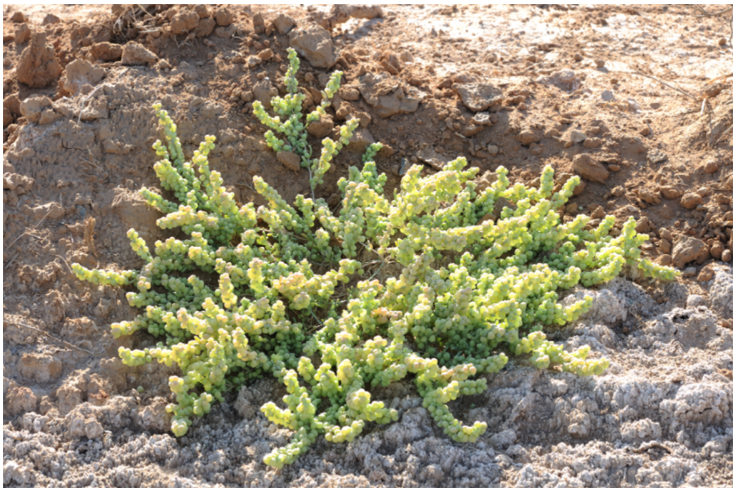

Figure 2

Click here to access/download;Figure;Figure.2.pdf

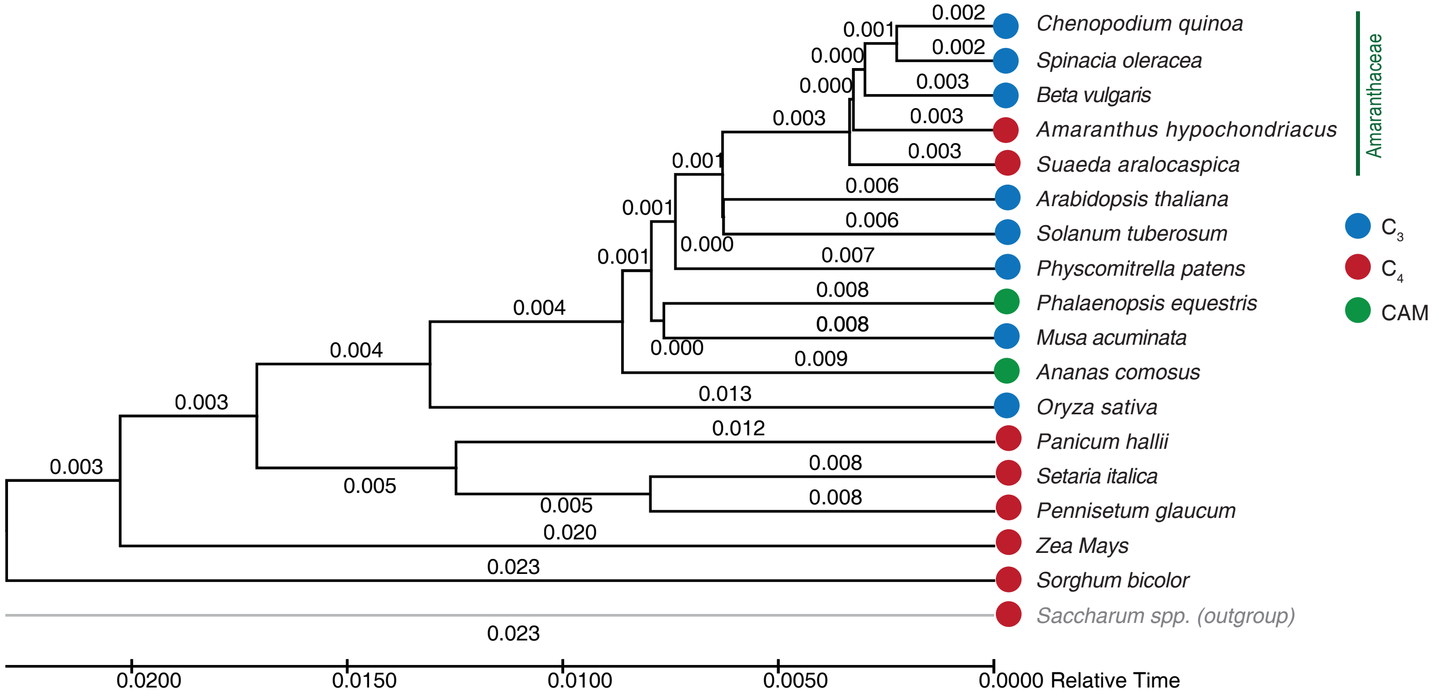

Figure 3

Click here to access/download;Figure;Figure.3.pdf

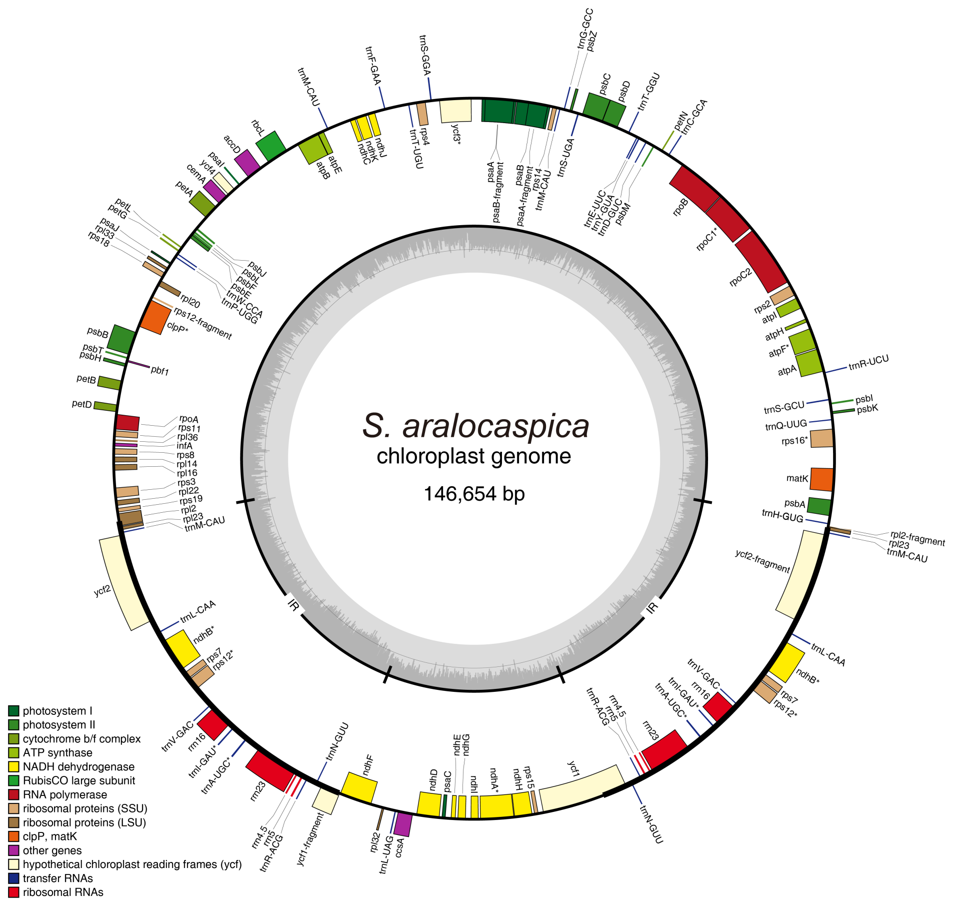

Click here to access/download
Supplementary Material
Supplemental-Information-revision.docx
