# Supplementary material for: A draft genome assembly of halophyte Suaeda aralocaspica, a plant that performs C4 photosynthesis within individual cells
Source: Gigascience. 2019 Sep 12;8(9):giz116. doi: 10.1093/gigascience/giz116 (PMC6741815; doi:10.1093/gigascience/giz116)
Supplement: giz116_Supplemental_Figures_and_Tables [file giz116_supplemental_figures_and_tables.docx]

**Supplemental Information For**

**A draft genome assembly of halophyte *Suaeda aralocaspica*, a plant that performs C_4_ photosynthesis within individual cells**

Lei Wang^1^, Ganglong Ma^2^, Hongling Wang^3^, Chao Cheng^2^, Shuyong Mu^3^, Weili Quan^2^, Li Jiang^4,5^, Zhenyong Zhao^1^, Yu Zhang^2^, Ke Zhang^1^, Xuelian Wang^2^, Changyan Tian^1,*^, Yi Zhang^2,*^

^1^State Key Laboratory of Desert and Oasis Ecology, Xinjiang Institute of Ecology and Geography, Chinese Academy of Sciences, Urumqi 830011, China,

^2^Center for Genome Analysis, ABLife Inc., Wuhan, Hubei 430075, China,

^3^Central Lab, Xinjiang Institute of Ecology and Geography, Chinese Academy of Sciences, Urumqi 830011, China,

^4^Key Laboratory of Biogeography and Bioresource in Arid Land, Xinjiang Institute of Ecology and Geography, Chinese Academy of Sciences, Urumqi 830011, China,

^5^Turpan Eremophytes Botanical Garden, Chinese Academy of Sciences, Turpan 838008, China,

^*^Correspondence address: Yi Zhang, Center for Genome Analysis, ABLife Inc., Wuhan, Hubei 430075, China, E-mail: [yizhang@ablife.cc](mailto:yizhang@ablife.cc); Changyan Tian, State Key Laboratory of Desert and Oasis Ecology, Xinjiang Institute of Ecology and Geography, Chinese Academy of Sciences, Urumqi 830011, China, E-mail: [tianchy@ms.xjb.ac.cn](mailto:tianchy@ms.xjb.ac.cn)

Table of Contents

[Supplemental Figures 2](#_Toc13661261)

[Supplemental Figure 1. *K-mer* distribution of sequencing reads. 3](#_Toc13661262)

[Supplemental Figure 2. Size distribution of inserts in sequenced paired-end DNA reads. 4](#_Toc13661263)

[Supplemental Figure 3. Integrity comparison of genome assemblies of *S. aralocaspica* with BUSCO. For *S. aralocaspica*, assemblies in each steps were analyzed respectively. 5](#_Toc13661264)

[Supplemental Figure 4. Annotated genes supported by different manners. 6](#_Toc13661265)

[Supplemental Figure 5. Gene ontology distribution of *S. aralocaspica* protein coding genes. 7](#_Toc13661266)

[Supplemental Figure 6. Transcription start site (TSS) annotation with Cage-seq. 8](#_Toc13661267)

[Supplemental Figure 7. Transcription terminal site (TTS) annotation with Pas-seq. 9](#_Toc13661268)

[Supplemental Figure 8. Non-coding RNAs classification in *S. aralocaspica*. 10](#_Toc13661269)

[Supplementary Tables 11](#_Toc13661270)

[Supplemental Table 1. Summary of sequencing data obtained for genome assembly. 11](#_Toc13661271)

[Supplemental Table 2. The assembly statistics of the *S. aralocaspica* genome. 12](#_Toc13661272)

[Supplemental Table 3. Information of different types of RNA libraries. 13](#_Toc13661273)

[Supplemental Table 4. Mapping efficiency of short insert library reads 14](#_Toc13661274)

[Supplemental Table 5. Assessment of sequence coverage of S. *aralocaspica* genome assembly using unigenes. 15](#_Toc13661275)

[Supplemental Table 6. Gene prediction in the *S. aralocaspica* genome. 16](#_Toc13661276)

[Supplemental Table 7. Comparison of the gene structure among *S. aralocaspica* and some other species 17](#_Toc13661277)

[Supplemental Table 8. Summary of *S. aralocaspica* gene annotation based on homology or functional classification. 18](#_Toc13661278)

[Supplemental Table 9. Number of *S. aralocaspica* genes with protein or unigene support. 19](#_Toc13661279)

[Supplemental Table 10. Noncoding RNA genes in the *S. aralocaspica* genome. 20](#_Toc13661280)

[Supplemental Table 11. Repeat elements in the *S. aralocaspica* genome. Repeat elements were identified by different methods and then combined into a final repeat set. 21](#_Toc13661281)

[Supplemental Table 12. Repeat elements in *S. aralocaspica* genomes. 22](#_Toc13661282)

[Supplemental Table 13. Orthogroups clustered by OrthoFinder in 18 species. 23](#_Toc13661283)

# Supplemental Figures


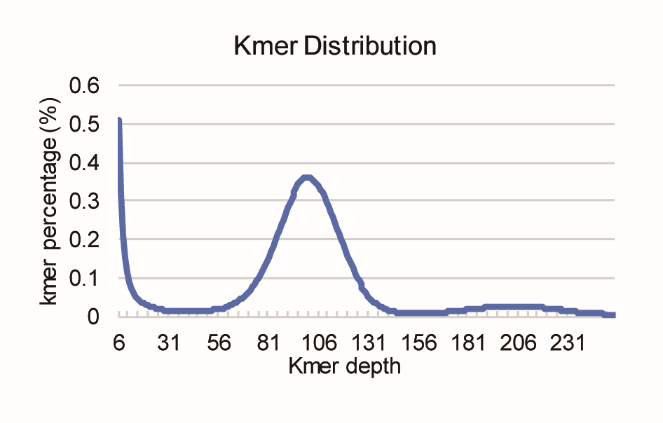


## Supplemental Figure 1. *K-mer* distribution of sequencing reads.

According to the distribution, we estimate that the genome size of *S. aralocaspica* is approximately 467M. The analysis is based on the Illumina data.


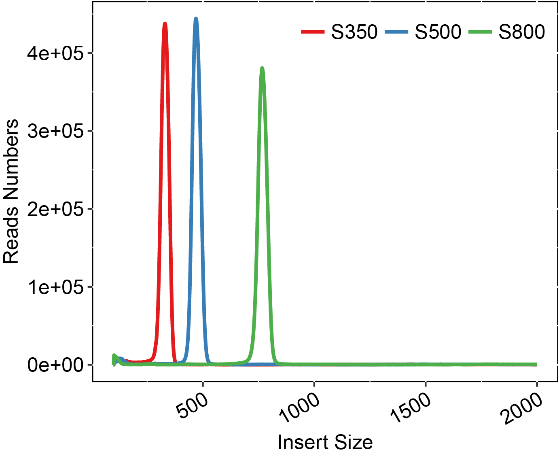


## Supplemental Figure 2. Size distribution of inserts in sequenced paired-end DNA reads.

All reads from the short insert libraries (350bp, 500bp, 800bp) were mapped onto the *S. aralocaspica* genome by BWA. The real insert size for each read was extracted, then the number of reads with specific insert size was calculated.


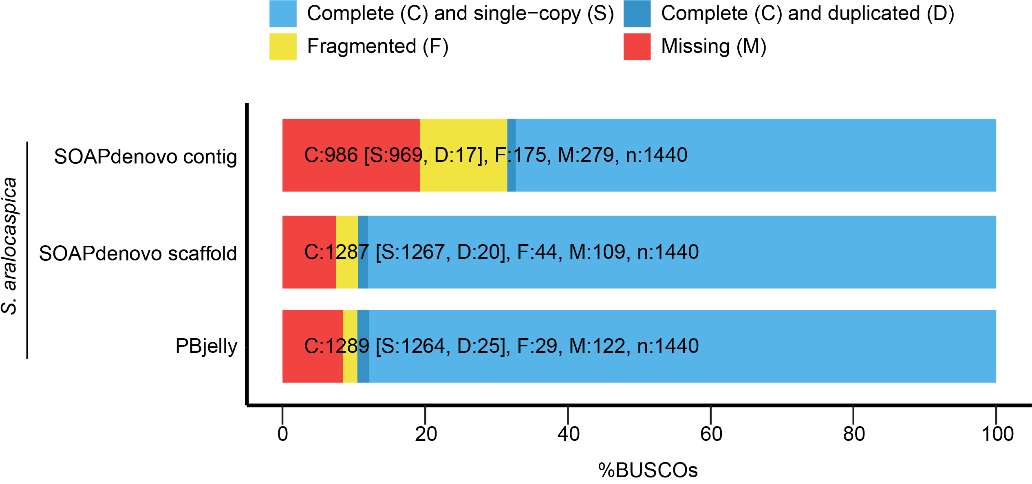


## Supplemental Figure 3. Integrity comparison of genome assemblies of *S. aralocaspica* with BUSCO. For *S. aralocaspica*, assemblies in each steps were analyzed respectively.


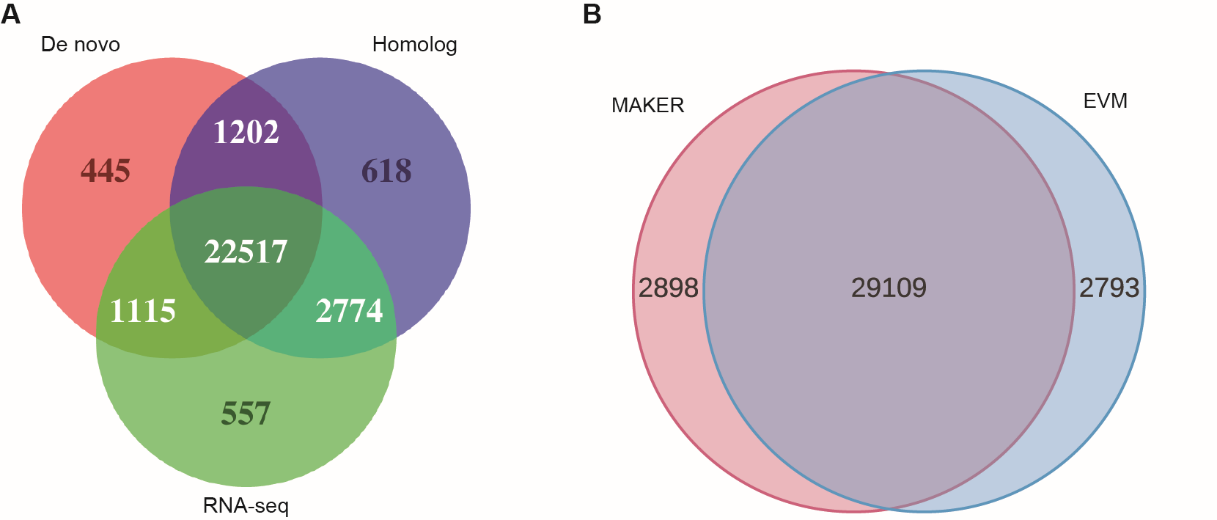


## Supplemental Figure 4. Annotated genes supported by different manners.

**A, Number of genes with protein or unigene support.** Protein database: KEGG, Swiss-Prot, NR, InterproScan; Protein support criterion: identity ≥ 30%, e-value < 1e-5. RNA-Seq clean data was assembled to unigenes by Trinity. GeneMark and GlimmerHmm were used for *ab initio* prediction, genes support criterion: identity ≥ 30%, e value < 1 e-5.

**B, Gene number supported by interpro database in the results by MAKER and EVM.** Protein coding gene number predicted by MAKER and EVM were blast to interpro database, using support criterion: identity ≥ 30%, e value < 1 e-5, 29109 proteins from the database both support the results from MAKER and EVM.


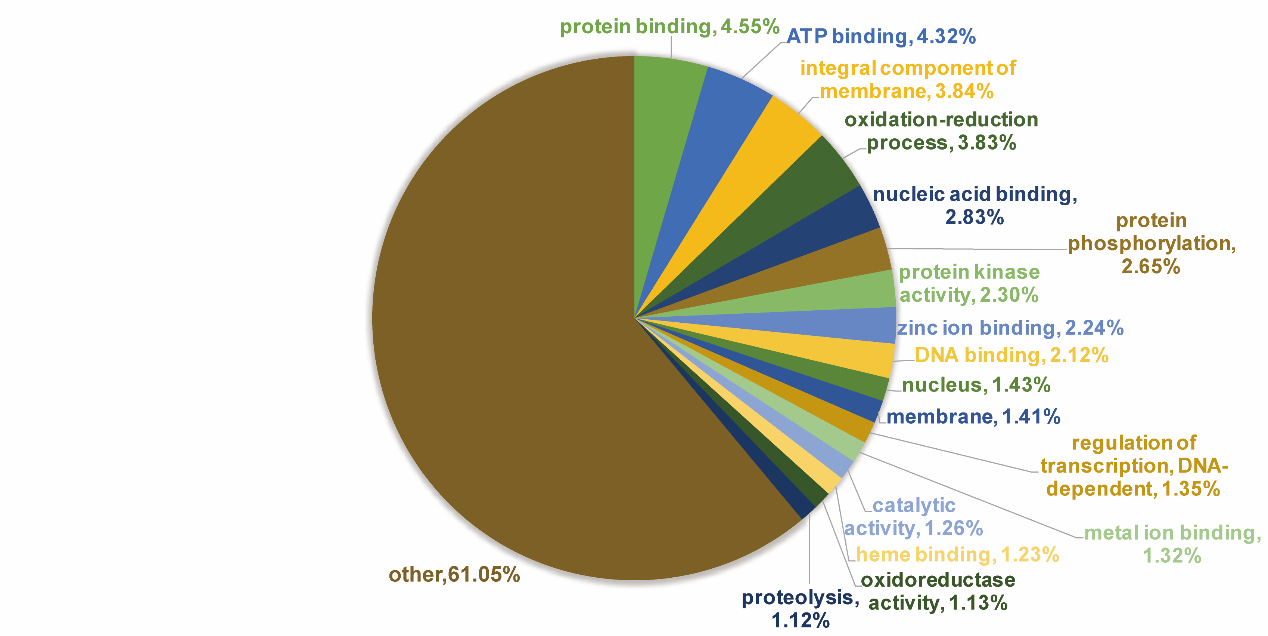


## Supplemental Figure 5. Gene ontology distribution of *S. aralocaspica* protein coding genes.

The GO terms of genes were union set of blast2go annotations of NR blast results and interpro go annotations without top level categories – biological process, molecular function, cellular component.


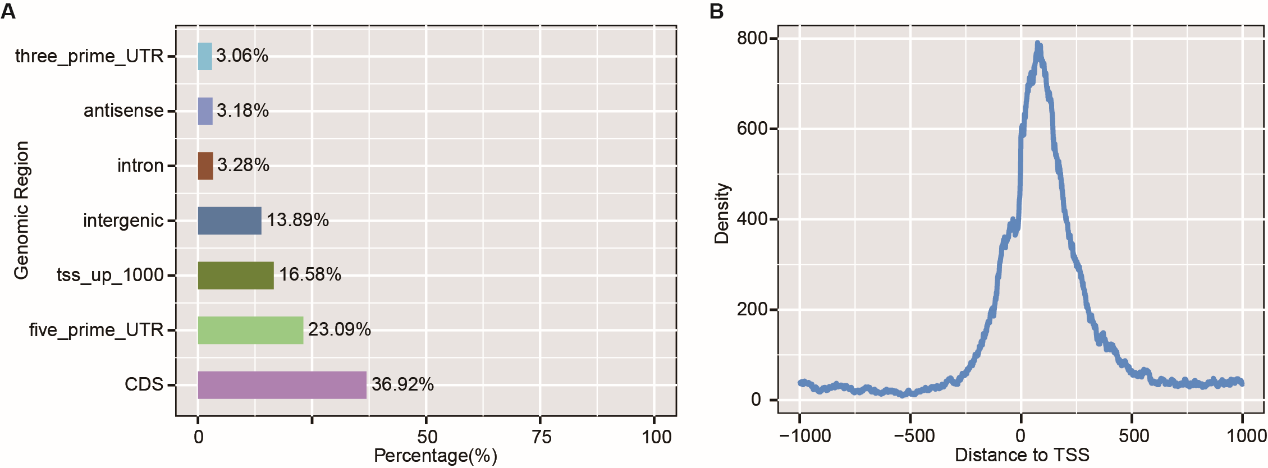


## Supplemental Figure 6. Transcription start site (TSS) annotation with Cage-seq.

**A,** distribution of identified transcription start site clusters (TCs) in different genomic regions, tss_up_1000 indicates upstream 0-1000 bp of TSS. **B,** density distribution of identified TCs around TSS.


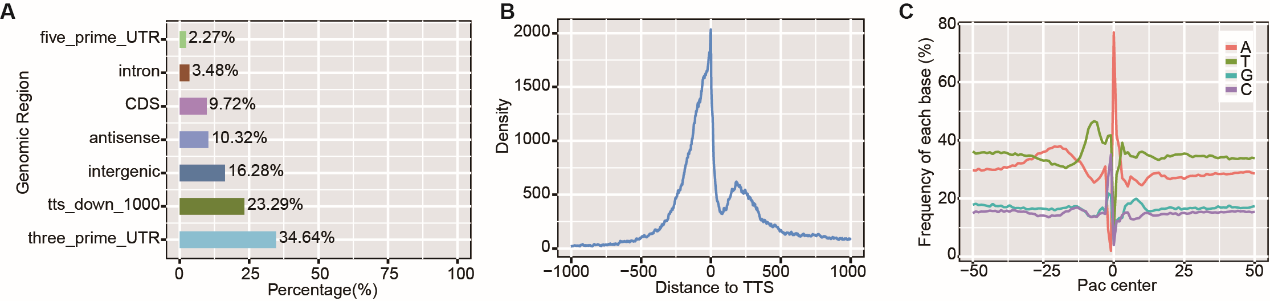


## Supplemental Figure 7. Transcription terminal site (TTS) annotation with Pas-seq.

**A,** Distribution of identified polyA site clusters (PACs) in different genomic regions, tts_down_1000 indicates downstream 0-1000 bp of TTS. **B,** Density distribution of identified PACs around TTS. **C,** frequency of each base around summit of PACs.


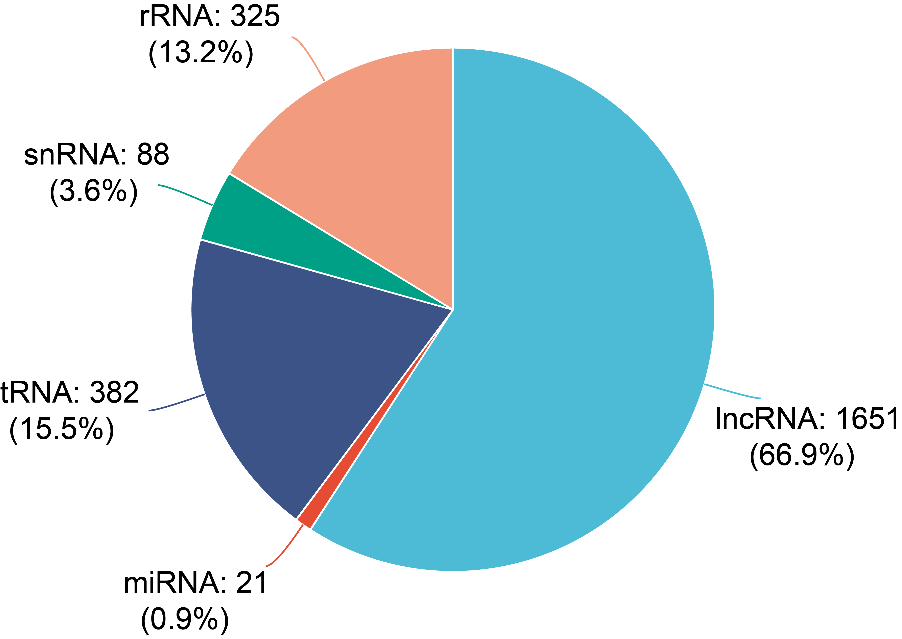


## Supplemental Figure 8. Non-coding RNAs classification in *S. aralocaspica*.

# Supplementary Tables

## Supplemental Table 1. Summary of sequencing data obtained for genome assembly.

| **Libraries** | **Type** | **Insert size** (mean/SD) | **Read length (bp)** | **Raw data (Gb)** | **Clean data (Gb)** | **Coverage (🞨)** |
| --- | --- | --- | --- | --- | --- | --- |
| **PCR** | Paired end | 350bp (331/29) | 100 | 46 | 46 | 99 |
|  |  | 500bp (471/29) | 100 | 46 | 46 | 99 |
|  |  | 800bp (766/38) | 100 | 46 | 46 | 99 |
|  | Mate pair | 2kb (1979/428) | 81 | 71 | 24 | 51 |
|  |  | 5kb (4626/1835) | 81 | 82 | 18 | 39 |
|  |  | 10kb (10,807/1,341) | 81 | 39 | 7.4 | 16 |
|  |  | 20kb (17,367/3,881) | 81 | 40 | 8 | 17 |
| **PacBio** |  | / | >1k | 10 | 6.9 | 15 |
| **Total** |  | / | / | 380 | 202.3 | 433 |

## Supplemental Table 2. The assembly statistics of the *S. aralocaspica* genome.

| **-** | **Contig** | | **Scaffold** | | | | **PBjelly assemblys** | |
| --- | --- | --- | --- | --- | --- | --- | --- | --- |
|  | **Size(bp)** | **Number** | **Size(bp)** | **Number** | | **Size(bp)** | | **Number** |
| **N90** | 143 | 263,809 | **306,619** | | 332 | 363,870 | | 283 |
| **N80** | 2,670 | 11,242 | 558,320 | | 231 | 690,729 | | 195 |
| **N70** | 18,589 | 5,445 | 752,872 | | 167 | 939,454 | | 139 |
| **N60** | 33,975 | 3,606 | 984,237 | | 117 | 1,271,602 | | 97 |
| **N50** | 49,212 | 2,464 | 1,437,169 | | 80 | 1,831,350 | | 68 |
| **Longest** | 653,049 | - | 9,293,200 | | - | 9,979,223 | | - |
| **Total Size** | - | - | 424,456,026 | | - | 451,707,677 | | - |
| **Total Number(**≥ **500 bp)** | - | 17,302 | - | | 4,184 | - | | 4,033 |
| **Total Number(**≥ **2 kb)** | - | 11,800 | - | | 1,482 | - | | 1,493 |
| **Length of Ns** | - | - | 25,118,675 | | - | 13,456,548 | | - |

## Supplemental Table 3. Information of different types of RNA libraries.

| **Species** | **Tissue** | **Short name^a^** | **Library** | **Fragment size** | **Average reads length(bp)** | **Total clean data(Gb)** |
| --- | --- | --- | --- | --- | --- | --- |
|  |  |  |  | **(bp)** |  |  |
| *S. aralocaspica* | mix-tissue | MIX | RNA | 200-500 | 150 | 30 |
|  | mature leaf | ML | CAGE | 200-500 | 150 | 16 |
|  | mature leaf | ML | PAS | 200-500 | 300 | 28.5 |
|  | mix-tissue | MIX | sRNA | 350 | 36 | 4.5 |

^a^ Short name will be used in following analysis.

## Supplemental Table 4. Mapping efficiency of short insert library reads

| DNA paired end insert | Total reads | Reads mapped | Reads mapped and paired | Reads mapped and properly paired |
| --- | --- | --- | --- | --- |
| 350bp | 204199236 | 200068366 (97.98%) | 198096674 (97.01%) | 185064708 (90.63%) |
| 500bp | 202874264 | 198740654 (97.96) | 196570100 (96.89%) | 181154712 (89.29%) |
| 800bp | 205135304 | 201109346 (98.04%) | 198953352 (96.99%) | 178626536 (87.08%) |

## Supplemental Table 5. Assessment of sequence coverage of S. *aralocaspica* genome assembly using unigenes.

Unigenes were assembled using mix-tissues RNA-seq reads and aligned to the genome assembly with BLAST.

| **Unigene** | | | | | | | |
| --- | --- | --- | --- | --- | --- | --- | --- |
| Dataset | Number | Total  length (bp) | Covered by assembly (%) | With >90% Sequence in one Scaffold | | With >50% Sequence in one Scaffold | |
|  |  |  |  | Number | Percentage (%) | Number | Percentage (%) |
| All | 157,521 | 94,968,978 | 94.68 | 120,114 | 76.25 | 137,123 | 87.05 |
| >200bp | 157,521 | 94,968,978 | 94.68 | 120,114 | 76.25 | 137,123 | 87.05 |
| >500bp | 52,467 | 63,636,653 | 98.61 | 46,720 | 89.05 | 50,224 | 95.72 |
| >1 kb | 24,576 | 44,700,526 | 99.48 | 22,800 | 92.77 | 24,023 | 97.75 |

## Supplemental Table 6. Gene prediction in the *S. aralocaspica* genome.

| **Gene Set** | | **Number** | **Average Transcript Length (bp)** | **Average CDS Length (bp)** | **Average Exon Number**  **per Gene** | **Average Exon Length (bp)** | **Average Intron Length (bp)** |
| --- | --- | --- | --- | --- | --- | --- | --- |
| ***De novo*** | **AUGUSTUS** | 38,591 | 4,344.65 | 1,032.34 | 4.37 | 236.08 | 982.04 |
|  | **GlimmerHMM** | 37,356 | 1,767.41 | 744.40 | 3.17 | 234.68 | 471.01 |
|  | ***GeneMark-ES*** | 40,149 | 4,985.77 | 1,110.49 | 6.04 | 183.89 | 769.07 |
| **Homolog** |  | 31,646 (30,854) | 3,153.98 | 902.39 | 3.13 | 287.93 | 1,055.10 |
| **RNA_Seq** | ***PASA*** | 69,695 | 2,121.60 | 716.45 | 2.46 | 291.24 | 962.41 |
| **Combiner** | **EVM** | 38,061 | 2,800.90 | 933.55 | 3.90 | 239.40 | 644.03 |
|  | **MAKER** | 29,604 | 4,462.03 | 1,112.95 | 4.76 | 234.03 | 891.76 |
|  |  |  |  |  |  |  |  |

## Supplemental Table 7. Comparison of the gene structure among *S. aralocaspica* and some other species

|  | ***S.Aralocaspica*** | **C. quinoa** | ***B. vulgaris*** | ***A.*** ***thaliana*** | ***Glycine max*** | ***Populus italica*** |
| --- | --- | --- | --- | --- | --- | --- |
| Genome assembly size* (Mb) | 452 | 1,484 | 731.3 | 119.48 | 955.05 | 403.75 |
| # Genes | 29,604 | 63,089 | 32,734 | 26,637 | 55,787 | 45,033 |
| # Exons | 140,915 | 346,989 | 184,302 | 139,382 | 331,060 | 224,259 |
| # Introns | 111,311 | 283,900 | 151,231 | 112,745 | 275,273 | 179,226 |
| Mean exon per gene | 4.76 | 5.50 | 5.62 | 5.23 | 5.93 | 4.98 |
| Mean exon length (bp) | 234.03 | 242.87 | 245.47 | 237.50 | 206.26 | 231.14 |
| Mean CDS length (bp) | 1,112.95 | 1,334.99 | 1,378.79 | 1242.78 | 1224.01 | 1151.06 |
| Mean intron length (bp) | 891.76 | 1,014.38 | 1,397.95 | 157.54 | 423.71 | 347.09 |
| Mean transcripts length (bp) | 4,462.03 | 5,896.33 | 7,833.14 | 1909.57 | 3816.24 | 2916.61 |

*：Without NNs;

## Supplemental Table 8. Summary of *S. aralocaspica* gene annotation based on homology or functional classification.

|  | | **Number** | **Percentage*** |
| --- | --- | --- | --- |
| **Total** | | 29,604 | - |
| **Annotated** | InterPro | 24,634 | 83.2% |
|  | GO | 18,548 | 62.6% |
|  | KEGG | 6,714 | 22.6% |
|  | Swissprot | 15,838 | 53.4% |
|  | NR | 27,750 | 93.7% |
|  | Total | 28,781 | 97.2% |
| **Unannotated** | | 823 | 2.8% |
| * Percentage of the annotated nuclear genes. | | | |

## Supplemental Table 9. Number of *S. aralocaspica* genes with protein or unigene support.

|  | **Number** | **Percentage** |
| --- | --- | --- |
| **Total genes** | 29,604 | **100%** |
| **Genes with:** |  |  |
| **Protein Support^a^** | 27,111 | 91.57% |
| **Unigene Support^b^** | 26,963 | 91.07% |
| ***Ab Initio*** | 25,279 | 85.39% |
| **Protein & Unigene & Ab Initio Support** | 22,517 | 76.06% |

^a^ Protein database: KEGG, Swiss-Prot, NR, InterproScan; Protein support criteria: identity ≥ 30%, e value < 1 e-5.

^b^ RNA-Seq clean reads wwere assembled into unigenes by Trinity. GeneMark and GlimmerHmm were used for *ab initio* prediction, genes support criteria: identity ≥ 30%, e value < 1 e-5.

## Supplemental Table 10. Noncoding RNA genes in the *S. aralocaspica* genome.

| **Type** | **Number** | **Average Length (bp)** |
| --- | --- | --- |
| **lncRNA** | 1651 | 3717.5 |
| **miRNA** | 21 | 102.1 |
| **tRNA** | 382 | 74.5 |
| **snRNA** | 88 | 98.4 |
| **rRNA** | 325 | 144.8 |

## Supplemental Table 11. Repeat elements in the *S. aralocaspica* genome. Repeat elements were identified by different methods and then combined into a final repeat set.

|  | **RepBase TEs** | | ***De novo*** | | **Combined TEs** | | |
| --- | --- | --- | --- | --- | --- | --- | --- |
|  | **Length (bp)** | **% in genome** | **Length**  **(bp)** | **% in genome** | | **Length**  **(bp)** | **% in genome** |
| **DNA** | 5,090,733 | 1.13 | 6,448,526 | 1.43 | | 10,605,723 | 2.35 |
| **LINE** | 3,010,864 | 0.67 | 2,675,538 | 0.59 | | 4,295,125 | 0.95 |
| **LTR** | 44,129,761 | 9.77 | 71,814,812 | 15.90 | | 84,140,487 | 18.63 |
| **SINE** | 14,591 | 0.00 | 0 | 0 | | 14,591 | 0.00 |
| **Other** | 17,772,295 | 3.94 | 18,926,333 | 4.05 | | 21,786,124 | 4.82 |
| **Unknown** | 297,884 | 0.06 | 56,195,195 | 12.44 | | 52,654,862 | 11.66 |
| **Total** | 70,316,128 | 15.57 | 155,440,522 | 34.41 | | 173,496,912 | 38.41 |

LINE, long interspersed nuclear elements; SINE, short interspersed nuclear elements; LTR, long terminal repeats; DNA, DNA transposon; Other, including Tandem repeats.

## Supplemental Table 12. Repeat elements in *S. aralocaspica* genomes.

|  | ***S. aralocaspica*** | |
| --- | --- | --- |
| **Type** | **Length (bp)** | **% in genome** |
| **Genome size** | 451,707,677 | / |
| **DNA** | 10,605,723 | 2.35 |
| **LINE** | 4,295,125 | 0.95 |
| **LTR** | 84,140,487 | 18.63 |
| **SINE** | 14,591 | 0.00 |
| **Other** | 21,786,124 | 4.82 |
| **Unknown** | 52,654,862 | 11.66 |
| **Total** | 173,496,912 | 38.41 |

LINE, long interspersed nuclear elements; SINE, short interspersed nuclear elements; LTR, long terminal repeats; DNA, DNA transposon; Other, including Tandem repeats.

##

## Supplemental Table 13. Orthogroups clustered by OrthoFinder in 18 species.

|  | **Number of genes** | **Number of genes in orthogroups** | **Number of unassigned genes** | **Percentage of genes in orthogroups** | **Percentage of unassigned genes** | **Number of orthogroups containing species** | **Percentage of orthogroups containing species** | **Number of species-specific orthogroups** | **Number of genes in species-specific orthogroups** | **Percentage of genes in species-specific orthogroups** |
| --- | --- | --- | --- | --- | --- | --- | --- | --- | --- | --- |
| *Amaranthus hypochondriacus* | 23843 | 21069 | 2774 | 88.4 | 11.6 | 11605 | 60.1 | 20 | 76 | 0.3 |
| *Ananas comosus* | 21577 | 20263 | 1314 | 93.9 | 6.1 | 11092 | 57.4 | 24 | 139 | 0.6 |
| *Beta vulgaris* | 23944 | 22738 | 1206 | 95 | 5 | 11690 | 60.5 | 21 | 82 | 0.3 |
| *Chenopodium quinoa* | 48270 | 43888 | 4382 | 90.9 | 9.1 | 12305 | 63.7 | 74 | 490 | 1 |
| *Zea Mays* | 36457 | 31290 | 5167 | 85.8 | 14.2 | 12729 | 65.9 | 26 | 169 | 0.5 |
| *Musa acuminata* | 29997 | 28708 | 1289 | 95.7 | 4.3 | 11000 | 56.9 | 18 | 54 | 0.2 |
| *Oryza sativa* | 27999 | 23224 | 4775 | 82.9 | 17.1 | 12364 | 64 | 5 | 13 | 0 |
| *Phalaenopsis equestris* | 19771 | 18595 | 1176 | 94.1 | 5.9 | 10471 | 54.2 | 17 | 43 | 0.2 |
| *Panicum_hallii* | 25685 | 24903 | 782 | 97 | 3 | 12803 | 66.3 | 7 | 43 | 0.2 |
| *Physcomitrella patens* | 32234 | 17529 | 14705 | 54.4 | 45.6 | 7958 | 41.2 | 204 | 1015 | 3.1 |
| *Pennisetum glaucum* | 38579 | 27783 | 10796 | 72 | 28 | 13079 | 67.7 | 52 | 394 | 1 |
| *Saccharum spp.* | 25316 | 20078 | 5238 | 79.3 | 20.7 | 10146 | 52.5 | 14 | 68 | 0.3 |
| ***S. aralocaspica*** | **29604** | **23112** | **6492** | **78.1** | **21.9** | **11768** | **60.9** | **70** | **351** | **1.2** |
| *Setaria italica* | 26938 | 26150 | 788 | 97.1 | 2.9 | 12928 | 66.9 | 5 | 20 | 0.1 |
| *Sorghum bicolor* | 27495 | 26103 | 1392 | 94.9 | 5.1 | 13059 | 67.6 | 10 | 42 | 0.2 |
| *Solanum tuberosum* | 39020 | 26479 | 12541 | 67.9 | 32.1 | 10915 | 56.5 | 121 | 1070 | 2.7 |
| *Spinacia oleracea* | 25196 | 23941 | 1255 | 95 | 5 | 11752 | 60.8 | 31 | 145 | 0.6 |
| *Athaliana thaliana* | 27442 | 23046 | 4396 | 84 | 16 | 10948 | 56.7 | 59 | 479 | 1.7 |
