# Supplementary material for: A draft genome assembly of halophyte Suaeda aralocaspica, a plant that performs C4 photosynthesis within individual cells
Source: Gigascience. 2019 Sep 12;8(9):giz116. doi: 10.1093/gigascience/giz116 (PMC6741815; doi:10.1093/gigascience/giz116)
Supplement: giz116_Response_to_Reviewer_Comments_Original_Submission [file giz116_response_to_reviewer_comments_original_submission.pdf]

Author's Response To Reviewer Comments

Close

July 12, 2019

Dear Editor,

We are pleased to submit the revised manuscript entitled "A draft genome assembly of halophyte Suaeda aralocaspica, a plant that performs C_4 photosynthesis within individual cells". Attached please find our detailed responses to your comments and those raised by both reviewers. The manuscript has been revised accordingly. All authors greatly appreciate the critical comments which we have used to guide our revisions to improve the manuscript.

We believe the quality and clarity of the manuscript has been improved significantly, which we hope is now in a form suitable for publication in GigaScience as a Data Note.

Yours sincerely,
Dr. Yi Zhang
Center for Genome Analysis, ABLife Inc., Wuhan, Hubei 430075, China
Email: yizhang@ablife.cc

Comments from the editor and responses:

As you will see the reviewers felt significant additional work is required to improve the writing, methodological detail and validation/analysis. We are willing to continue to consider the manuscript if you are able to address these points, but we will only send it back to the reviewers if you are able to carry out this additional work thoroughly. This includes getting a native English speaker or commercial copy editor to improve the language. And improve the methodological detail, for example including detailed protocols via protocols.io (these can be reused or adapted from protocols already submitted https://www.protocols.io/groups/gigascience-journal).

Response: According the your kind advice, we have sent our revised manuscript to a commercial copy editor in International Science Editing (http://www.internationalscienceediting.com) to improve the language. In addition, we have updated the methodological details in manuscript and also uploaded the detailed commands and house-scripts used in genome assembly and annotation to GigaDB database.

Responses to the reviewers' comments are detailed below.

Comments from the reviewers and responses:

Reviewer #1: Wang et al. generate a draft genome of Suaeda aralocaspica, a halophyte with single cell C_4 photosynthesis. They use a primarily Illumina based approach with gap filling using low coverage PacBio data. This resource will be useful for the comparative genomics and C_4 research communities. I have some concerns I feel should be addressed before this manuscript is suitable for publication.

Major:
Were the gaps that were filled by PacBio data polished? Raw PacBio reads have an error rate of ~5-10% and these patched regions would be full of errors if there was no polishing. The Raw PacBio reads could be polished against themselves prior to gap filling with PBJelly or the regions could be polished with Illumina data using a program such as Pilon.

Response: We appreciate the reviewer's comment. Sorry that we missed this important information in the last version of the manuscript. The details have been added to the revised manuscript (lines 121-

124). In this study, raw PacBio reads were polished with Illumina data using Proovread (https://github.com/BioInf-Wuerzburg/proovread) to ensure the high accuracy of the PacBio reads used for genome assembly.

Minor:
Figure 2 is not particularly informative and slightly misleading. Circos plots of chromosomes/pseudomolecules can be useful for looking at gene and repeat density as well as macrosynteny (for polyploids and for detecting whole genome duplication events). Here, the 4,033 contigs are stitched together into one continuous block and various features are highlighted. Because most scaffolds are relatively small, rolling windows of features seem meaningless (particularly track i).

Response: We concur with the reviewer’s comment, and decided to remove this figure from the revised manuscript accordingly.

Line 205: more details on what MAKER parameters were used are needed, particularly which gene prediction programs were used (i.e. Snap, Augustus, etc). The BUSCO completeness of the annotation should also be provided.

Response: Thanks for the reviewer’s comment, and detailed MAKER parameters were added to revised manuscript (lines 224-231) accordingly. De novo predictions were processed by AUGUSTUS (v3.2.1). The BUSCO completeness of the annotation was provided at Lines 212-214.

I would caution the authors against using 'first' in the title. To my knowledge, this is the first plant with single cell C_4 to be sequenced, but that might not be the case when this paper is published.

Response: Thanks for this specific comment. The title was changed to: “A draft genome assembly of halophyte Suaeda aralocaspica, a plant that performs C_4 photosynthesis within individual cells”.

Reviewer #2: Gigascience 4.2019
Here, the authors present the wet lab work, some bioinformatics information and short analysis of the genome of S. aralocaspica, the first SCC_4 genome available. This is a short technical / data report of the public availability of a genome. I appreciate the simplicity this type of article; however, I have a number of scientific and data presentation comments. These are detailed below.

Genome analysis:
The genome analyses (phylogenetics, gene families) leave much to be desired. They are not well described, interpretation is nearly completely lacking and the scope of the analyses are very narrow. I understand that this is a data note; however, if such analyses are going to be conducted, they should be done in a rigorous manner, with sufficient replication, and include a complete methodological description and scientific interpretation.
What does 'SCC_4 would independently evolve in this family' mean? Is it that the trees indicate that SCC_4 is a derived trait in the family? If so, to make this claim, you would need to analyze gene / species trees with many more species. It could easily be that the observed pattern is simply due to very small sample size.
Same goes for the private-gene family analysis. If the authors want to actually perform this analysis they need to add many more genomes. This is in part to capture the evolution of these families, and in part because sequence alignments of highly diverged and sparsely sampled sequences are highly biased and inaccurate.

Response: Many thanks for your critical comments. We have re-performed the comparative genome analysis, using all annotated protein sequences from 18 sequenced plant genomes including eight C_3 species, eight C_4 species and two CAM species. The analysis We also rewrote the result presentation under the subtitle of “Phylogenetic placement of S. aralocaspica” accordingly. In fact, we tried to use more species, however, which led to no single copy gene for analysis. In the revised version, we also added a couple of interpretation and expanded the scope of the analyses.

Text:
There are a lot of proofreading and grammar errors. The most obvious is line 282: 'We annotated xxx protein-coding genes and noncoding genes' … This type of error is very concerning and makes me

skeptical that the authors put in the effort to check accuracy of the numbers presented throughout.
Response: To avoid this type of errors, we have sent our revised manuscript to a commercial copy editor in International Science Editing (http://www.internationalscienceediting.com) to improve the language.

The title is misleading and makes the reader think that you have sequenced a unicellular organism.
Response: Thanks for your comment. We have change the title to : "A draft genome assembly of halophyte Suaeda aralocaspica, a plant that performs C_4 photosynthesis within individual cells" to avoid the misunderstanding.

The term 'high quality' is used a lot. To me, this is not helpful. High-quality means different things to different people. The genome presented here is of low or moderate quality to those working in model systems, or those with recently sequenced genomes. However, it is certainly high enough quality for a species with little genomic information. I would drop this term throughout and instead focus on the exact contiguity.
Response: Thanks for you for your comments. We have dropped this term throughout..

Methods:
The methodological details focus on lab protocols, but have very little information about sample sizes, plant growth, etc. for example: How were plants grown for RNA extraction? How many biological replicates? N. technical replicates? Was RNA extracted from the exact same plant as gDNA? If not, was it the same genotype? If so, how was it clonally propagated? All of these questions and many others must be answered.
Please provide georeferenced location information for the sample collection site.
Please provide the collection permits and all other information about how the sample was obtained.

Response: Thanks for you for your comments. We have added the suggested information as a separated part called "Plant materials" at line 92 in revised manuscript.

How were PacBio reads error corrected?

Response: The raw reads with length < 1kb were filtered and then 46 Gb of Illumina clean reads with 100 bp read length was used to correct the PacBio raw reads using Proovread (https://github.com/BioInf-Wuerzburg/proovread). The detailed method were added to the revised manuscript (line 121).

How is % gap calculated? on a per-bp basis? Line 126. If so, how large are the gaps (10k Ns?)

Response: A "gap" is a run of Ns of any length, % gap was calculated using total length of Ns divided to the total length of assembly.
In the final assembly, there are 35949 Gaps, the total length of Gaps is 13456548, in average each gap is 374 Ns. The results were added to the revised manuscript (line 148).

Close
