# Supplementary material for: A draft genome assembly of halophyte Suaeda aralocaspica, a plant that performs C4 photosynthesis within individual cells
Source: Gigascience. 2019 Sep 12;8(9):giz116. doi: 10.1093/gigascience/giz116 (PMC6741815; doi:10.1093/gigascience/giz116)
Supplement: giz116_Response_to_Reviewer_Comments_Revision_1 [file giz116_response_to_reviewer_comments_revision_1.pdf]

Author's Response To Reviewer Comments

Close

August 8, 2019

Dear Editor Zhou,

Thank you so much for bring us the good news regarding the submitted manuscript "A draft genome assembly of halophyte Suaeda aralocaspica, a plant that performs C_4 photosynthesis within individual cells". Attached please find our responses to your comment and the one raised by reviewer #2. The manuscript and figure 2 have been revised accordingly.

Yours sincerely,
Dr. Yi Zhang
Center for Genome Analysis, ABLife Inc., Wuhan, Hubei 430075, China
Email: yizhang@ablife.cc

Comments from the editor and responses:

In addition, please register any new software application in the SciCrunch.org database to receive a RRID (Research Resource Identification Initiative ID) number, and include this in your manuscript. This will facilitate tracking, reproducibility and re-use of your tool.

Response: According the your kind advice, we have registered new software application in the SciCrunch.org database and added the RRID number in our revised manuscript.

Comments from the reviewers and responses:

Reviewer reports:
Reviewer #2: The authors have addressed my previous comments. However, I have several issues with the new phylogenetic analysis ...
Is the point that Suaeda is a sister group to the rest of the amaranths (line 284)? If so, this tree absolutely needs bootstrap support at the nodes to show that you can distinguish positions in the amaranths. Furthermore, if the goal is to understand evolution in the amaranths, why are there grasses in this tree at all? You would find far more single-copy loci and build a better phylogeny if you just look at the amaranths and adjacent families. Also, all findings presented in lines 285-294 need statistics showing that in fact the patterns you observe are different from a random tree. I think presenting % bootstrap support in the text along with the tree would accomplish this.

Response: Many thanks for your comments and advice. Yes, according to the phylogenetic analysis, Suaeda is a sister group to the rest of the Amaranthaceae. We have added % bootstrap support at the nodes. We have two goals for this analysis. One goal is to understand the evolution in the Amaranthaceae. The other goal is to explore the photosynthesis-related phylogenetic placement of Suaeda in C_3 and C_4 plants, which are not limited to Amaranthaceae. We therefore selected some representative non-Amaranthaceae C_3 and C_4 plants for phylogenetic analysis.

Close
