# Supplementary material for: A draft genome assembly of halophyte Suaeda aralocaspica, a plant that performs C4 photosynthesis within individual cells
Source: Gigascience. 2019 Sep 12;8(9):giz116. doi: 10.1093/gigascience/giz116 (PMC6741815; doi:10.1093/gigascience/giz116)
Supplement: giz116_Reviewer_1_Report_Original_Submission — Robert van Buren -- 3/19/2019 Reviewed [file giz116_reviewer_1_report_original_submission.pdf]

Reviewer Report

Title: A draft genome assembly of halophyte Suaeda aralocaspica, a plant that performs Câ„ photosynthesis within individual cells

Version: Original Submission **Date:** 3/19/2019

Reviewer name: Robert van Buren

Reviewer Comments to Author:

Wang et al. generate a draft genome of Suaeda aralocaspica, a halophyte with single cell C4 photosynthesis. They use a primarily Illumina based approach with gap filling using low coverage PacBio data. This resource will be useful for the comparative genomics and C4 research communities. I have some concerns I feel should be addressed before this manuscript is suitable for publication.
Major:
Were the gaps that were filled by PacBio data polished? Raw PacBio reads have an error rate of ~5-10% and these patched regions would be full of errors if there was no polishing. The Raw PacBio reads could be polished against themselves prior to gap filling with PBJelly or the regions could be polished with Illumina data using a program such as Pilon.
Minor:
Figure 2 is not particularly informative and slightly misleading. Circos plots of chromosomes/pseudomolecules can be useful for looking at gene and repeat density as well as macrosynteny (for polyploids and for detecting whole genome duplication events). Here, the 4,033 contigs are stitched together into one continuous block and various features are highlighted. Because most scaffolds are relatively small, rolling windows of features seem meaningless (particularly track i).
Line 205: more details on what MAKER parameters were used are needed, particularly which gene prediction programs were used (i.e. Snap, Augustus, etc). The BUSCO completeness of the annotation should also be provided.
I would caution the authors against using 'first' in the title. To my knowledge, this is the first plant with single cell C4 to be sequenced, but that might not be the case when this paper is published.

Level of Interest

Please indicate how interesting you found the manuscript: Choose an item.

Quality of Written English

Please indicate the quality of language in the manuscript: Choose an item.

Declaration of Competing Interests

Please complete a declaration of competing interests, considering the following questions:

- Have you in the past five years received reimbursements, fees, funding, or salary from an organisation that may in any way gain or lose financially from the publication of this manuscript, either now or in the future?
- Do you hold any stocks or shares in an organisation that may in any way gain or lose financially from the publication of this manuscript, either now or in the future?
- Do you hold or are you currently applying for any patents relating to the content of the manuscript?
- Have you received reimbursements, fees, funding, or salary from an organization that holds or has applied for patents relating to the content of the manuscript?
- Do you have any other financial competing interests?
- Do you have any non-financial competing interests in relation to this paper?

If you can answer no to all of the above, write 'I declare that I have no competing interests' below. If your reply is yes to any, please give details below.

I declare that I have no competing interests

I agree to the open peer review policy of the journal. I understand that my name will be included on my report to the authors and, if the manuscript is accepted for publication, my named report including any attachments I upload will be posted on the website along with the authors' responses. I agree for my report to be made available under an Open Access Creative Commons CC-BY license (http://creativecommons.org/licenses/by/4.0/). I understand that any comments which I do not wish to be included in my named report can be included as confidential comments to the editors, which will not be published.

Choose an item.

To further support our reviewers, we have joined with Publons, where you can gain additional credit to further highlight your hard work (see: https://publons.com/journal/530/gigascience). On publication of this paper, your review will be automatically added to Publons, you can then choose whether or not to claim your Publons credit. I understand this statement.

Yes Choose an item.
