# Supplementary material for: A draft genome assembly of halophyte Suaeda aralocaspica, a plant that performs C4 photosynthesis within individual cells
Source: Gigascience. 2019 Sep 12;8(9):giz116. doi: 10.1093/gigascience/giz116 (PMC6741815; doi:10.1093/gigascience/giz116)
Supplement: giz116_Reviewer_2_Report_Original_Submission — John Lovell -- 4/9/2019 Reviewed [file giz116_reviewer_2_report_original_submission.pdf]

Reviewer Report

Title: A draft genome assembly of halophyte Suaeda aralocaspica, a plant that performs Câ„„ photosynthesis within individual cells

Version: Original Submission **Date:** 4/9/2019

Reviewer name: John Lovell

Reviewer Comments to Author:

Gigascience 4.2019
Here, the authors present the wet lab work, some bioinformatics information and short analysis of the genome of S. aralocaspica, the first SCC4 genome available. This is a short technical / data report of the public availability of a genome. I appreciate the simplicity this type of article; however, I have a number of scientific and data presentation comments. These are detailed below.
--
Genome analysis:
The genome analyses (phylogenetics, gene families) leave much to be desired. They are not well described, interpretation is nearly completely lacking and the scope of the analyses are very narrow. I understand that this is a data note; however, if such analyses are going to be conducted, they should be done in a rigorous manner, with sufficient replication, and include a complete methodological description and scientific interpretation.
What does 'SCC4 would independently evolve in this family' mean? Is it that the trees indicate that SCC4 is a derived trait in the family? If so, to make this claim, you would need to analyze gene / species trees with many more species. It could easily be that the observed pattern is simply due to very small sample size.
Same goes for the private-gene family analysis. If the authors want to actually perform this analysis they need to add many more genomes. This is in part to capture the evolution of these families, and in part because sequence alignments of highly diverged and sparsely sampled sequences are highly biased and inaccurate.
Text:
There are a lot of proofreading and grammar errors. The most obvious is line 282: 'We annotated xxx protein-coding genes and noncoding genes' … This type of error is very concerning and makes me skeptical that the authors put in the effort to check accuracy of the numbers presented throughout.
The title is misleading and makes the reader think that you have sequenced a unicellular organism.
The term 'high quality' is used a lot. To me, this is not helpful. High-quality means different things to different people. The genome presented here is of low or moderate quality to those working in model systems, or those with recently sequenced genomes. However, it is certainly high enough quality for a species with little genomic information. I would drop this term throughout and instead focus on the exact contiguity.
Methods:
The methodological details focus on lab protocols, but have very little information about sample sizes,

plant growth, etc. for example: How were plants grown for RNA extraction? How many biological replicates? N. technical replicates? Was RNA extracted from the exact same plant as gDNA? If not, was it the same genotype? If so, how was it clonally propagated? All of these questions and many others must be answered.
Please provide georeferenced location information for the sample collection site.
Please provide the collection permits and all other information about how the sample was obtained.
How were PacBio reads error corrected?
How is % gap calculated? on a per-bp basis? Line 126. If so, how large are the gaps (10k Ns?)

Level of Interest

Please indicate how interesting you found the manuscript: Choose an item.

Quality of Written English

Please indicate the quality of language in the manuscript: Choose an item.

Declaration of Competing Interests

Please complete a declaration of competing interests, considering the following questions:

- Have you in the past five years received reimbursements, fees, funding, or salary from an organisation that may in any way gain or lose financially from the publication of this manuscript, either now or in the future?
- Do you hold any stocks or shares in an organisation that may in any way gain or lose financially from the publication of this manuscript, either now or in the future?
- Do you hold or are you currently applying for any patents relating to the content of the manuscript?
- Have you received reimbursements, fees, funding, or salary from an organization that holds or has applied for patents relating to the content of the manuscript?
- Do you have any other financial competing interests?
- Do you have any non-financial competing interests in relation to this paper?

If you can answer no to all of the above, write 'I declare that I have no competing interests' below. If your reply is yes to any, please give details below.

I declare that I have no competing interests

I agree to the open peer review policy of the journal. I understand that my name will be included on my report to the authors and, if the manuscript is accepted for publication, my named report including any attachments I upload will be posted on the website along with the authors' responses. I agree for my

report to be made available under an Open Access Creative Commons CC-BY license (http://creativecommons.org/licenses/by/4.0/). I understand that any comments which I do not wish to be included in my named report can be included as confidential comments to the editors, which will not be published.

Choose an item.

To further support our reviewers, we have joined with Publons, where you can gain additional credit to further highlight your hard work (see: https://publons.com/journal/530/gigascience). On publication of this paper, your review will be automatically added to Publons, you can then choose whether or not to claim your Publons credit. I understand this statement.

Yes Choose an item.
